# Electronically flexible PYA ligands for efficient palladium-catalyzed α-arylation of ketones†

**DOI:** 10.1039/d3dt03182a

**Published:** 2023-10-18

**Authors:** Esaïe Reusser, Martin Albrecht

**Affiliations:** a Department of Chemistry, Biochemistry and Pharmaceutical Sciences, University of Bern Freiestrasse 3 3012 Bern Switzerland martin.albrecht@unibe.ch

## Abstract

Palladium-catalyzed cross-coupling chemistry and in particular ketone α-arylation has been relying on a rather narrow range of supporting ligands with almost no alternatives to phosphines and N-heterocyclic carbenes. Here we introduce a class of well-defined palladium(ii) complexes supported by *N*,*N*′-chelating and electronically flexible pyridylidene amide (PYA)-pyridyl ligands as catalysts for efficient α-arylation of ketones. Steric and electronic variations of the *N*,*N*′-bidentate ligand indicate that the introduction of an *ortho*-methyl group on the pyridinum heterocycle of the PYA ligand enhances the arylation rate and prevents catalyst deactivation, reaching turnover numbers up to 7300 and turnover frequencies of almost 10 000 h^−1^, which is similar to that of the best phosphine complexes known to date. Introducing a shielding xylyl substituent accelerates catalysis further, however at the expense of lower selectivity towards arylated ketones. Substrate scope investigations revealed that both electron-rich and -poor aryl bromides as well as a broad range of electronically and sterically modified ketones are efficiently converted, including aliphatic ketones. Mechanistic investigations using Hammett and Eyring analyses indicated that both, oxidative addition and reductive elimination are relatively fast, presumably as a consequence of the electronic flexibility of the PYA ligand, while enolate coordination was identified as the turnover-limiting step.

## Abstract 2

Electronically flexible PYA ligands enable efficient palladium-catalyzed α-arylation of ketones and reach unprecedented turnover numbers for N-based ligands, offering a promising alternative to phosphine ligands.

## Graphical abstract

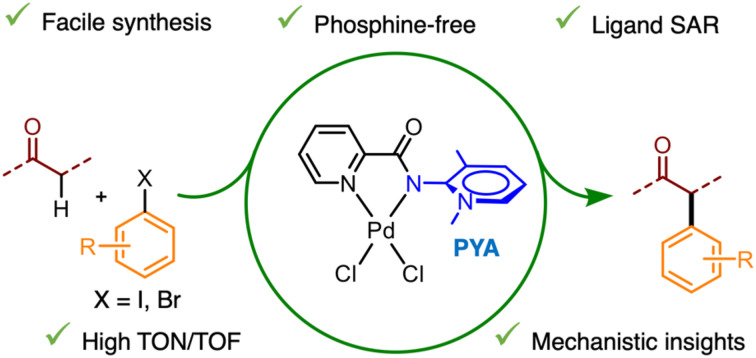

## Introduction

**Scheme 1:**
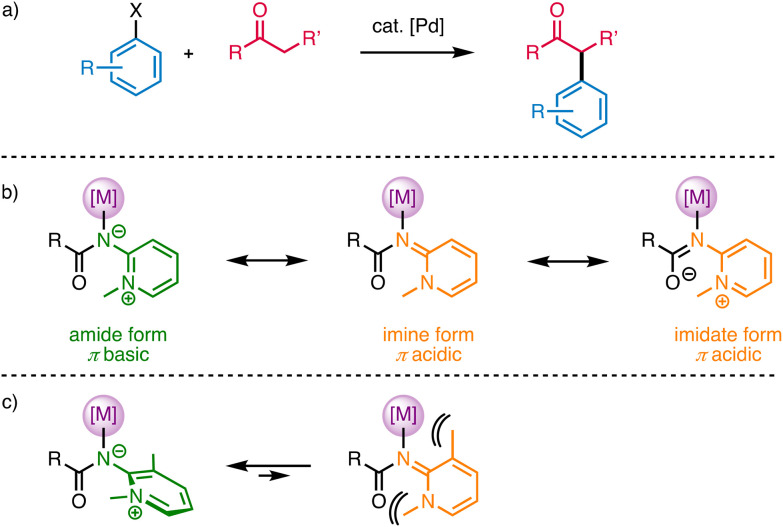
(a) Generic palladium-catalyzed α-arylation of ketones; (b) limiting resonance structures of 2-pyridylidene amide (PYA) ligands; (c) steric implications of *ortho*,*ortho*-disubstitution of 2-pyridylidene amide ligands.

Transition metal-catalysed cross-coupling methodologies have become a standard concept in the development of pharmaceuticals, natural product synthesis, and in the fine chemicals industry.^1–3^ In the late 1990s, Buchwald and Hartwig expanded the set of cross-coupling reactions to direct α-arylation of enolizable ketones (Scheme 1a),^4–7^ which provides versatile chemical intermediates and also a motif that is abundant in various active pharmaceutical ingredients (APIs).^8,9^ This process is considerably more sustainable than previously used stoichiometric methods, which often suffered from low scope and selectivity.^10–12^ Early studies indicated the suitability of palladium catalysts for ketone α-arylation, in particular when combined with sterically hindered strongly electron-donating phosphine ligands.^6,7^ Especially wide bite angle diphosphine ligands were noted to provide high steric hindrance, which suppresses undesired β-H elimination.^4,5,13^ N-Heterocyclic carbenes (NHCs) have emerged as valuable alternatives to phosphines for the α-arylation of ketones^14^ and provided access to molecularly well-defined pre-catalysts in contrast to the phosphine-based systems, which were typically generated *in situ*.^15–19^ Ligand tailoring enabled the activation of more challenging aryl chloride substrates, which was achieved in some instances even at room temperature and in aqueous media,^20–22^ and the substitution of palladium with Earth-abundant nickel, albeit with only modest turnover numbers (TONs).^23^ Moreover, the accessibility of molecularly defined pre-catalysts allowed for a better understanding of some mechanistic aspects such as the formation of the catalytically active palladium(0) species.^6,24,25^ NHC-based catalysts generally feature only moderate turnover frequencies (TOFs), which has been attributed to slow reductive product elimination imparted by the monodentate ligation of NHCs while chelating phosphines are supposed to accelerate this step considerably.^5,25–27^

Based on these ligand requirements, we became interested in exploring the suitability of pyridylidene amide (PYA; Scheme 1b) palladium complexes as catalyst precursors for this transformation. PYA ligands are synthetically easily accessible from cheap aminopyridine, they offer vast opportunities to introduce a chelating donor site, and they are amongst the strongest neutral (L-type) N-donor ligands known thus far.^28^ Early work indicated a donor strength of these ligands that is comparable to classic NHC ligands.^29,30^ Moreover they are defined by limiting resonance structures that feature either π-basic or π-acidic properties (Scheme 1b) and thus suggest electronic donor-flexibility that responds to the electronic situation of the coordinated metal center.^28^ Such flexibility may be particularly powerful in palladium-catalyzed cross-coupling processes, which entail both oxidative addition and reductive elimination steps and thus require the stabilization of palladium in both zero-valent and 2^+^ oxidation states. The accessibility of these states is expected to benefit from ligands that have the potential to flexibly adjust their donor properties. Moreover, introduction of a potentially hemilabile pyridine chelating site has been accomplished,^31,32^ thus offering also flexibility to toggle between mono- and bidentate coordination modes. Indeed, previous work indicated the suitability of such pyridyl-PYA ligands in palladium-catalyzed Suzuki–Miyaura cross coupling reactions,^31^ as well as other oxidation reactions.^33–37^

Here we demonstrate that pyridyl-PYA palladium complexes constitute precursors of catalysts for the α-arylation of ketones with excellent activity and high selectivity. Key for the high catalytic activity is the presence of two *ortho*-substituents in the PYA ligand, a concept that has also been applied for palladium-catalyzed olefin oligomerization.^38^ This substitution pattern increases the PYA σ-donor properties and also the steric hindrance with the amide group, which enhances the relevance of the zwitterionic resonance forms and hence the donor strength of the PYA ligand (Scheme 1c). The high activity of these complexes therefore expands the rather narrow range of ligands available for ketone α-arylation.

## Results and discussion

### Synthesis and characterization of Pd PYA complex

**Scheme 2:**
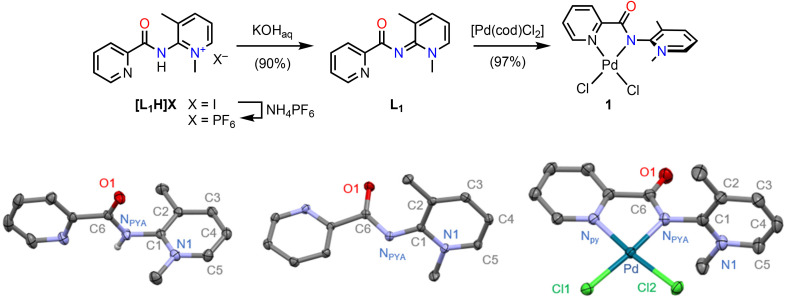
Synthesis of complex 1 and molecular structures of the [L_1_H]^+^ cation, the free ligand L_1_ and complex 1 (30% probability ellipsoids, all carbon-bound hydrogen atoms omitted for clarity). Selected bond lengths for complex 1: Pd–N_PYA_ 2.006(3) Å, Pd–N_py_ 2.013(3) Å, Pd–Cl1 2.3030(9) Å, Pd–Cl2 2.2935(11) Å.

Complex 1 was prepared from the previously reported PYA ligand [L_1_H]I in two straightforward high yielding steps (Scheme 2).^38^ Aqueous KOH was used to first deprotonate the iodide salt. The resulting neutral ligand L_1_ was then reacted with [Pd(cod)Cl_2_] to afford complex 1 in essentially quantitative yield as an orange solid that is stable towards air, light, and moisture for weeks. Completion of the reaction was easily monitored by ^1^H NMR spectroscopy as the PYA ligand N-CH_3_ singlet shifts from *δ*_H_ = 3.86 in L_1_ downfield to 4.34 ppm in complex 1. Additionally, the aromatic proton adjacent to the pyridine nitrogen is more deshielded (9.23 ppm in 1*vs.* 8.61 ppm in L_1_; CD_2_Cl_2_), indicative of pyridine coordination and hence chelation of the ligand. The significant deshielding was attributed to electrostatic interactions of this proton with the chloride ligand. Single-crystal X-ray diffraction analysis of complex 1 revealed the palladium center in its typical, slightly distorted square-planar geometry, defined by the two chloride ligands and the chelating pyridine-PYA ligand. The chelate bite angle is 80.35(10)°, which is in line with similar 5-membered palladacycles.^39^ The structure also features a close contact between H_pyr_ and the chloride *cis* to the pyridyl unit (H_pyr_⋯.Cl 2.6895(8) Å) as inferred from NMR spectroscopy. Interestingly, the Pd–N_PYA_ bond is slightly shorter than in a previously reported *para*-PYA-pyr Pd^II^ complex (2.006(3) *vs.* 2.0392(14) Å),^31^ yet similar to the Pd–N bond in [Pd(bipy)Cl_2_].^39^ Remarkably, the two Pd–Cl bonds are essentially equally long, even though one is *trans* to the PYA and the other *trans* to pyridine (2.303(9) *vs.* 2.2935(11) Å). Likewise, the two Pd–N bonds do not differ significantly.

To better understand the behavior of the PYA ligand, the solid state structures of the protonated ligand precursor [L_1_H]^+^ and its deprotonated form L_1_ were compared to the structure of complex 1 (Table 1). [L_1_H]^+^ can be considered as the limiting X-type zwitterionic resonance form of the PYA unit with the N_PYA_ electron pair fully engaged in covalent N–H bonding, while L_1_ represents the L-type quinoidal limiting resonance form with the N_PYA_ electron pair fully available for stabilizing the positive charge of the pyridinium unit. Diagnostic probes for these two limiting forms are, for example, the exocyclic C1–N_PYA_ bond, which has predominantly double bond character in quinoidal L_1_ and significant single bond character in [L_1_H]^+^ (1.333(1) Å *vs.* 1.401(4) Å). Moreover, the zwitterionic form in [L_1_H]^+^ results in an essentially orthogonal orientation of the PYA amide unit with respect to the pyridinium heterocycle as defined by the N1–C1–N_PYA_–C6 dihedral angle *θ* = 89(7)°. In the quinoidal form, the amide unit is much more in plane with the PYA heterocycle and *θ* reduces to 39.9(1)° in L_1_. Full coplanarity is probably prevented by the steric repulsion between the two *o*-methyl groups of the pyridinium and the carbonyl unit.^40,41^ In complex 1, these diagnostic metrics are highly similar to those of [L_1_H]^+^ (Table 1), indicating that in the solid state, the PYA ligand adopts a predominantly zwitterionic structure with X-type bonding to the palladium center. For comparison, previously reported *para*-PYA systems had significantly smaller torsion angle *θ* of 44.91(16)°,^31^ and also *ortho*-PYA systems without the second *ortho*-methyl group feature smaller dihedral angles than 1 with *θ* around 60°.^38,40^

**Table 1:** Selected solid-state X-ray and solution ^1^H NMR metrics for [L_1_H]^+^, L_1_ and complex 1 a

	[L_1_H]^+^ b	L_1_	1
C1–N_PYA_	1.401(4)	1.333(1)	1.395(4)
C2–C3	1.384(3)	1.370(2)	1.373(5)
C3–C4	1.368(5)	1.397(2)	1.369(5)
*θ* c	89(7)	39.9(1)	87.8(4)
*δ*_NCH_3__	4.32	3.86	4.34
*δ*_H(4)_	7.83	6.73	7.54

a Bond lengths in Å, bond angles in deg, chemical shifts in ppm (in CD_2_Cl_2_).

b Average value from 4 independent molecules in the asymmetric unit.

c Torsion angle between amide unit and PYA heterocycle defined by N1–C1–N_PYA_–C6, see also Fig. S38 and Table S5.†

Also in solution, the zwitterionic form of the PYA ligand prevails in complex 1 according to ^1^H NMR spectroscopic data. Specifically, all resonances for the heterocyclic PYA protons undergo a substantial upfield shift upon ligand deprotonation, for example, the signal for H(4) located *para* to the pyridinium nitrogen appears at *δ*_H_ = 6.73 compared to 7.83 in [L_1_H]^+^. Upon palladium coordination in complex 1, this resonance is again deshielded (*δ*_H_ = 7.54), pointing to a similar electronic configuration as in the zwitterionic system of [L_1_H]^+^. Similarly, the N-CH_3_ resonance of the PYA has been proposed as a reporter group for distinguishing quinoidal *vs.* zwitterionic contributions.^42^ According to these shifts, the PYA ligand in complex 1 is also closer to the zwitterionic protonated ligand [L_1_H]^+^ (*δ*_H_ = 4.34 and 4.32, respectively), and distinct from the quinoidal deprotonated ligand L_1_ (*δ*_H_ = 3.86). Hence, both solution and solid-state analysis suggest a zwitterionic bonding of the PYA ligand in the ground state of complex 1, which results in a formal palladate system with an electron-rich palladium center. Such a configuration may promote the release of chloride ligands from complex 1 and may also be advantageous for mediating oxidative addition reactions. Moreover, the electronic flexibility of the PYA ligand may facilitate through its quinoidal form the accessibility of palladium(0) intermediates that are critical for C–C bond formation catalysis.

### Catalytic α-arylation of ketones

The catalytic activity of complex 1 was probed in the α-arylation of ketones using propiophenone and bromobenzene as model substrates (Table S6†). Evaluation of the reaction conditions involved the variation of the base and the solvent (Tables S6 and S7†). These experiments indicated the need for strong and sufficiently soluble base. Thus, DBU, Cs_2_CO_3_, or K_3_PO_4_ gave essentially no conversion, while NaO*t*Bu in a small excess (1.1. eq.) afforded both high conversion and high yields (91%). Modification of the alkali metal to LiO*t*Bu or KO*t*Bu gave lower yields under otherwise identical conditions (72% and 59%, respectively, Table S6 and Fig. S39†). When the solvent was changed from dioxane to more polar media (*n*BuOH, EtOH, MeNO_2_, MeCN), conversions were high but little to no desired arylated ketone product was observed. In less polar solvents such as THF, 2-MeTHF or toluene^43^ yields were better (40–60%), though lower than in dioxane (91%, Table S7†). The inhibiting effect of THF rationalizes also the low yields observed when NaO*t*Bu was added as a THF solution. The decent performance of complex 1 in bio-based, inexpensive 2-methyl-THF (58% yield in 90 min) is promising when considering a green alternative for this reaction.^44^ Several control experiments were run to assess the role of 1 in the reaction (Table 2). Replacing 1 by its precursor PdCl_2_ resulted in a drop of activity from 91% to 20% yield under similar conditions (entry 2). Removal of the base or the catalyst completely inhibited the reaction as no detectable amount of product was observed (entries 3 and 4). Interestingly, when the reaction was run under reflux rather than in a closed vial, the yield dropped to 33%. The low overpressure built during the reaction seems to have a positive impact on the rate and selectivity of the reaction. Similar observations have been made for related cross-coupling reactions.^45,46^ The use of CH_2_Cl_2_ as co-solvent to improve the catalyst solubility had a detrimental effect as yields decreased from 91% to 19% when the catalyst was pre-dissolved in CH_2_Cl_2_ and to 31% when CH_2_Cl_2_ was added as a co-solvent (entries 6 and 7). The yield and selectivity were also reduced when the reaction was run in air rather than a N_2_ atmosphere (entry 8), presumably because the intermediate palladium(0) species is sensitive to oxidation.

**Table 2:** Ketone arylation with complex 1 and control experiments^a^

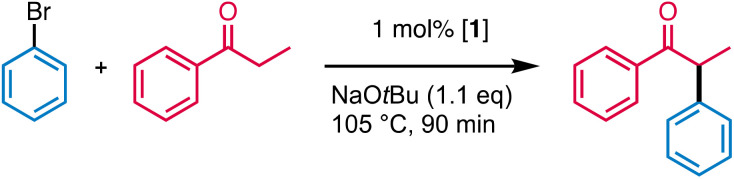			
Entry	Deviation from above	Yield^b^	Conversion
1	None	91%	94%
2	PdCl_2_ instead of 1	20%	30%
3	No catalyst	<1%	30%
4	No base	<1%	<1%
5	Under reflux	33%	68%
6	Catalyst pre-dissolved in CH_2_Cl_2_	19%	60%
7	0.5 ml CH_2_Cl_2_ added	35%	70%
8	Under air	27%	58%

a Reaction conditions: PhBr (1.0 mmol), Propiophenone (1.0 mmol), [Pd] (0.01 mmol), NaOtBu (1.1 mmol), dioxane (1.0 mL) in a 10 mL microwave vial, 105° for 90 min under N_2_.

b Yields determined by GC analysis using hexamethylbenzene as internal standard.

Further optimization of the catalytic performance included the concentration of the reagents (Table S8 and Fig. S40†). At concentrations lower than 0.5 M full conversion required more than 1 h (TOF = 220 h^−1^). Upon raising the concentration to 2 M, the TOF_max_ increased to 280 h^−1^. Even at 8 M concentrations, essentially neat conditions, a 85% yield was achieved. These latter conditions enable an excellent e-factor of the process by reducing the costs of solvent disposal or recycling.^47^ At this specific concentration, 250 μL solvent were sufficient to produce >350 mg product. However, catalysis monitoring is prevented by the fast precipitation of NaBr and, therefore, substrate concentrations in the 1–2 M range were preferred for further catalyst exploration. Notably, the yield of the arylated ketone was only marginally affected when a 0.2 eq. excess of either reagent was used (Table S9 and Fig. S41†), which may become useful when one of the coupling partners is particularly precious as typical in late-stage functionalisation.^48–51^

**Fig. 1:**
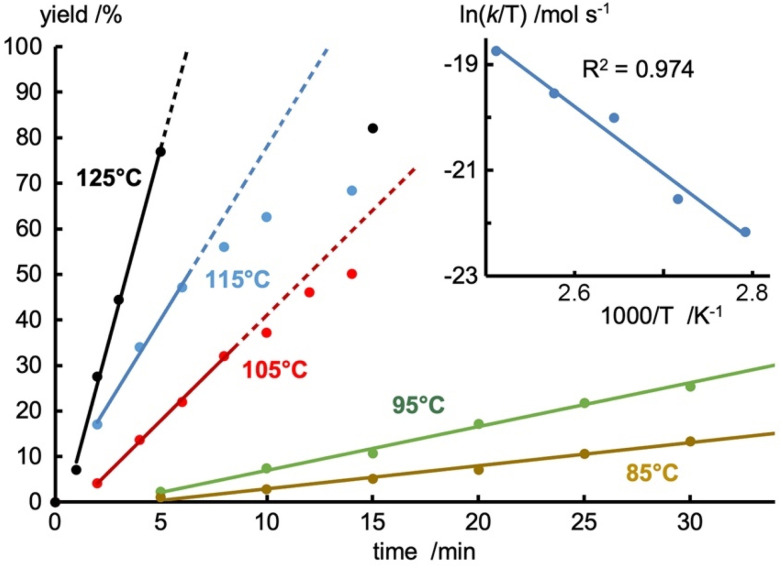
Time dependent conversion profiles for the coupling of propiophenone and bromobenzene catalyzed by 1 at different temperatures (dots) and initial rates (lines). Reaction conditions: ArBr (1.0 mmol), propiophenone (1.0 mmol), [1] (0.01 mmol), NaO*t*Bu (1.1 mmol), 1,4-dioxane (0.5 mL) in a 10 mL microwave vial. Spectroscopic yields measured by GC-FID with hexamethylbenzene as internal standard. Inset: eyring plot based on temperature-dependence of initial rates, yielding Δ*H*^‡^ = 105 ± 10 kJ mol^−1^ and Δ*S*^‡^ = −89 ± 26 J K^−1^ (Fig. S43 and Table S10†).

Variation of the reaction temperature revealed a high thermal robustness of the catalyst. At 115 °C, TOF_max_ increased to 450 h^−1^ and even to 1000 h^−1^ at 125 °C while keeping yields high (>85%, Table S10 and Fig. S42†). At this temperature, full conversion required less than 5 min. The catalyst is active also at lower temperature, and up to 70% yield was obtained at 85 °C, though full conversion was not reached even after 2.5 h. An Eyring plot of the initial rates at different temperatures provided the activation parameters Δ*H*^‡^ = 105 ± 10 kJ mol^−1^ and Δ*S*^‡^ = −89 ± 26 J K^−1^ mol^−1^ (Fig. 1). The negative activation entropy value is indicative of an associative rate determining step.^52–54^

### Catalyst variations

**Scheme 3:**
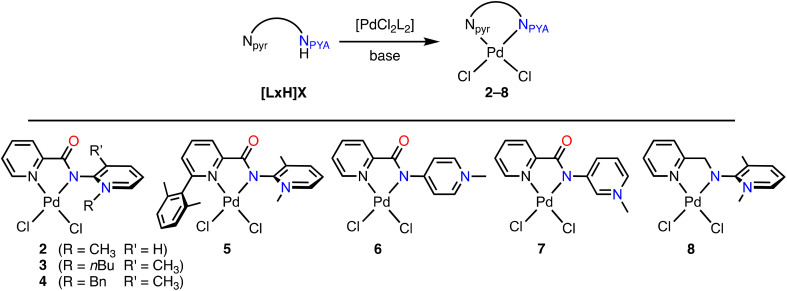
Synthetic pathway to palladium complexes 1–8. General conditions: aqueous KOH (2.0 M), then [PdCl_2_(cod)] in CH_2_Cl_2_; for complex 6: DBU (1.7 eq.), [PdCl_2_(PhCN)_2_] in CH_2_Cl_2_; for complex 7: (iii) Cs_2_CO_3_ (3 eq.), [PdCl_2_(cod)] in CH_2_Cl_2_; see ESI† for synthetic details. 2–8.

In an effort to improve the performance of the catalyst and to gain mechanistic insights, a series of palladium complexes 2–8 was designed (Scheme 3). These complexes all contain a *N*,*N*′-bidentate ligand related to that in 1. Specifically, complex 2 features a proton rather than a methyl substituent on the PYA *ortho* carbon, which reduces the bulkiness around the PYA and facilitates wagging or even rotation about the C1–N_PYA_ bond. In complexes 3 and 4, the methyl substituent of N1 was exchanged for a butyl and benzyl group, respectively, to probe the effect of steric bulk. Complex 5 contains a shielding xylyl-substitutent on the pyridyl side of the *N*,*N*′-chelate to introduce steric protection of the catalytic site. The 2,6-xylyl substitution was preferred over the phenyl counterpart to prevent cyclometallation. In complexes 6 and 7, the PYA system was changed from *ortho* to *para* and *meta*, respectively, to establish the electronic impact of the PYA system on catalysis. Finally, complex 8 contains a methylene rather than a carbonyl linker between the pyridyl and the pyridylidene site, thus constituting formally a pyridylidene amine (PYE) rather than a pyridylidene amide. This modification is expected to enhance the donor properties and the conformational flexibility, though it also features reactive C–H bonds.

Complexes 2–8 were prepared in a manner analogous to 1, either by sequential deprotonation of the precursor salt, isolation of the free ligand, and subsequent palladation (complexes 2–5 and 8), or by a single step procedure involving *in situ* palladation in the presence of a mild base such as DBU or Cs_2_CO_3_ (complexes 6 and 7; see ESI section 1† for synthetic details).^31^ All complexes were obtained as air- and moisture-stable red to orange complexes in good yields (>70%). Generally, the reaction was followed by ^1^H NMR spectroscopy, since the signal of the PYA *N*-alkyl α-protons shift significantly upon complexation. For instance in 2, the diagnostic N-CH_3_ singlet shifted downfield from *δ*_H_ = 3.87 in L_2_ to 4.24 in complex 2. In complexes 3 and 4 containing *N*-butyl and *N*-benzyl substituents, respectively, the α-CH_2_ protons become diastereotopic upon palladation, and feature characteristic vicinal coupling constants ^2^*J*_HH_ = 13.4 and 14.8 Hz, respectively (Fig. S27 and S29†). Distinct to the *para* and *ortho*-PYA complexes, the N-CH_3_ resonance in the *meta*-PYA complex 7 shifted upfield upon palladation from *δ*_H_ = 4.45 to 4.24. Complexation of the PYE ligand yielded complex 8 featuring a five-membered palladacycle with fluxional conformation.^55^ At 213 K, the bridging methylene protons appear as two well-resolved doublets at *δ*_H_ = 4.15 and 5.65 (^2^*J*_HH_ = 15.6 Hz), while at room temperature, coalescence is reached, and the signals are not detectable. Warming the solution to 328 K, approaches the fast exchange limit with the CH_2_ group appearing as a broad singlet at 5.00 ppm (Fig. S36†).

**Fig. 2:**
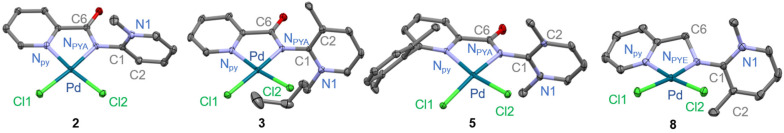
Molecular structures of complexes 2, 3, 5, and 8 determined by X-ray diffraction analysis (30% probability ellipsoids, hydrogen atoms and co-crystallized solvent molecules omitted for clarity).

Crystals suitable for X-ray diffraction were obtained for complexes 2, 3, 5 and 8 (Fig. 2). All complexes display the palladium center in a distorted square planar geometry with similar metrics as observed for complex 1 (Table 3). While PYA complexes 2, 3, and 5 feature a long exocyclic C1–N_PYA_ bond of at least 1.39 Å, indicative of a single bond and thus a predominantly zwitterionic electronic configuration in the solid state, their dihedral angles *θ* between the pyridylidene and the amide unit N1–C1–N_PYA_–C6 differs considerably. The angle is close to orthogonal in complex 2 (89.4(3)°) and similar to complex 1, yet only around 70° in complexes 3 and 5 (69.5(11)° and 71.55(17)°, respectively), suggesting some wagging about the C1–N_PYA_ bond. Also in complex 8, the angle *θ* is 71.9(3)°, though in this complex, also the C1–N_PYE_ is shorter, 1.341(4) Å, suggesting some imine character and hence a larger contribution of the neutral quinoidal PYE resonance form than in the crystallographically characterized PYA complexes 1–3 and 5. Despite these notable modulations in the PYA ligand backbone and the distinct donor properties of the PYA ligand compared to pyridine, the metrics around the palladium center are essentially identical in all complexes 1–3, 5 and 8. All Pd–N bond distances are around 2.02(1) Å, irrespective of the N-donor, and likewise, no difference in *trans* influence is noticeable with all Pd–Cl bond lengths around 2.30(1) Å. Only complex 5 features a slightly elongated Pd–N_py_ bond (2.0719(13) Å) and a compression of the *cis*-located Pd–Cl bond (2.2745(7) Å), which presumably originates from the steric impact of the xylyl substituent in this complex.

**Table 3:** Selected metrics for the molecular structures of complexes 1–3, 5, and 8

	1	2	3 a	5	8 b
C1–N_PYA_/Å	1.395(4)	1.390(3)	1.397(23)	1.392(2)	1.341(4)
N_PYA_–C6/Å	1.340(4)	1.346(4)	1.355(13)	1.345(2)	1.472(5)
Pd–N_py_/Å	2.013(3)	2.024(2)	2.034(9)	2.0719(13)	2.021(3)
Pd–N_PYA_/Å	2.006(3)	2.014(2)	2.010(8)	2.0140(15)	2.015(2)
Pd–Cl1/Å	2.3030(9)	2.3153(9)	2.299(6)	2.2745(7)	2.3185(7)
Pd–Cl2/Å	2.2935(11)	2.2906(9)	2.291(3)	2.2963(6)	2.2941(9)
*θ* c	87.8(4)	89.4(3)	69.5(11)	71.55(17)	71.9(3)
%*V*_bur _d	40.2	38.5	43.3	48.3	41.6
*τ*_4_ e	0.084	0.086	0.093	0.162	0.105

a Average value from 2 independent molecules in the asymmetric unit.

b N_PYA_ should read N_PYE_.

c Torsion angle *θ* between amide unit and PYA heterocycle defined by N1–C1–N_PYA_–C6.

d Percentage buried volume %*V*_bur_ determined according to ref. 56, see ESI† for details.

e Tetrahedral distortion parameter *τ*_4_ determined according to ref. 59.

The percentage buried volume %*V*_bur_ ^56,57^ was used to quantify the steric influence of the different ligands and to estimate the accessibility of the metal center in each of the crystallized complexes (see also ESI†). As expected from the ligand design, the mono-substituted *o*-PYA ligand in complex 2 is the smallest in the series with 38.5%*V*_bur_, followed by the *o*,*o*-disubstituted PYA in complex 1 with 40.2%*V*_bur_. The PYE analogue in complex 8 is sterically slightly more demanding, 41.6%*V*_bur_, while substitution of the *N*-methyl group with *n*Bu further enhances the steric congestion around palladium with a 43.3%*V*_bur_ for 3. Introducing a xylyl substituent on the pyridine resulted in the largest steric shielding with 48.3%*V*_bur_ for complex 5. These steric parameters are not too dissimilar from those of benchmark cross-couplings catalyst precursors with bidentate phosphine ligands, *cf.* 47.0%*V*_bur_ for [PdCl_2_(dppm)] or 55.5%*V*_bur_ for [PdCl_2_(dppf)].^58^ We also note that the substantial shielding of L_5_ induced a significant distortion of the square planar configuration in complex 5, indicated by a high *τ*_4_ value (0.162) in comparison with the other complexes (*τ*_4_ around 0.09).^59^

Evaluation of complexes 2–8 as catalyst precursors in the α-arylation of propiophenone under standard conditions revealed a distinct influence of the ligand on the catalytic activity (Table 4 and Fig. S44†). Thus, removing the *o*-methyl group of the PYA ligand reduced the TOF_max_ from 220 h^−1^ for 1 to 115 h^−1^ for complex 2, and full conversion of the starting materials was not achieved (entries 1 and 2). After about 45 min and 72% yield, the catalytic activity of complex 2 essentially ceased. Similarly, the addition of bulkier *N*-substituents on the PYA had a negative effect on the catalysis since both 3 and 4 displayed much lower catalytic activity (TOF_max_ 70 h^−1^) than 1, suggesting that steric demand on the PYA side is hindering turnover (entries 3 and 4). In contrast, introduction of steric bulk on the pyridyl side essentially doubled the activity with TOF_max_ = 470 h^−1^ for complex 5 and essentially complete conversion after 15 min, albeit at the cost of a reduced selectivity since the yield dropped to 82% (entry 5). When comparing the different PYA isomers 2, 6, and 7, the *meta* PYA variant showed higher activity than 2 with TOF_max_ = 245 h^−1^ (85% yield), while the *para* PYA system performed similar to 2 (TOF_max_ = 135 h^−1^, 71% yield; entries 6 and 7). This catalytic activity correlates well with the donor properties of the different PYA isomers.^29,36^ The removal of the carbonyl moiety in 8 was detrimental both in terms of reaction rate (140 h^−1^; entry 8) and especially selectivity, as only a modest 37% yield was obtained. The pertinent time-conversion profile with complex 8 revealed a sharp drop of activity after 5 min, indicating that the benzylic methylene linker is unstable under catalytic conditions. In summary, addition of steric hindrance on the pyridyl side afforded the most active catalyst of the series, though highest yields and selectivity were achieved with complex 1.

**Table 4:** Effect of the different ligands on the efficiency of ketone arylation catalysis^a^

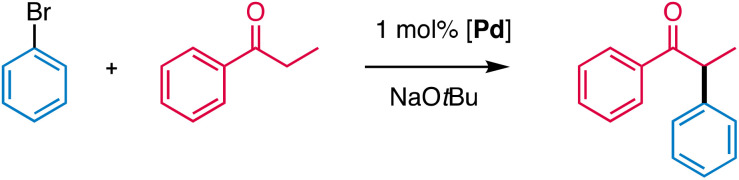					
Entry	Complex	Yield	Ketone conversion	PhBr conversion	TOF_max_/h^−1^
1	1	91%	94%	>99%	220
2	2	72%	91%	92%	115
3	3	68%	74%	75%	70
4	4	64%	82%	81%	70
5	5	82%	94%	>99%	470
6	6	71%	91%	92%	135
7	7	85%	95%	98%	245
8	8	37%	69%	65%	140
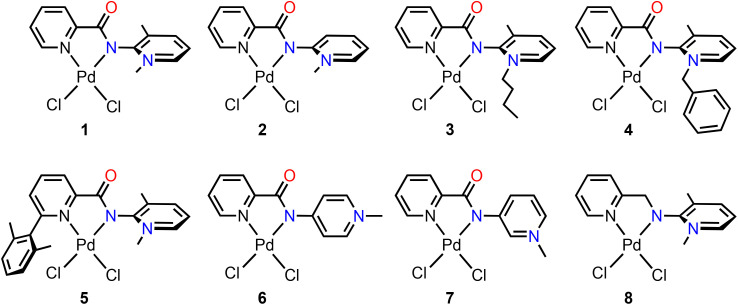					

a Reaction conditions: ArBr (1.0 mmol), propiophenone (1.0 mmol), [Pd] (0.01 mmol), NaO*t*Bu (1.1 mmol), 1,4-dioxane (1.0 mL) in a 10 mL microwave vial, 90 min, 105 °C, N_2_.

### Catalyst scope and limitations

The maximum performance of these catalysts was explored by raising the temperature to 125 °C. Under these conditions, complex 5 reached TOF_max_ as high as 2700 h^−1^ with full conversion within 5 min and an acceptable yield of 80%, as side reactions such as aldol condensations also increased (Table 5, entry 1 and Fig. S46†). Such very short reaction times might be of interest for time-sensitive substrate such as, for instance, short-lived ^18^F-labelled compounds.^60,61^ Under the same high temperature conditions, complex 1 was again slower, yet yielded the arylated ketone in 87% yield (entry 2), thus indicating a high thermal robustness of these catalytic systems while maintaining selectivity. Upon lowering the catalyst loading from 1 mol% to just 0.04 mol%, yields dropped slightly to 76%, equivalent to 1900 TON, and the TOF_max_ reached 3900 h^−1^ (entry 3). Even higher performance was accomplished when running an experiment with 0.01 mol% catalyst loading (100 ppm), reaching 7300 turnovers and a TOF_max_ = 8900 h^−1^, while keeping the yield at 73% (entry 4, Fig. S45†). The relevance of these low-catalyst loadings^62^ was demonstrated by running a gram-scale experiment with 0.01 mol% loading, which afforded 1.82 g 1,2-diphenylpropan-1-one in 70% isolated yield after 4 h reaction time. These TON and TOF values are unprecedented for catalysts with N-donor ligands. In comparison, the system reported by Connell based on a NCN pincer ligand achieved 860 TON.^63^ Although NHC based catalysts achieved notable activity in aryl chloride conversion, TONs do not exceed 1000 even when using aryl bromides for the arylation of ketones.^26^ However, higher TONs (74 000) were observed after extended reaction times (24 h *vs.* 2 h here) by Buchwald and co-workers in a ligand-free process.^4^

**Table 5:** Optimization of catalyst performance^a^

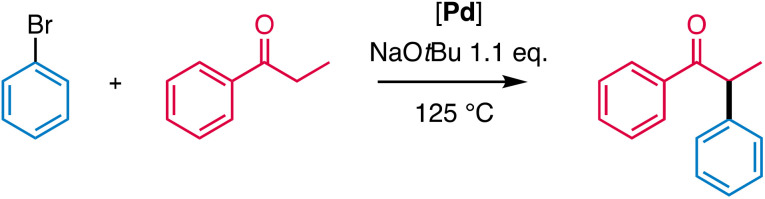						
Entry	[Pd]	Catalyst loading	Yield	Conversion	TON	TOF_max_ (h^−1^)
1	5	1 mol%	80%	98%	80	2700
2	1	1 mol%	87%	94%	87	1200
3	1	0.04 mol%	76%	95%	1900	3900
4	1	0.01 mol%	73% (70%)^b^	99%	7300	8900

a Reaction conditions: for entries 1,2: PhBr and propiophenone (1.0 mmol each), [Pd] (0.01 mmol), NaOtBu (1.1 mmol), 1,4-dioxane (0.5 mL) in a 10 mL vial, 90 min, 125 °C; entry 3: PhBr and propiophenone (3.09 mmol each), [Pd] (1.24 μmol), NaOtBu (3.40 mmol), 1,4-dioxane (1.55 mL) in a 10 mL vial, 120 min, 125 °C; entry 4: PhBr and propiophenone (12.4 mmol each), [Pd] (1.24 μmol), NaOtBu (13.6 mmol), 1,4-dioxane (6.2 mL) in a 25 mL vial, 240 min, 125 °C.

b Isolated yield in parentheses.

**Scheme 4:**
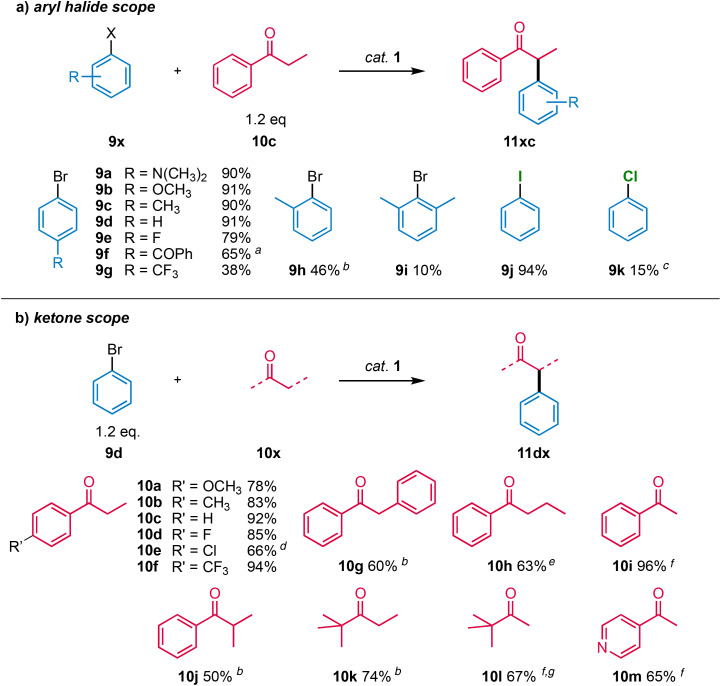
Substrate scope for catalyst 1. General reaction conditions for aryl halide scope: aryl halide 9x (1.0 mmol), ketone 10c (1.2 mmol), Pd complex 1 (0.01 mmol, 1 mol%), NaO*t*Bu (1.3 mmol), 1,4-dioxane (0.5 mL), 2 h, 105 °C; general reaction conditions for ketone scope: phenyl bromide 9d (1.2 mmol), ketone 10x (1.0 mmol), Pd complex 1 (0.01 mmol, 1 mol%), NaOtBu (1.1 mmol), 1,4-dioxane (0.5 mL), 2 h, 105 °C; ^*a*^ 1.0 mL 1,4-dioxane; ^*b*^ after 24 h; ^*c*^ at 125 °C; ^*d*^ Ar–Cl bond activated side products formed (Fig. S65†); ^*e*^ ketone concentration reduced to 0.5 M; ^*f*^ increased quantities of ketone (1.5 mmol) and NaOtBu (3 mmol); ^*g*^ di-arylated side product formed (3% yield).

Since complex 1 afforded the highest yields, this complex was selected to investigate the substrate scope of ketone α-arylation. Variation of the aryl halide included electron-rich aryl bromides (9a–c), which were efficiently coupled to propiophenone (Scheme 4a). In contrast, electron-withdrawing substituents (9e–9g) reduced the yield, which was particularly obvious with the electron-deficient CF_3_-substituted aryl bromide 9g, which gave only a low 38% yield did not improve by extending reaction time beyond 2 h. Likewise, sterically demanding *ortho*-substituted aryl-bromides 9h and 9i were poorly converted and generally required longer reaction times to reach full conversion. 9-Bromoanthracenyl did not react at all. Iodobenzene 9j was converted in excellent 94% yield, while chlorobenzene 9k gave only 15% yield, even when using higher catalyst loading (5 mol%) and elevated reaction temperatures (125 °C; Table S11†). Notably, the initial activity is appreciable (Fig. S50†), suggesting that in principle, the catalytic system has potential to convert aryl chlorides.

Variation of the ketones included electron-rich and -poor propiophenones (10a–f; Scheme 4b). In general, yields were slightly reduced upon including substituents, except for 10e where C–Cl bond activation was competitive and generated considerable amounts of side products (Fig. S64 and S65†). Substitution of the ketone α position with a bulkier substituent resulted in reduced yields, though both aromatic (10g) as well as aliphatic substituents (10h) were tolerated. When acetophenone 10i was used as substrate, the monoarylated product 10g was obtained in a high 96% yield, when an excess of ketone was used. Interestingly, the tertiary ketone 10j was also arylated, albeit in a reduced yield (50%). Moreover, aliphatic ketones (10k, 10l) were arylated in decent yields. Likewise, the heteroaromatic 4-acyl-pyridine 10m was converted well despite the potential coordination ability of both the substrate and product.

### Mechanistic aspects

**Fig. 3:**
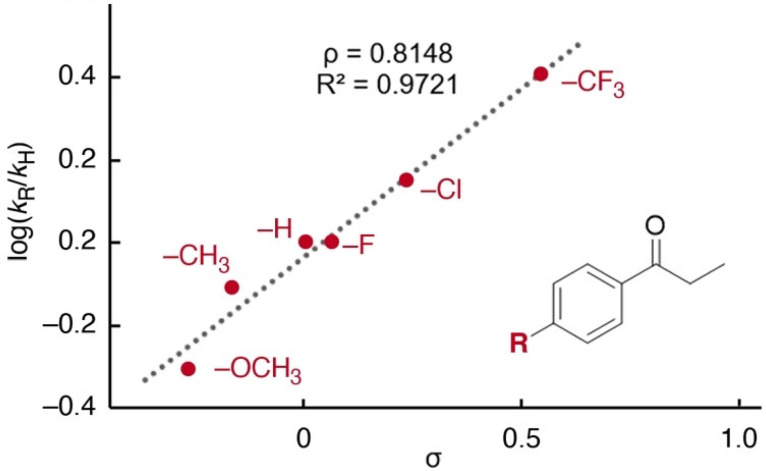
Hammett plot for the arylation of 4-substituted propiophenones (reaction conditions as in Scheme 4b).

Temperature-dependent analysis of the reaction rate and specifically the negative entropy of activation Δ*S*^‡^ = −89 ± 26 J K^−1^ mol^−1^ (*cf.*Fig. 1) suggests an associative turnover limiting step. This conclusion is in agreement with the fact that reactions in a sealed and pressurized vial proceed much faster than under reflux at 1 atm (*cf.*Table 2, entry 1 *vs.* 5). Moreover, analysis of the initial rates for the conversion of *para*-substituted aryl bromides 9a–g did not reveal a correlation with the Hammett parameter *σ*_p_ (*R*^2^ < 0.7, Fig. S49†), though a positive linear correlation was observed for *para*-substituted aryl ketones (*R*^2^ > 0.97, Fig. 3 and Fig. S47†),^64^ indicating the involvement of the ketone in the turnover limiting step. The highly positive slope, *ρ* = 0.81, points towards the build-up of almost a full negative charge in the transition state. These considerations together with the negative entropy are consistent with turnover-limiting enolate coordination to palladium. Notably, several studies demonstrated the relevance of large ligand bite angles to impart efficient reductive elimination rates.^65,66^ With the pyridyl-PYA ligands developed here, the turnover-limiting step is shifted to a redox-neutral ligand substitution process. Moreover, this work demonstrates that also chelating ligands with rather acute bite angles are efficient in α-arylation catalysis. Both these effects may be a direct consequence of the PYA donor-flexibility and promotion of both oxidative addition and reductive eliminations at the palladium center through the variable electron-donor properties.

## Conclusions

Here we demonstrated the straightforward accessibility to a series of air- and moisture-stable palladium(ii) complexes with a *N*,*N*′-bidentate chelating ligand based on electronically flexible PYA ligands. These complexes are active in the α-arylation of ketones and tolerate a range of functional groups both in the aryl bromide as well as the ketone substrates. They show activity that is unprecedentedly high for nitrogen-based ligands, reaching up to 7300 turnover numbers and close to 9000 h^−1^ TOF. These values are competitive with those of the best NHC or phosphine-based systems, indicating that PYAs offer a valuable and low-price alternative to those ligands. Ligand tailoring revealed that activity is enhanced with increased PYA donor properties and larger steric shielding, while selectivity improved when the pyridyl side is unsubstituted. These observations suggest that further tailoring of the imine donor, *i.e.* replacement of the pyridyl unit, may be prolific for further enhancement of catalytic activity. Overall, these ligand tailoring effects lend strong support to a catalytically active species that preserves coordination of the pyridyl-PYA ligand. Mechanistic considerations indicate that both oxidative addition and reductive elimination are swift with these PYA-based catalysts, presumably facilitated by the donor-flexibility of the PYA ligand, while enolate coordination is turnover-limiting. These insights provide opportunities for the development of other catalysts for challenging C–C bond formation methodologies.

## Conflicts of interest

The authors declare no competing financial interest.

## Supplementary Material

DT-052-D3DT03182A-s001

DT-052-D3DT03182A-s002
