# Supplementary material for: Electronically flexible PYA ligands for efficient palladium-catalyzed α-arylation of ketones
Source: Dalton Trans. 2023 Oct 18;52(45):16688–97. doi: 10.1039/d3dt03182a (PMC10660196; doi:10.1039/d3dt03182a)
Supplement: DT-052-D3DT03182A-s001 [file DT-052-D3DT03182A-s001.pdf]

Electronic Supplementary Material (ESI) for Dalton Transactions.
This journal is © The Royal Society of Chemistry 2023

Electronic Supporting Information

belonging to

Electronically flexible PYA ligands for efficient palladium-catalyzed α-arylation of ketones

Esaïe Reusser[a] and Martin Albrecht*[a]

[a] Department of Chemistry, Biochemistry and Pharmaceutical Sciences, University of Bern, Freiestrasse 3, 3012 Bern, Switzerland

Email: martin.albrecht@unibe.ch

Table of contents

S.1. SYNTHETIC PROCEDURES **S3**

S.1.1 General information S3

S.1.2 Synthesis of the pyridyl-pyridinium salt [L_5H]I S4

S.1.3 General procedure for the preparation of neutral pyridylidene amides L_1-L_5, L_8 .... S11

S.1.3.1 Synthesis of neutral PYA ligand L1 .... S11

S.1.3.2 Synthesis of neutral PYA ligand L_2 .... S13

S.1.3.3 Synthesis of neutral PYA ligand L_3 .... S14

S.1.3.4 Synthesis of neutral PYA ligand L_4 .... S16

S.1.3.5 Synthesis of neutral PYA ligand L_5 .... S17

S.1.3.6 Synthesis of neutral ligand L_8 .... S19

S.1.4 General procedure for the preparation of PYA palladium complexes 1-8 .... S22

S.1.4.1 Synthesis of pyridyl-PYA Pd complex 1 .... S22

S.1.4.2 Synthesis of pyridyl-PYA Pd complex 2 .... S24

S.1.4.3 Synthesis of pyridyl-PYA Pd complex 3 .... S25

S.1.4.4 Synthesis of pyridyl-PYA Pd complex 4 .... S27

S.1.4.5 Synthesis of pyridyl-PYA Pd complex 5 .... S28

S.1.4.6 Synthesis of pyridyl-PYA Pd complex 7 .... S30

S.1.4.8 Synthesis of pyridyl-PYA Pd complex 8 .... S32

S.2. CRYSTAL STRUCTURE DETERMINATIONS **S34**

S.3. BURIED VOLUME CALCULATIONS **S39**

S.4. CATALYTIC A-ARYLATION OF KETONES **S44**

S.5. REFERENCES **S66**

S.1. Synthetic procedures

S.1.1 General information

Salts **[L_1H]I**, [L_2H]**I**, **[L_6H]**I **[L_7H]I** and the precursor of **L_3** and **L_4**, N-(3-methylpyridin-2-yl)picolinamide, as well as complex **6** were prepared according to previously reported procedures.[S1,S2] Acetonitrile and dichloromethane were dried by passage through a solvent purification column. All other reagents and solvents were commercially available and used as received. Unless otherwise stated all the reactions were run under exclusion of air using standard Schlenk techniques under an atmosphere of dry nitrogen. Benzotriazol-1-yloxytripyrrolidinophosphonium hexafluorophosphate (PyBOP) and all other chemicals were purchased from commercial vendors and used as received.

Unless otherwise specified, NMR spectra were recorded at 25 °C on Bruker spectrometers operating at 300 or 400 MHz (^{1}H NMR) and 75 or 100 MHz (^{13}C{^{1}H} NMR), respectively. Chemical shifts (δ) are expressed in ppm downfield from $SiMe_4$ using the residual protonated solvent as an internal standard and coupling constants in Hz. Assignments are based on homo- and heteronuclear shift correlation spectroscopy.

High resolution mass spectrometry was carried out with a Thermo Scientific LTQ Orbitrap XL (ESI-TOF) or with a Thermo Scientific Q Exactive GC either directly used with direct injection probe (DIP) or coupled to a Trace 1310 Gas Chromatograph when electron impact ionization (EI-MS) was required to obtain satisfactory data. All the MS data were collected by the mass spectrometry group in the DCBP. Elemental analyses were performed on a Thermo Scientific Flash 2000 CHNS-O elemental analyzer by the microanalytic laboratory at the DCBP. GC-FID reaction follow-up was achieved using an Agilent technologies 7820A GC System. GC-MS reaction follow-up was carried out on a Perkin Elmer Clarus 500 GC coupled to Perkin Elmer Clarus 560S ESI mass spectrometer.

S.1.2 Synthesis of the pyridyl-pyridinium salt [L_5H]I

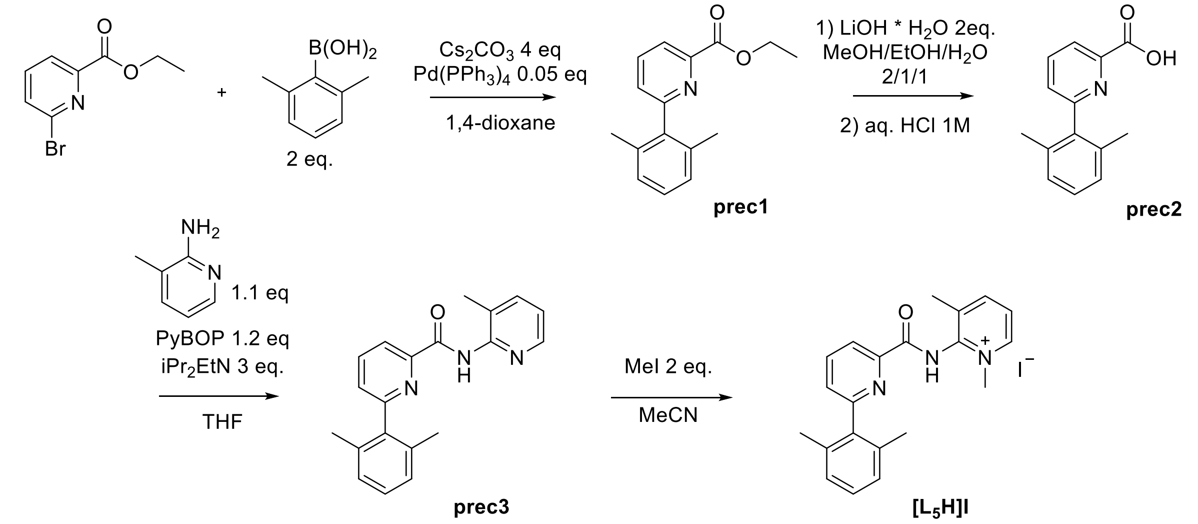

Scheme S1. Synthetic pathway to ligand precursor **[L_5H]I**

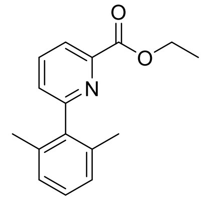

prec1. To a mixture of ethyl 6-bromopicolinate (200 mg, 0.87 mmol), (2,6-dimethylphenyl)boronic acid (261 mg, 1.74 mmol), Cs_2CO_3 (1.13 g, 3.48 mmol) and $Pd(PPh_3)_4$ (50.2 mg, 43.5 µmol) was added dry 1,4-dioxane (10 mL) and the resulting mixture was stirred for 16 h at 150 °C in a pressure tube. The resulting mixture was concentrated and purified by gradient flash chromatography (0 to 15% EtOAc in n-hexane; SiO_2) to yield the pure title compound as a white solid (124 mg, 62%). ^{1}H NMR (300 MHz, DMSO-d_6, 298 K): δ = 8.14 – 8.00 (m, 2H, CH_{pyr}), 7.58 (dd, J_{HH} = 7.1, 1.7 Hz, 1H, CH_{pyr}), 7.24 (t, $^3J_{HH}$ = 7.5 Hz, 1H, CH_{xyl}), 7.14 (d, $^3J_{HH}$ = 7.5, 2H, CH_{xyl}, 4.35 (q, $^3J_{HH}$ = 7.1, 2H, CH_2), 1.95 (s, 6H, xyl-CH_3), 1.32 (t, $^3J_{HH}$ = 7.1, 3H, OCH_2CH_3) ppm. ^{13}C{^{1}H} NMR (75 MHz, DMSO-d_6, 298 K): δ = 165.29 (*C*O), 159.58 (C_{pyr}-xyl), 148.16 (C_{pyr}-CO), 140.11 (C_{xyl}-pyr), 138.50 (*C*H_{pyr}), 135.66 (2C, $C_{xyl}CH_3$), 128.48 (2C, *C*H_{xyl} + *C*H_{pyr}), 127.94 (2C, *C*H_{xyl}) 123.56 (*C*H_{pyr}), 61.71 (*C*H_2), 20.37 (2C, xyl-*C*H_3), 14.62 (CH_2CH_3) ppm. HR-MS (m/z): calculated for $C_{16}H_{18}NO_2$ $[M+H]^+$ = 256.1338; found: 256.1337.

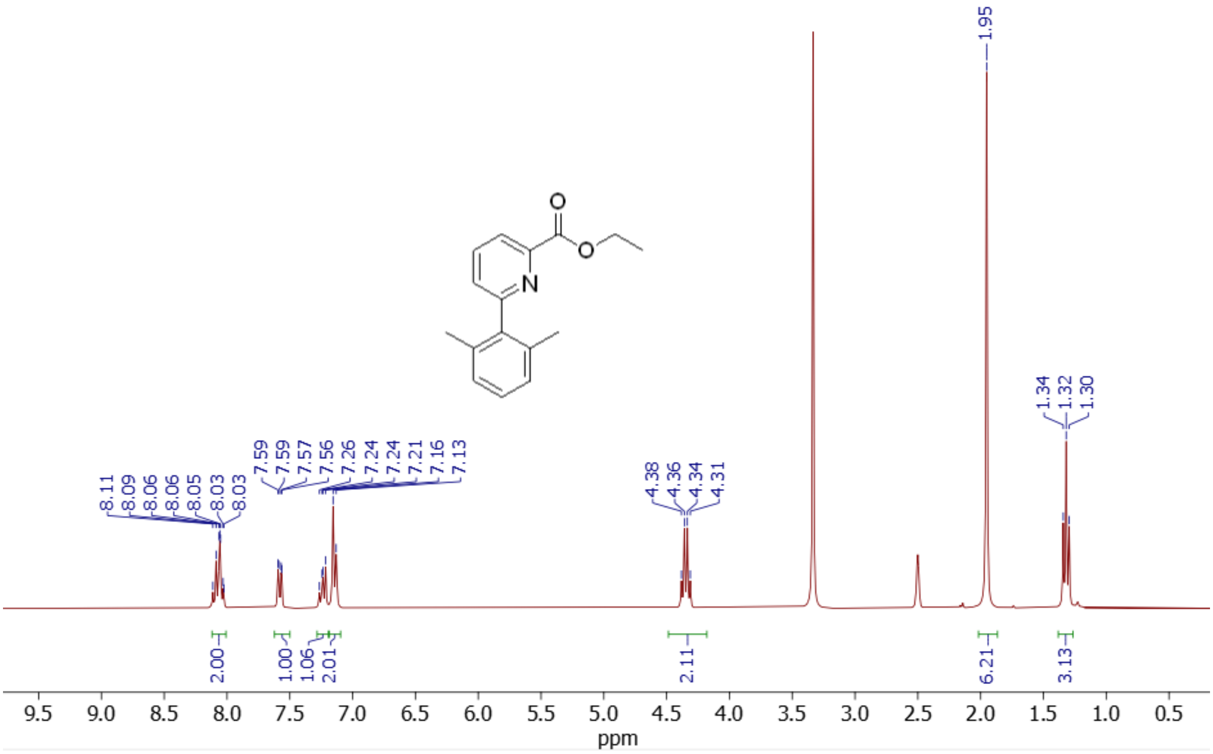

Figure S1. ^{1}H NMR spectrum (DMSO-d_6, 298 K, 300 MHz) of **prec1**.

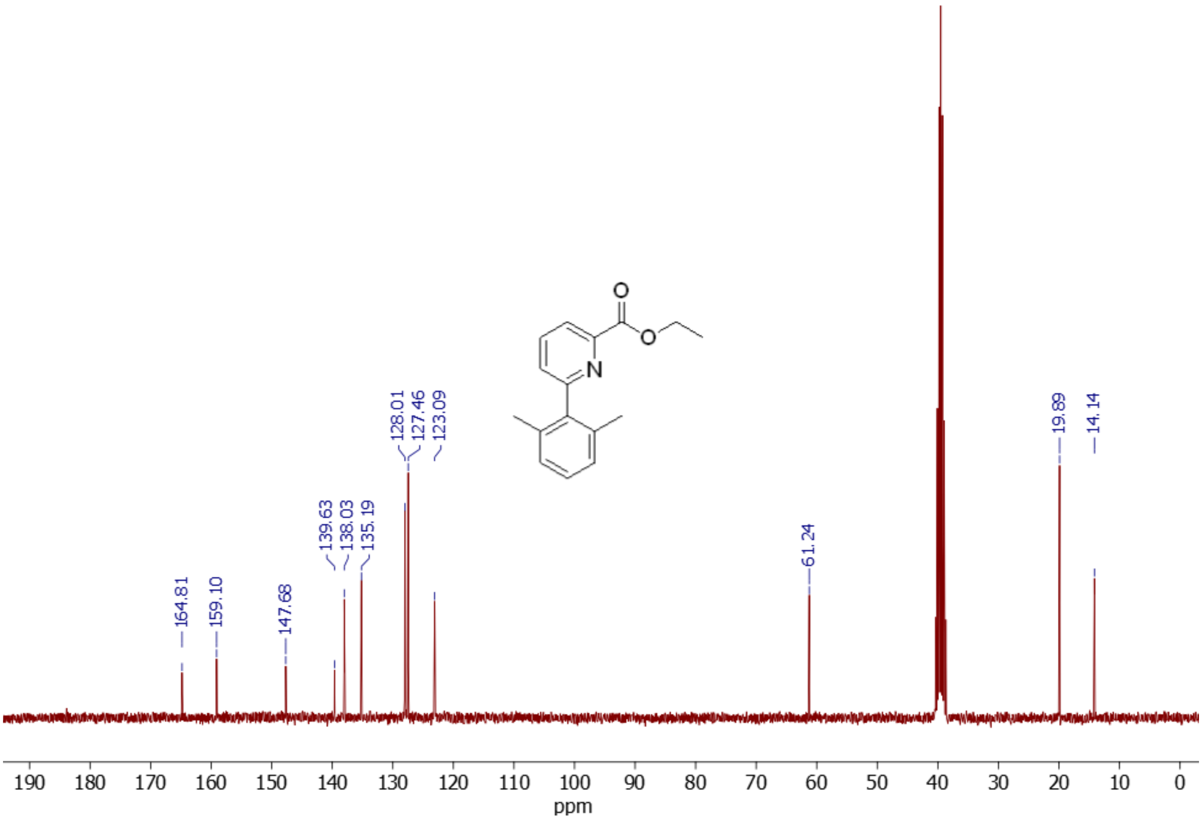

Figure S2. ^{13}C{^{1}H} NMR spectrum (DMSO-d_6, 298 K, 75 MHz) of **prec1**.

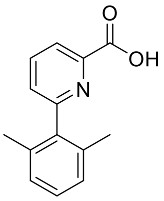

prec2. To a solution of ethyl 6-(2,6-dimethylphenyl)picolinate (200 mg, 0.78 mmol) in MeOH (8 mL), EtOH (4 mL) and H_2O (4 mL) was added lithium hydroxide hydrate (66 mg, 1.56 mmol) and the resulting mixture was stirred for 1 h at 23°C. Conversion was checked by TLC and, aq. HCl (1M, 2.4 mL, 2.4 mmol) was directly added. The reaction mixture was stirred for 10 min at the same temperature and then extracted with CH_2Cl_2 (3 x 20 mL). The combined organic layers were dried over $MgSO_4$ and concentrated under vacuum to yield the title compound as an off-white solid (178 mg, quant. yield). ^{1}H NMR (300 MHz, DMSO-d_6, 298 K): δ = 8.13 – 7.99 (m, 2H, CH_{pyr}), 7.56 (dd, J_{HH} = 7.1, 1.7 Hz, 1H, CH_{pyr}), 7.23 (t, $^3J_{HH}$ = 7.5 Hz, 1H, CH_{xyl}), 7.14 (d, $^3J_{HH}$ = 7.5, 2H, CH_{xyl}), 1.96 (s, 6H, xyl-CH_3) ppm. ^{13}C{^{1}H} NMR (75 MHz, DMSO-d_6, 298 K): δ = 166.27 (*C*O), 158.85 (C_{pyr}-xyl), 148.30 (C_{pyr}-CO), 139.70 (C_{xyl}-pyr), 137.98 (*C*H_{pyr}), 135.23 (2C, $C_{xyl}$$CH_3$), 127.96 (*C*$H_{xyl}$), 127.75 (*C*$H_{pyr}$) 127.43 (2C, *C*$H_{xyl}$), 122.91 (*C*$H_{pyr}$), 19.90 (2C, xyl-*C*$H_3$) ppm. HR-MS (m/z): calculated for $C_{14}H_{14}NO_2$ $[M+H]^+$ = 228.1025; found: 228.1025.

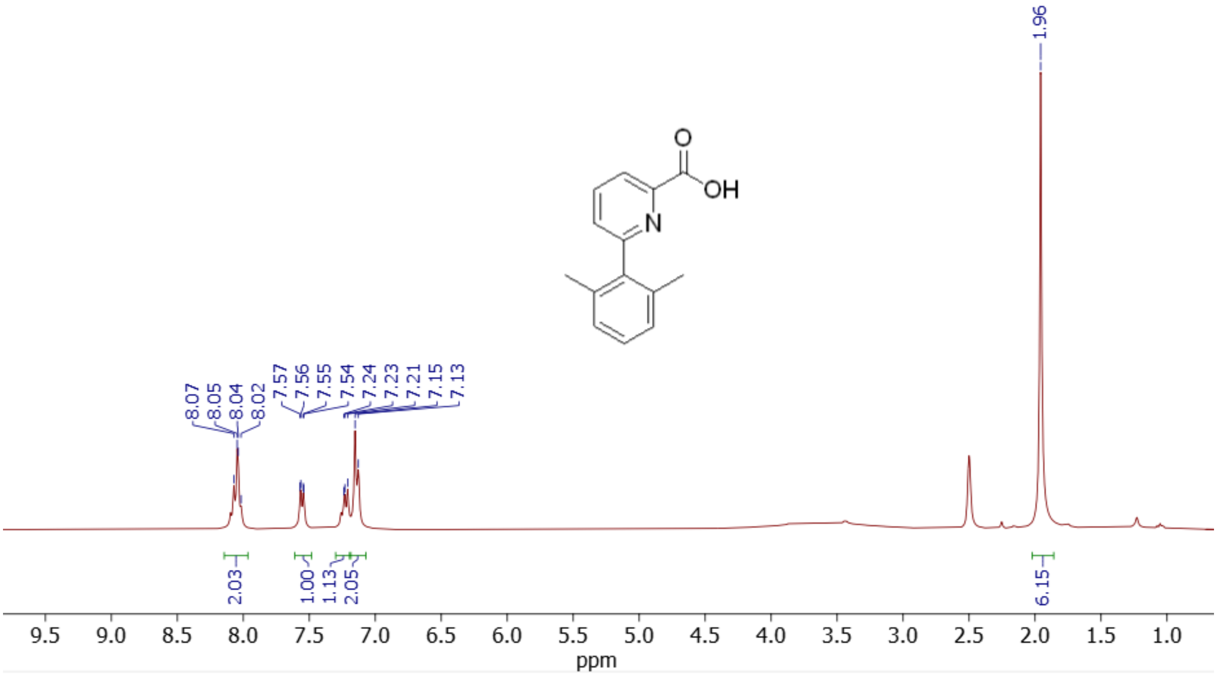

Figure S3. ^{1}H NMR spectrum (DMSO-d_6, 298 K, 300 MHz) of **prec2**.

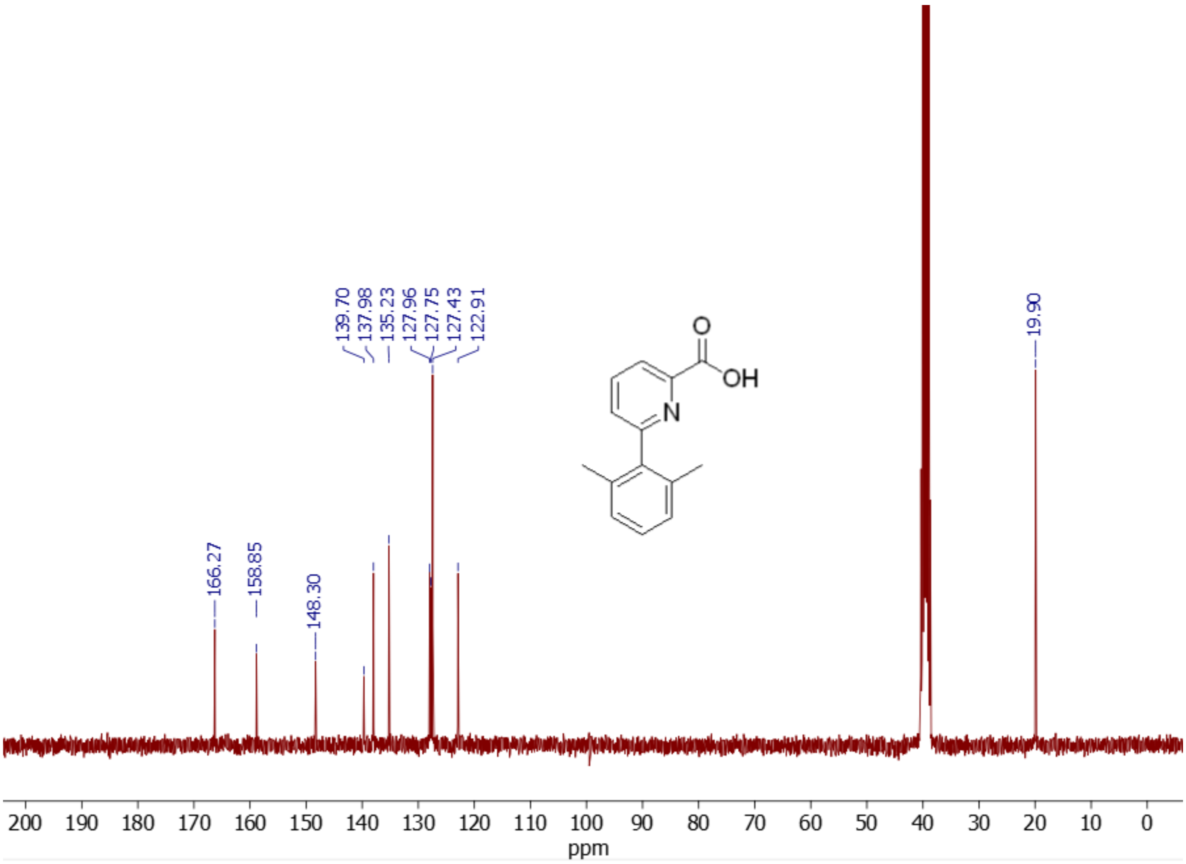

Figure S4. $^{13}C\{^1H\}$ NMR spectrum (DMSO-d_6, 298 K, 75 MHz) of **prec2**.

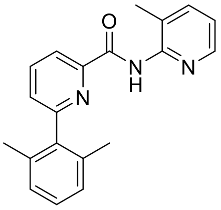

prec3. To a solution of 6-(2,6-dimethylphenyl)picolinic acid (169 mg, 0.744 mmol), PyBOP (464 mg, 0.892 mmol) and 3-methylpyridin-2-amine (83 µL, 0.818 mmol) in THF (6 mL) was added iPr_2EtN (390 µL, 2.23 mmol) and the resulting solution was stirred for 16 h at 50 °C. The reaction mixture was then concentrated under vacuum, dissolved in CH_2Cl_2 (15 mL), washed with brine (15 mL), evaporated and purified by FC (5 to 50% EtOAc/n-hexane; rf = 0.6 in 50% EtOAc/n-hexane) to yield the pure title compound as an off-white solid (189 mg, 80%). ^1H NMR (400 MHz, DMSO-d_6, 298 K): δ = 10.39 (s, 1H, NH), 8.28 (dd, J_{HH} = 4.8, 1.8 Hz, 1H, CH_{PYA}), 8.19 – 8.09 (m, 2H, CH_{pyr}), 7.71 (dd, J_{HH} = 7.1, 1.8 Hz, 1H, CH_{PYA}), 7.61 (dd, J_{HH} = 6.3, 2.6 Hz, 1H, CH_{pyr}), 7.26 – 7.21 (m, 2H, CH_{xyl} +CH_{PYA}), 7.16 (d, $^3J_{HH}$ = 7.5 Hz, 2H, CH_{xyl}), 2.23 (s, 3H, PYA-CCH_3), 2.05 (s, 6H, xyl-CH_3) ppm. $^{13}C\{^1H\}$ NMR (101 MHz, DMSO-d_6, 298 K): δ = 162.64 (*C*O), 158.04 (C_{pyr}-xyl), 149.59 (C_{pyr}-CO or C_{PYA}), 149.41 (C_{pyr}-CO or C_{PYA}), 145.97 (CH_{pyr}), 139.50 (C_{xyl}-pyr), 139.38 (CH_{PYA}), 138.60 (CH_{pyr}), 135.49 (2C, $C_{xyl}CH_3$), 129.18 (PYA*C*CH_3)128.11 (CH_{xyl} or CH_{pyr}), 127.97 (CH_{xyl} or CH_{pyr}), 127.59 (2C, CH_{xyl}), 122.21 (CH_{PYA}), 120.64 (CH_{pyr}), 20.09 (PYACCH_3), 17.59 (2C, xyl-CH_3) ppm. HR-MS (m/z): calculated for $C_{20}H_{19}N_3NaO$ $[M+Na]^+$ = 340.1420; found: 340.1420.

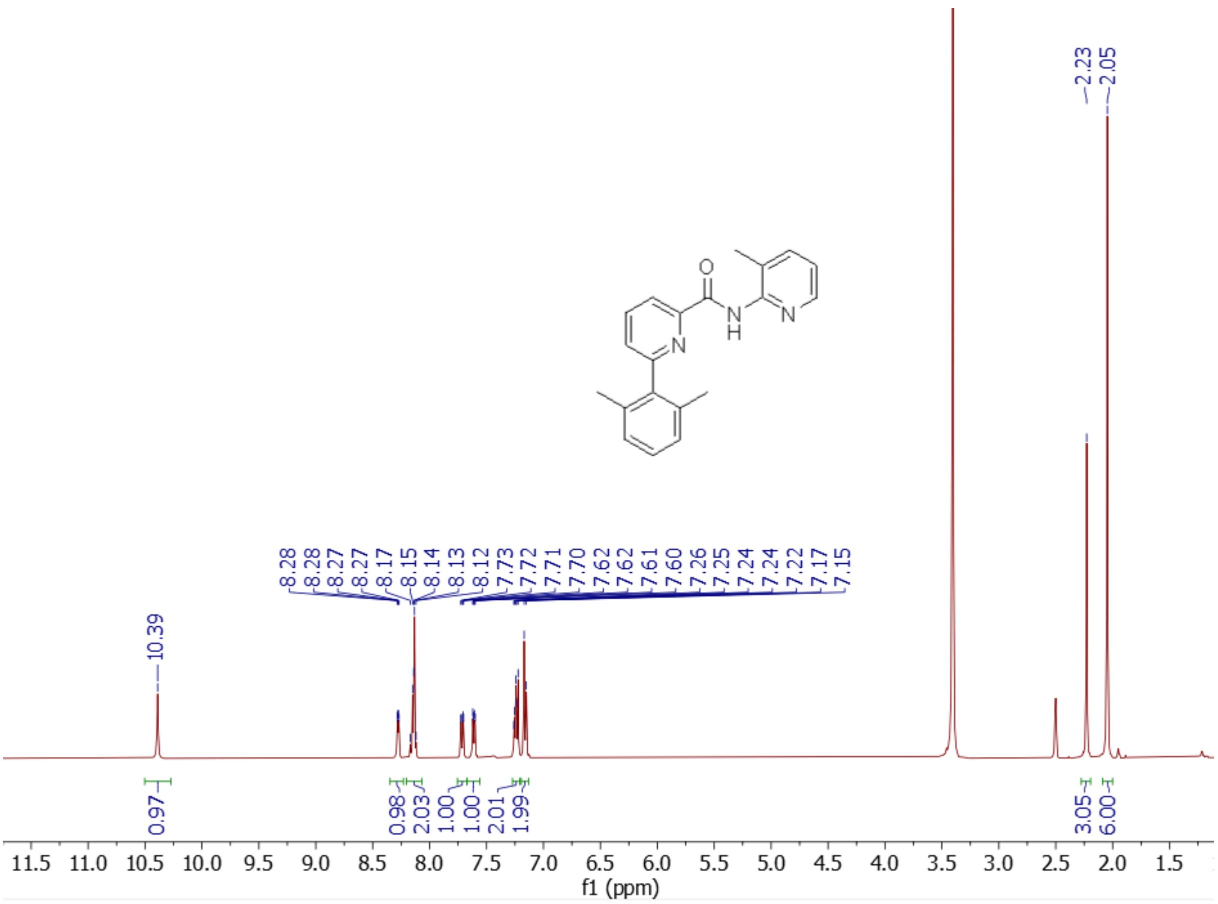

Figure S5. ^{1}H NMR spectrum (DMSO-d_6, 298 K, 400 MHz) of **prec3**.

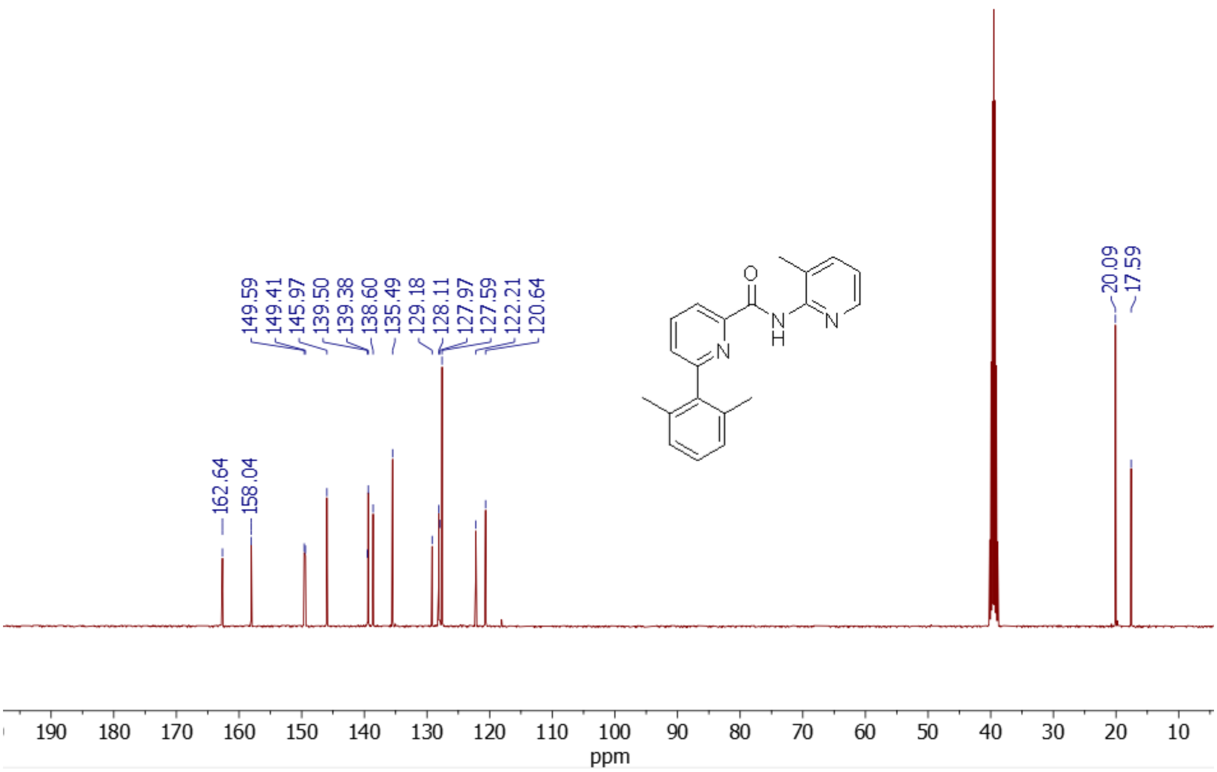

Figure S6. $^{13}C\{^{1}H\}$ NMR spectrum (DMSO-d_6, 298 K, 101 MHz) of **prec3**.

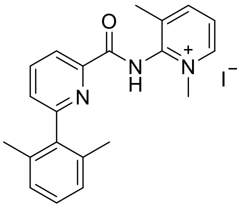

[L_5H]I. To a solution of 6-(2,6-dimethylphenyl)-N-(3-methylpyridin-2-yl)picolinamide (120 mg, 378 µmol) in MeCN (5 mL) was added methyl iodide (47 µL, 756 µmol) and the resulting mixture was stirred for 24 h at 60 °C. The crude mixture was concentrated to 1 mL MeCN then precipitated with Et_2O, filtered, washed with the same solvent and dried under vacuum to yield **[L_5H]**I as a pale-yellow solid (150 mg, 86%). ^{1}H NMR (400 MHz, CD_3CN, 298 K): δ = 10.48 (br s, 1H, N*H*), 8.62 (d, $^{3}J_{HH}$ = 6.1 Hz, 1H, C*H*$_{PYA}$), 8.44 (d, $^{3}J_{HH}$ = 8.0 Hz, 1H, C*H*$_{PYA}$), 8.21 (dd, J_{HH} = 7.8, 1.0 Hz, 1H, C*H*$_{pyr}$), 8.15 (t, $^{3}J_{HH}$ = 7.8 Hz, 1H, C*H*$_{pyr}$), 7.86 (dd, $^{3}J_{HH}$ = 8.0, 6.1 Hz, 1H, C*H*$_{PYA}$), 7.65 (dd, J_{HH} = 7.8, 1.0 Hz, 1H, C*H*$_{pyr}$), 7.27 (dd, $^{3}J_{HH}$ = 8.6, 6.5 Hz, 1H, C*H*$_{xyl}$), 7.19 (d, $^{3}J_{HH}$ = 7.5 Hz, 2H, C*H*$_{xyl}$), 4.20 (s, 3H NC*H*$_3$), 2.43 (s, 3H, pyrC*H*$_3$), 2.11 (s, 6H, xyl-C*H*$_3$) ppm. $^{13}C\{^{1}H\}$ NMR (101 MHz, CD_3CN, 298 K): δ = 164.02 (*C*O), 159.58 (*C*$_{pyr}$-xyl), 148.93 (*C*H$_{pyr}$),), 147.77 (*C*$_{pyr}$-CO), 146.86 (*C*$_{PYA}$), 144.96 (*C*H$_{PYA}$), 139.88 (*C*$_{xyl}$-pyr), 139.30 (*C*H$_{pyr}$), 138.72 (*C*$_{pyr}$-Me), 136.58 (2C, *C*$_{xyl}$CH$_3$), 130.03 (*C*H$_{pyr}$), 129.05 (*C*H$_{xyl}$), 128.31 (2C, *C*H$_{xyl}$),126.18 (*C*H$_{PYA}$), 121.61 (*C*H$_{pyr}$), 45.97 (N*C*H$_3$) , 20.10 (2C, xyl-*C*H$_3$), 17.77 (PYA-C*C*H$_3$) ppm. HR-MS (m/z): calculated for $C_{21}H_{22}N_3O$ $[M\text{-}I]^{+}$ = 332.1757; found: 332.1754.

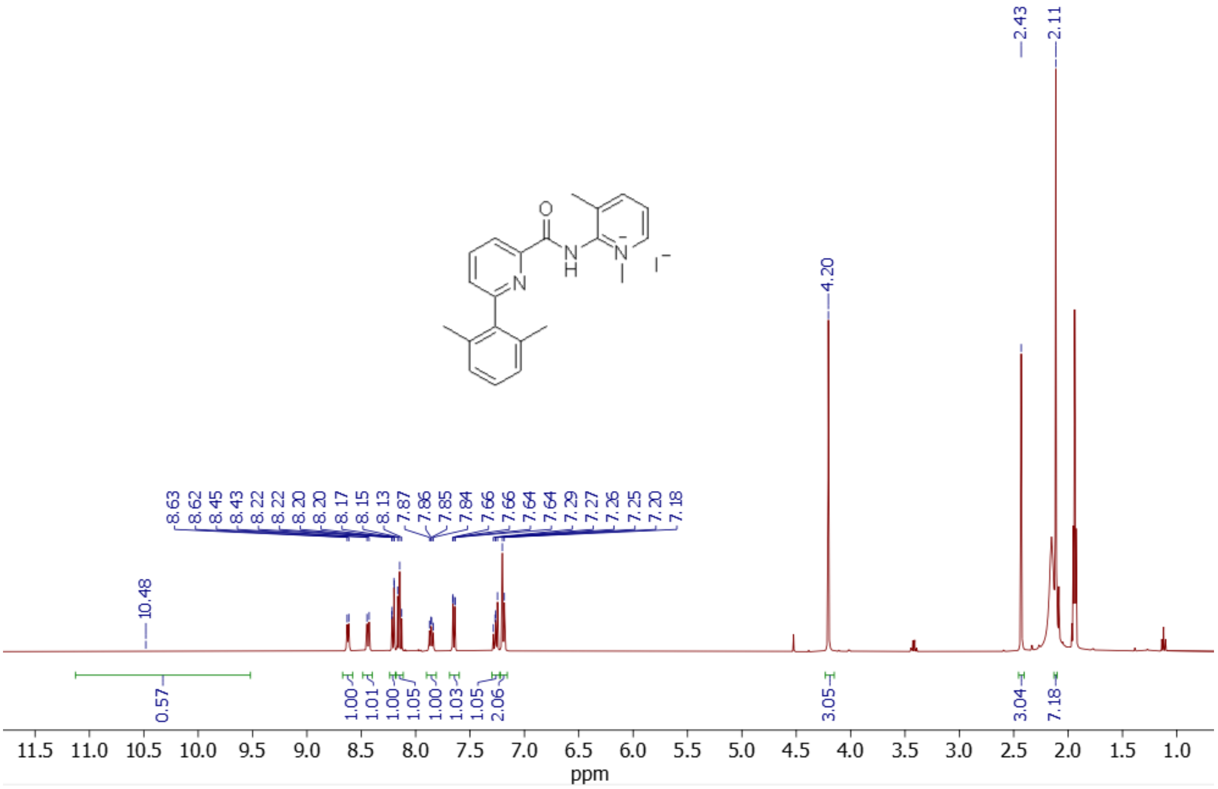

Figure S7. ^{1}H NMR spectrum (CD_3CN, 298 K, 400 MHz) of **[L$_5$H]I**.

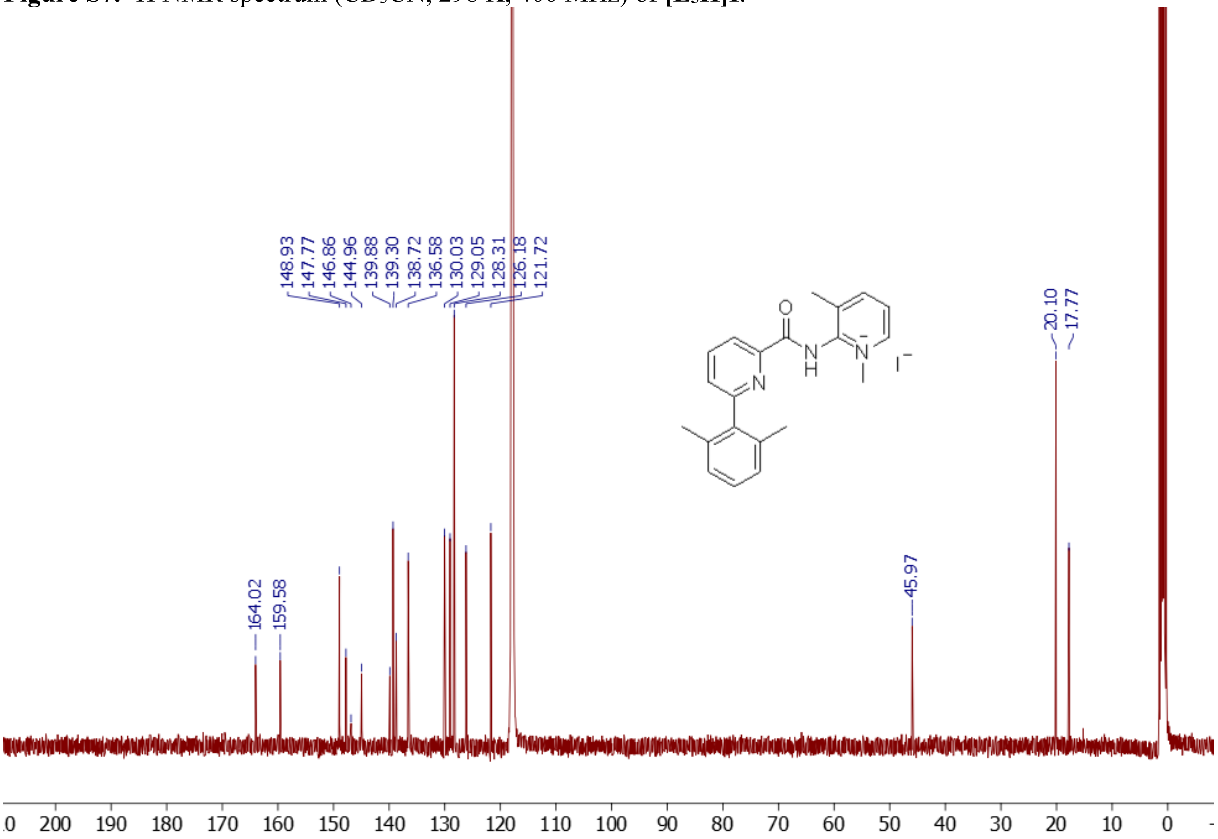

Figure S8. $^{13}C\{^{1}H\}$ NMR spectrum (CD_3CN, 298 K, 101 MHz) of **[L$_5$H]I**.

S.1.3 General procedure for the preparation of neutral pyridylidene amides L_1-L_5, L_8

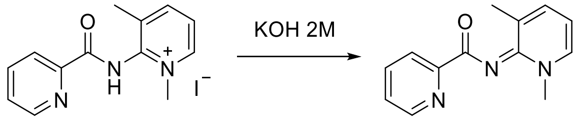

Scheme S2. Preparation of the neutral PYA ligand $\mathbf{L_1}$

The pyridinium salt (2 mmol) was dissolved in aq. KOH (2 M, 10 mL, 20 mmol) and the resulting solution was briefly sonicated and stirred at 23 °C for 10 min. The resulting slurry was extracted with CH_2Cl_2 (5 x 15 mL), the combined organic fractions were dried over $MgSO_4$, filtered and concentrated under vacuo to yield the pure pyridylidene amide (PYA) ligand.

S.1.3.1 Synthesis of neutral PYA ligand L1

Following the general procedure, reaction of **[L₁H]I** (750 mg, 2.1 mmol) yielded compound $\mathbf{L_1}$ as a pale-yellow solid (432 mg, 90%). Crystals suitable for Xray diffraction were grown by slow diffusion of Et_2O in a CH_2Cl_2 solution. ^{1}H NMR (300 MHz, CD_2Cl_2, 298 K): δ = 8.61 (d, $^3J_{HH}$ = 4.6 Hz, 1H, CH_{pyr}), 8.18 (d, $^3J_{HH}$ = 7.4 Hz, 1H, CH_{pyr}), 7.76 (t, $^3J_{HH}$ = 7.4 Hz, 1H, CH_{pyr}), 7.66 (d, $^3J_{HH}$ = 6.6 Hz, 1H, CH_{PYA}), 7.57 (d, $^3J_{HH}$ = 7.2 Hz, 1H, CH_{PYA}), 7.31 (dd, $^3J_{HH}$ = 7.4, 4.6 Hz, 1H, CH_{pyr}), 6.73 (dd, $^3J_{HH}$ = 7.2, 6.7 Hz, 1H, CH_{PYA}), 3.86 (s, 3H, PYA-NCH_3), 2.20 (s, 3H, PYA-CCH_3) ppm. ^{13}C{^{1}H} NMR (75 MHz, CD_2Cl_2, 298 K): δ = 166.73 (*C*O), 161.26 (C_{PYA}), 156.28 (C_{pyr}), 149.06 (*C*H_{pyr}), 140.49 (*C*H_{PYA}), 137.85 (*C*H_{PYA}), 136.47 (*C*H_{pyr}), 132.51 (C_{PYA}–Me), 124.63 (*C*H_{pyr}) 124.13 (*C*H_{pyr}), 113.27 (*C*H_{PYA}), 42.83 (PYA-N*C*H_3), 19.34 (PYA-C*C*H_3) ppm. HR-MS (m/z): calculated for $C_{13}H_{14}N_3O$ $[M+H]^+$ = 228.1137; found: 228.1126. Note: The PF_6 analog **[L₁H]PF₆** was prepared by anion metathesis according to a previously reported procedure.[S1] All analytical data in accordance with reported values.

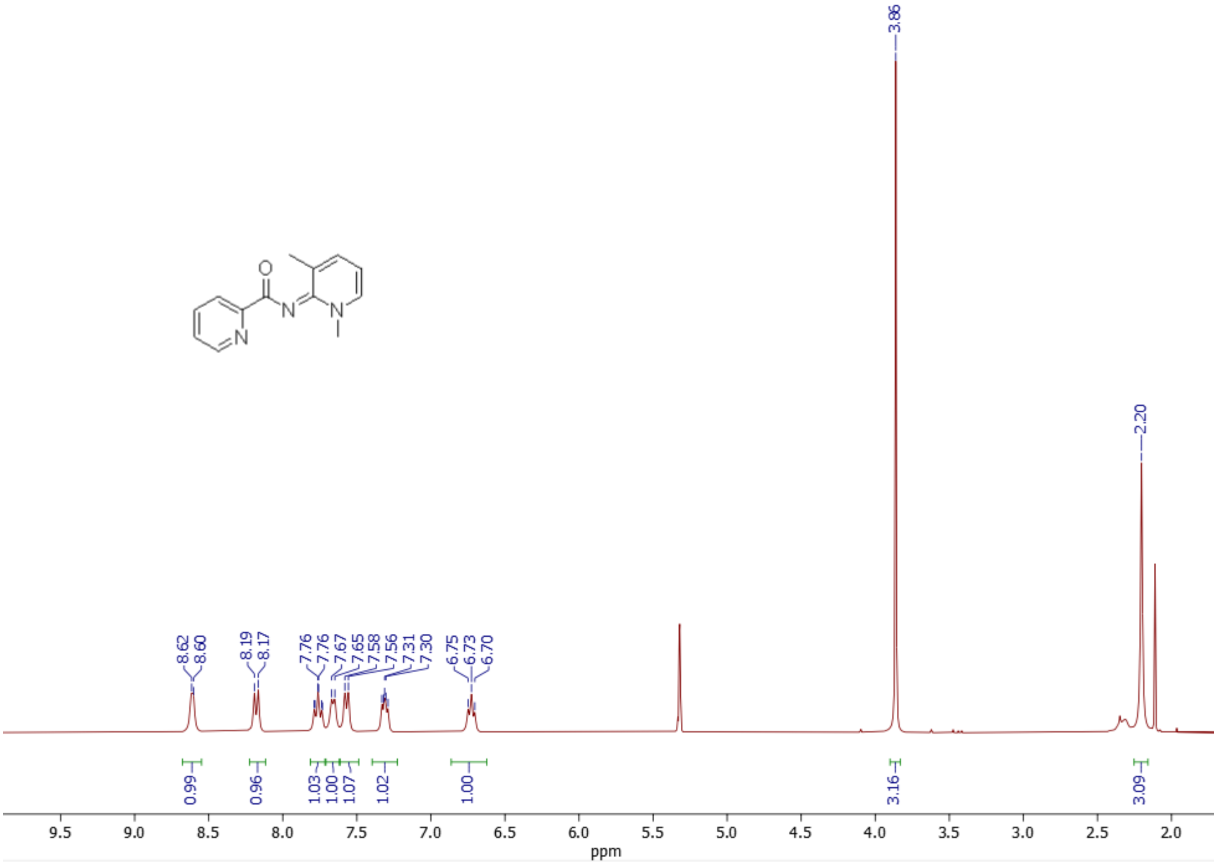

Figure S9. ^{1}H NMR spectrum (CD_2Cl_2, 298 K, 300 MHz) of **L$_1$**.

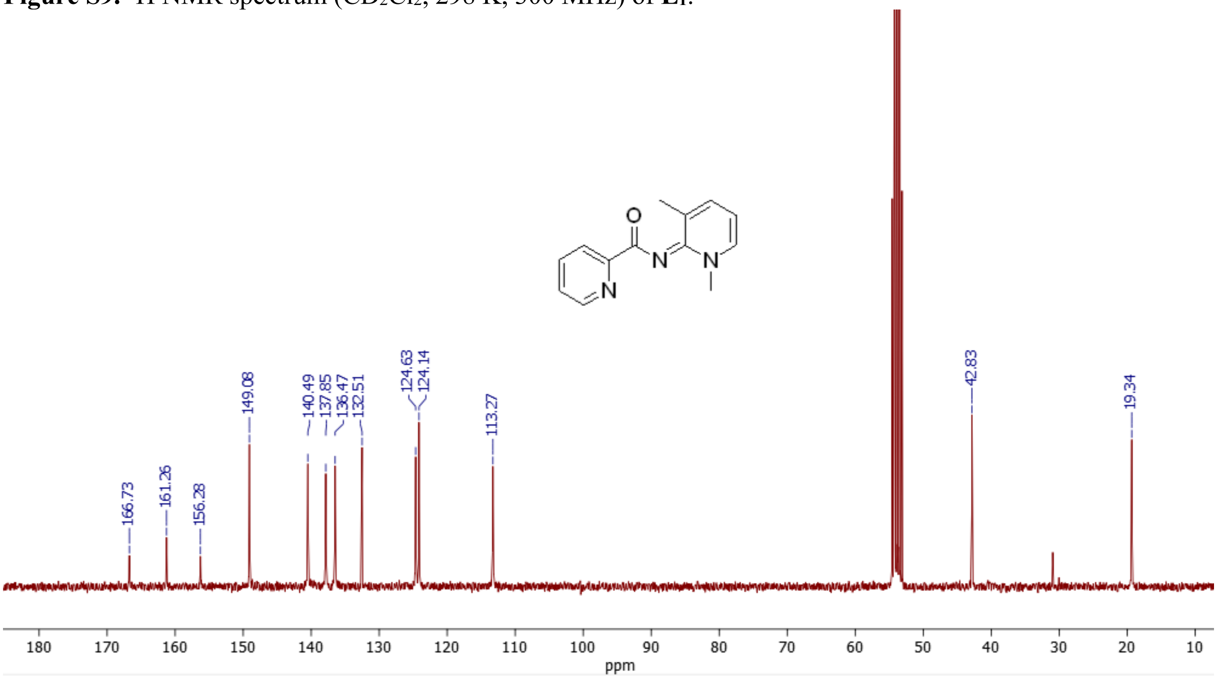

Figure S10. $^{13}C\{^{1}H\}$ NMR spectrum (CD_2Cl_2, 298 K, 75 MHz) of **L$_1$**.

S.1.3.2 Synthesis of neutral PYA ligand L_2

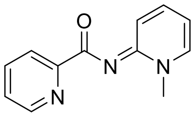

Following the general procedure, reaction of **[L_2H]I** (750 mg, 2.2 mmol) yielded compound **L_2** as a pale red solid that crystallized under vacuo (412 mg, 88%). ^{1}H NMR (400 MHz, CD_2Cl_2, 298 K): δ = 8.66 (d, J_{HH} = 4.6 Hz, 1H, CH_{pyr}), 8.32 – 8.27 (m, 2H, CH_{PYA} + CH_{pyr}), 7.76 (td, J_{HH} = 7.7, 1.8 Hz, 1H, CH_{pyr}), 7.67 (dd, J_{HH} = 6.7, 1.9 Hz, 1H, CH_{PYA}), 7.60 (ddd, J_{HH} = 9,0, 6.7, 1.9 Hz, 1H, CH_{PYA}), 7.31 (ddd, J_{HH} = 7.7, 4.6, 1.3 Hz, 1H, CH_{PYA}), 6.59 (td, J_{HH} = 6.7, 1.5 Hz, 1H, CH_{PYA}), 3.87 (s, 3H, CH_3) ppm. ^{13}C{^{1}H} NMR (101 MHz, CD_2Cl_2, 298 K): δ = 172.46 (*C*O), 160.08 (*C*$_{PYA}$), 156.67 (*C*$_{pyr}$), 149.38 (*C*H_{pyr}), 140.03 (*C*H_{PYA}), 137.84 (*C*H_{PYA}), 136.49 (*C*H_{pyr}), 129.94 (*C*H_{pyr}), 124.62 (*C*H_{pyr}), 121.20 (*C*H_{PYA}), 111.78 (*C*H_{PYA}), 41.92 (N*C*H_3) ppm. HR-MS (m/z): calculated for $C_{12}H_{12}N_3O$ $[M+H]^+$ = 214.0980; found: 214.0969.

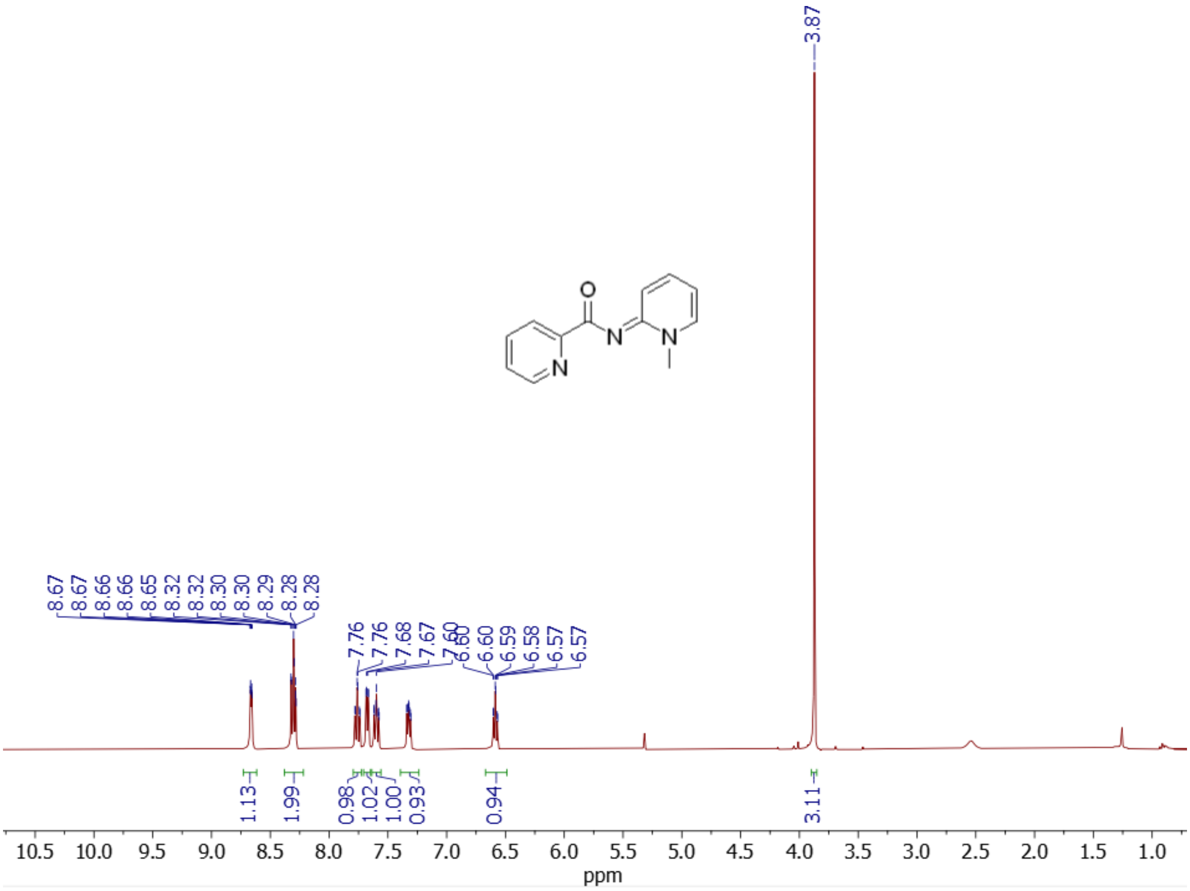

Figure S11. ^{1}H NMR spectrum (CD_2Cl_2, 298 K, 400 MHz) of **L_2**.

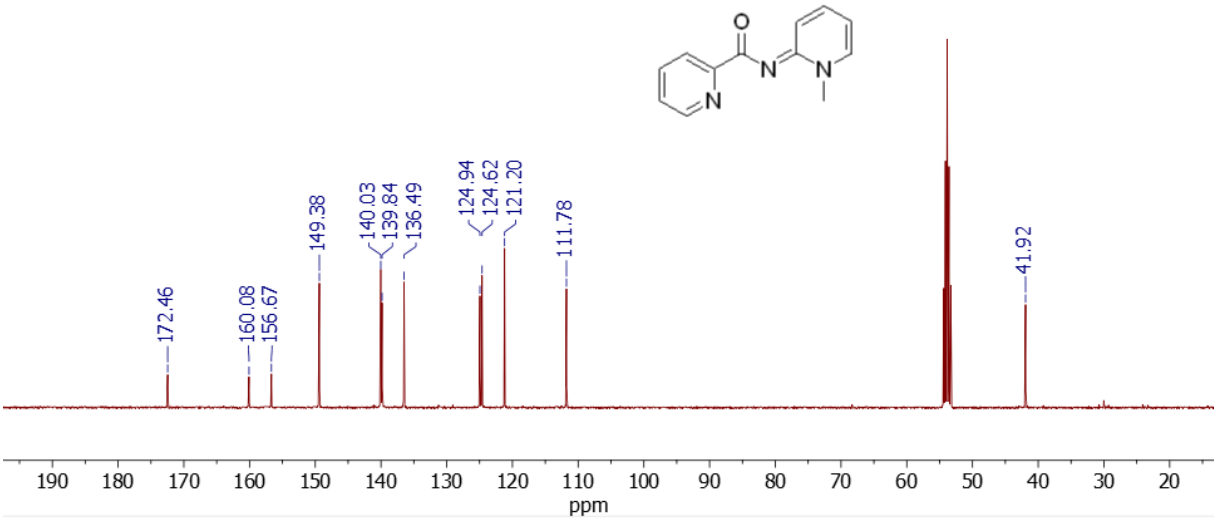

Figure S12. $^{13}C\{^1H\}$ NMR spectrum (CD_2Cl_2, 298 K, 101 MHz) of $\mathbf{L_2}$.

S.1.3.3 Synthesis of neutral PYA ligand L$_3$

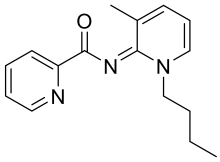

To a solution of N-(3-methylpyridin-2-yl)picolinamide (1.04g, 4.88 mmol) in dry MeCN (16 mL) in a pressure tube was added nBuI (2.8 mL, 24.4 mmol). The resulting mixture was stirred 24 h at 120 °C. Solvent amount was then reduced to 5 mL and Et_2O was added. The suspension was filtered and dried. The crude yellow solid was dissolved in a KOH solution (2 M, 25.0 mL, 50.0 mmol). The resulting slurry was stirred 5 min at 23 °C and then extracted with CH_2Cl_2 (3 x 20 mL). The combined organic fractions were concentrated under vacuum and purified by gradient flash chromatography (0 to 10% MeOH in CH_2Cl_2 on Al_2O_3; rf 0.7 in 10% MeOH/ CH_2Cl_2) to yield $\mathbf{L_3}$ as a pale brown waxy solid (1.28 g, 66%) ^{1}H NMR (400 MHz, CD_2Cl_2, 298 K): δ = 8.63 (dd, J_{HH} = 4.8, 1.8 Hz, 1H, CH_{pyr}), 8.16 (d, $^3J_{HH}$ = 7.8 Hz, 1H, CH_{pyr}), 7.76 (td, J_{HH} = 7.8, 1.8 Hz, 1H, CH_{pyr}), 7.61 (d, $^3J_{HH}$ = 6.6 Hz, 1H, CH_{PYA}), 7.56 (d, $^3J_{HH}$ = 7.0 Hz, 1H, CH_{PYA}), 7.31 (dd, $^3J_{HH}$ = 7.8, 4.8 Hz, 1H, CH_{pyr}), 6.74 (dd, $^3J_{HH}$ = 7.8, 6.6 Hz, 1H, CH_{PYA}), 4.30 (t, $^3J_{HH}$ = 7.4 Hz, 2H, NCH_2), 2.20 (s, 3H, PYA-CCH_3), 1.82 (apparent quintet, $^3J_{HH}$ = 7.4 Hz, 2H, NCH_2CH_2), 1.36 (apparent sextet, $^3J_{HH}$ = 7.4 Hz, 2H, CH_2CH_3), 0.91 (t, $^3J_{HH}$ = 7.4 Hz, 3H, CH_2CH_3)ppm. $^{13}C\{^1H\}$ NMR (101 MHz, CD_2Cl_2, 298 K): δ = 166.89 (*C*O), 161.20 (C_{PYA}), 156.52 (C_{pyr}), 149.20 (CH_{pyr}), 140.56 (CH_{PYA}), 137.00 (CH_{PYA}), 136.41 (CH_{pyr}), 132.79 (C_{PYA}–Me), 124.54 (CH_{pyr}) 124.17 (CH_{pyr}), 113.34 (CH_{PYA}), 54.88 (NCH_2), 31.92 (NCH2CH_2), 20.21 (CH_2CH_3), 19.46 (PYA-CCH_3), 13.79 (CH_2CH_3) ppm. HR-MS (m/z): calculated for $C_{16}H_{20}N_3O$ $[M+H]^+$ = 270.1606; found: 270.1596.

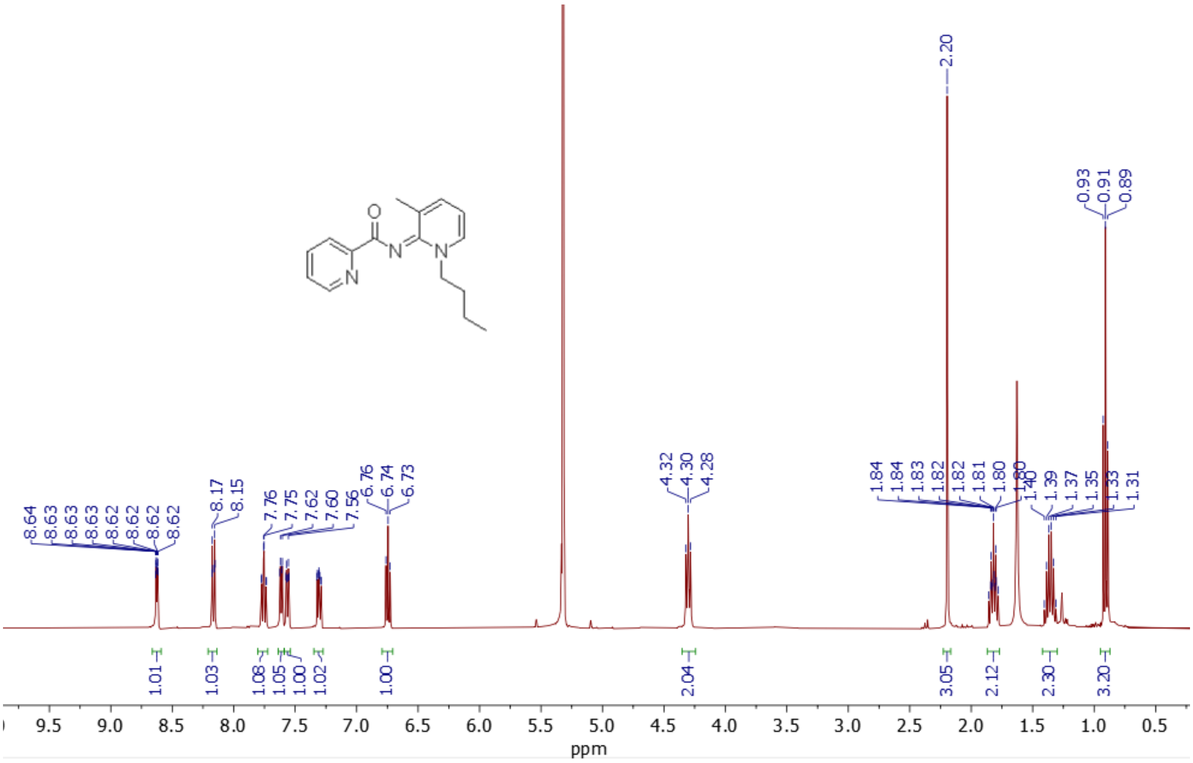

Figure S13. ^{1}H NMR spectrum (CD_2Cl_2, 298 K, 400 MHz) of **L3**.

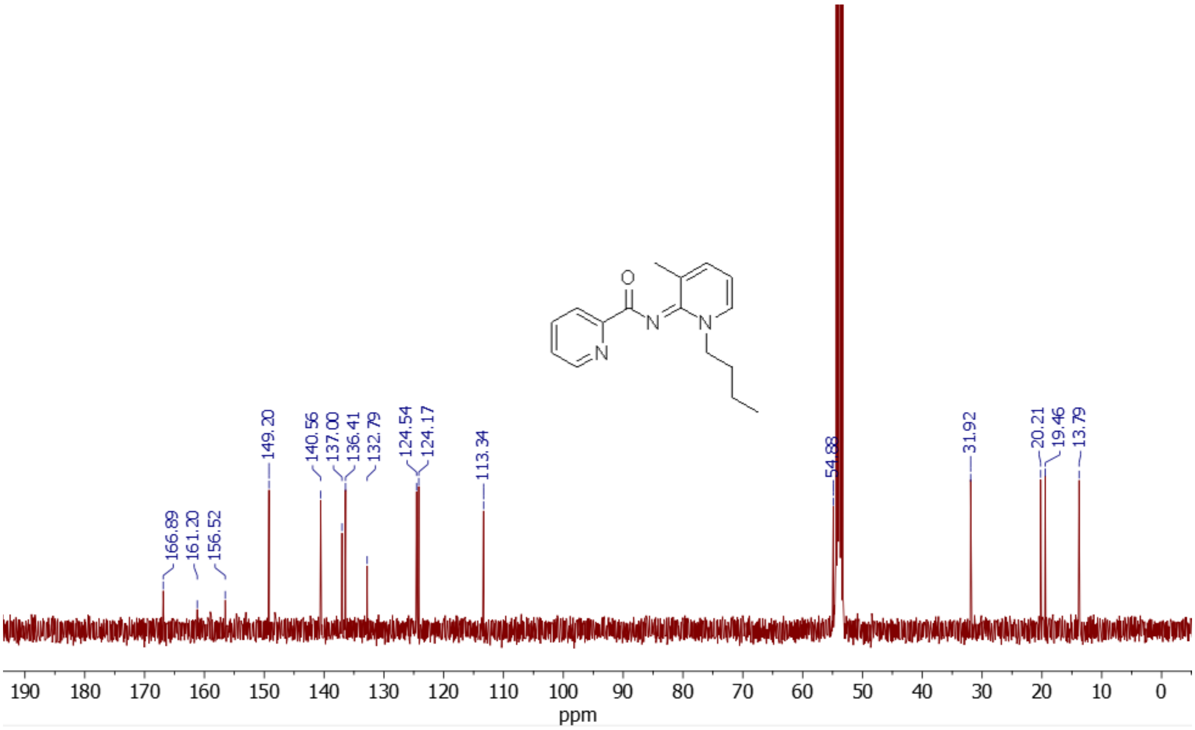

Figure S14. $^{13}C\{^{1}H\}$ NMR spectrum (CD_2Cl_2, 298 K, 101 MHz) of **L3**.

S.1.3.4 Synthesis of neutral PYA ligand L_4

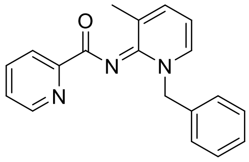

To a solution of N-(3-methylpyridin-2-yl)picolinamide (1.0 g, 4.68 mmol) in dry MeCN (20 mL) in a pressure tube was added benzyl chloride (10.8 mL, 93.8 mmol). The resulting mixture was stirred for 3 days at 85 °C. The solvent was evaporated and the residue was triturated into Et_2O, filtered and recrystallized from MeCN/Et_2O at –20 °C. The precipitate was filtered, washed with Et_2O and dried to yield a pale-yellow solid, which was dissolved in a KOH solution (2 M, 25 mL, 50 mmol). The resulting slurry was stirred for 5 min at 23 °C and then extracted with CH_2Cl_2 (3 x 20 mL). The combined organic fractions were dried over $MgSO_4$, filtered and concentrated under vacuum to yield **L_4** as a pale brown waxy solid (653 mg, 46%) that precipitated upon drying for several days under vacuum. ^{1}H NMR (400 MHz, CD_2Cl_2, 298 K): δ = 8.63 (dd, J_{HH} = 4.7, 1.5 Hz, 1H, CH_{pyr}), 8.12 (dd, J_{HH} = 7.9, 1.1 Hz, 1H, CH_{pyr}), 7.74 (td, J_{HH} = 7.7, 1.8 Hz, 1H, CH_{pyr}), 7.62 (d, $^3J_{HH}$ = 6.8 Hz, 1H, CH_{PYA}), 7.56 (d, $^3J_{HH}$ = 6.8 Hz, 1H, CH_{PYA}), 7.46 - 7.22 (m, 6H, CH_{pyr} + 5 CH_{phenyl}), 6.72 (t, $^3J_{HH}$ = 6.8 Hz, 1H, CH_{PYA}), 5.53 (s, 2H, NCH_2), 2.22 (s, 3H, PYA-CCH_3) ppm. ^{13}C{^{1}H} NMR (101 MHz, CD_2Cl_2, 298 K): δ = 167.20 (CO), 161.16 (C_{PYA}), 156.21 (C_{pyr}), 149.21 (CH_{pyr}), 140.69 (CH_{PYA}), 136.71 (CH_{pyr}), 136.48 (CH_{PYA}), 135.86 (C_{phenyl}), 132.79 (PYA-CCH_3), 129.33 (2C, CH_{phenyl}), 128.91 (2C, CH_{phenyl}), 128.73 (CH_{phenyl}), 124.69 (CH_{pyr}) 124.27 (CH_{pyr}), 113.35 (CH_{PYA}), 56.97 (NCH_2), 19.70 (PYA-CCH_3), ppm. HR-MS (m/z): calculated for $C_{19}H_{18}N_3O$ $[M+H]^+$ = 304.1450; found: 304.1441.

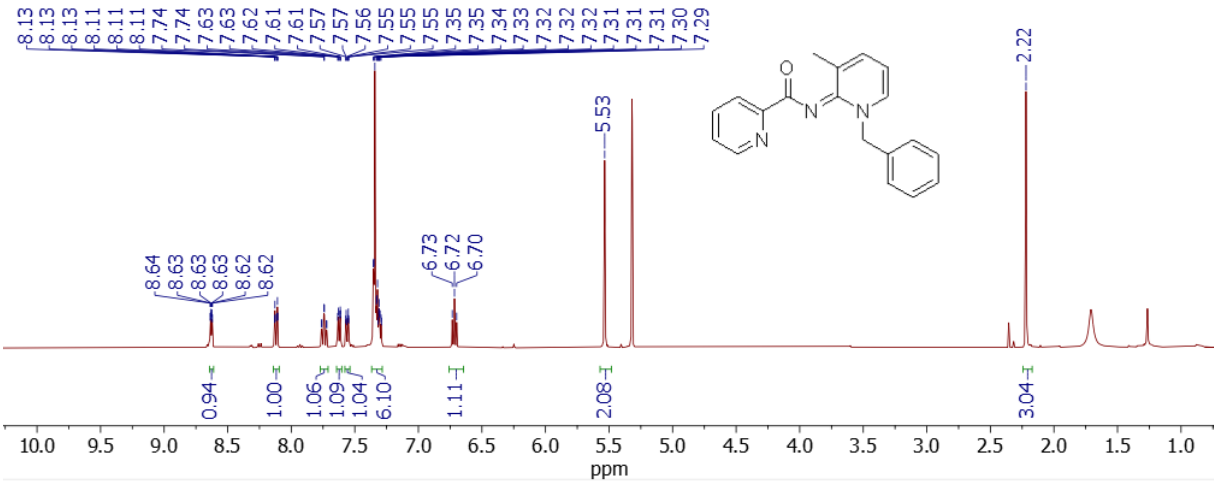

Figure S15. ^{1}H NMR spectrum (CD_2Cl_2, 298 K, 400 MHz) of **L_4**.

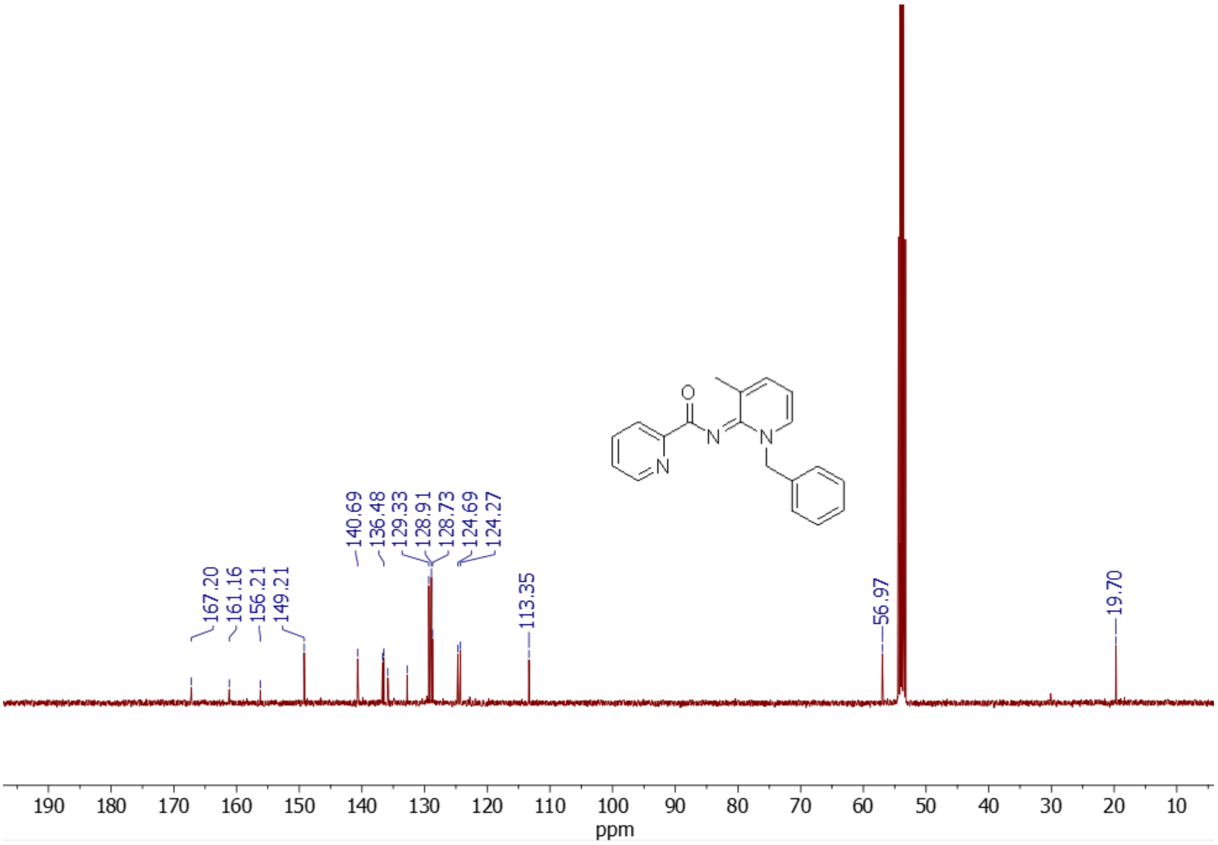

Figure S16. $^{13}C\{^{1}H\}$ NMR spectrum (CD_2Cl_2, 298 K, 101 MHz) of $\mathbf{L_4}$.

S.1.3.5 Synthesis of neutral PYA ligand L_5

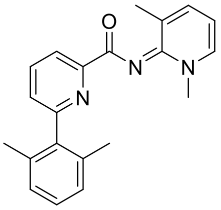

Following the general procedure, reaction of **[L_5H]I** (120 mg, 0.26 mmol) yielded compound **L_5** as an off-white solid (72 mg, 83%). ^{1}H NMR (400 MHz, CD_2Cl_2, 298 K): δ = 8.14 (dd, J_{HH} = 7.6, 1.1 Hz, 1H, CH_{pyr}), 7.85 (t, $^{3}J_{HH}$ = 7.6 Hz, 1H, CH_{pyr}), 7.56 (d, $^{3}J_{HH}$ = 6.6 Hz, 1H, CH_{PYA}), 7.51 (d, $^{3}J_{HH}$ = 7.1 Hz, 1H, CH_{PYA}), 7.25 (dd, $^{3}J_{HH}$ = 7.4, 1.1 Hz, 1H, CH_{pyr}), 7.17 (dd, $^{3}J_{HH}$ = 8.3, 6.7 Hz, 1H, CH_{xyl}), 7.08 (d, $^{3}J_{HH}$ = 7.5 Hz, 2H, CH_{xyl}), 6.64 (dd, $^{3}J_{HH}$ = 7.1, 6.6 Hz, 1H, CH_{PYA}), 3.83 (s, 3H, NCH_3), 2.20 (s, 3H, PYA-CCH_3), 2.02 (s, 6H, xyl-CH_3) ppm. $^{13}C\{^{1}H\}$ NMR (101 MHz, CD_2Cl_2, 298 K): δ = 166.90 (*C*O), 161.03 (C_{PYA}), 158.73 (C_{pyr}-xyl), 156.07 (C_{pyr}CO), 141.19 (C_{xyl}-pyr), 140.11 (*C*H_{PYA}), 137.57 (*C*H_{PYA}), 136.91 (*C*H_{pyr}), 136.29 (xyl-*C*CH_3, 2C) 132.40 (PYA-*C*CH3), 127.98 (*C*H_{xyl}), 127.71 (2C, *C*H_{xyl}), 125.48 (*C*H_{pyr}), 121.91 (*C*H_{pyr}), 112.85 (*C*H_{PYA}), 42.71 (N*C*H_3), 20.40 (2C, xyl-CH_3) 19.34 (PYA-C*C*H_3) ppm. HR-MS (m/z): calculated for $C_{21}H_{22}N_3O$ $[M+H]^{+}$ = 332.1763; found: 332.1751.

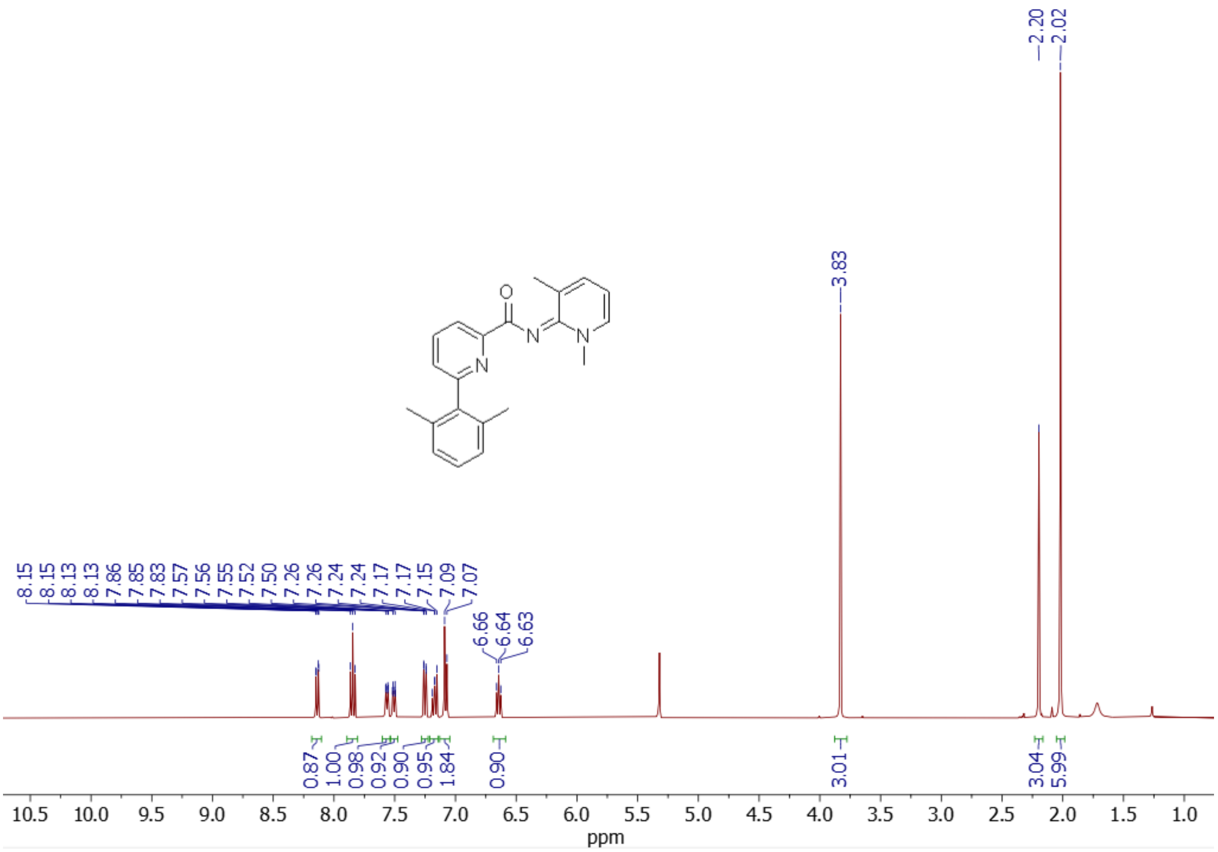

Figure S17. ^{1}H NMR spectrum (CD_2Cl_2, 298 K, 400 MHz) of $\mathbf{L_5}$.

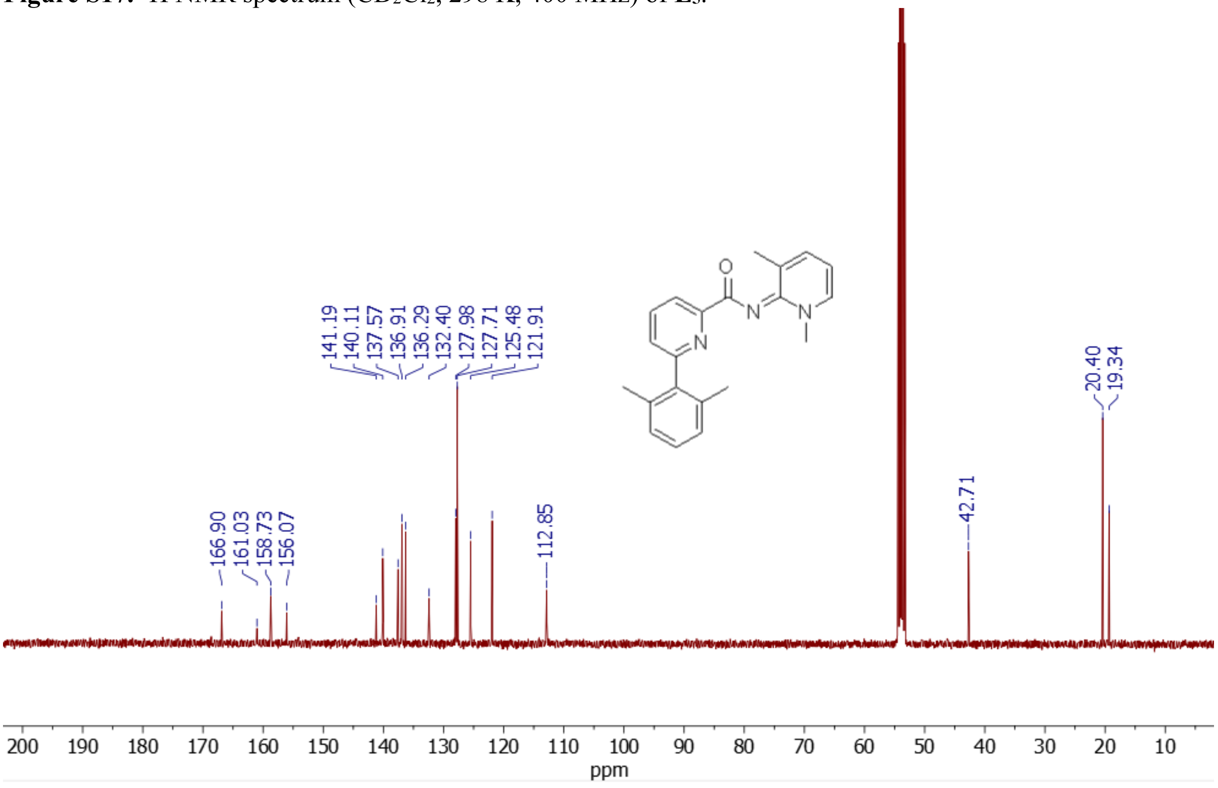

Figure S18. $^{13}C\{^{1}H\}$ NMR spectrum (CD_2Cl_2, 298 K, 101 MHz) of $\mathbf{L_5}$.

S.1.3.6 Synthesis of neutral ligand L_8

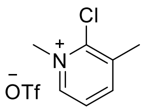

prec4. To a solution of 2-chloro-3-methylpyridine (3.0 mL, 27.5 mmol) in CH_2Cl_2 (30mL) was added methyl trifluoromethanesulfonate (4.7 mL, 41.2 mmol) dropwise and the resulting solution was stirred for 2 h at 23 °C. The solution was then reduced to 10 mL and precipitated with Et_2O (50mL). The precipitate was filtered, washed with the same solvent and dried to yield the pure title compound as a white solid (7.88 g, 98%). ^{1}H NMR (400 MHz, DMSO-d_6, 298 K): δ = 9.07 (d, $^3J_{HH}$ = 6.2 Hz, 1H, CH_{pyr}), 8.57 (d, $^3J_{HH}$ = 7.8 Hz, 1H, CH_{pyr}), 8.01 (dd, $^3J_{HH}$ = 7.8, 6.2 Hz, 1H, CH_{pyr}), 4.34 (s, 3H, NCH_3), 2.54 (s, 3H, CCH_3) ppm. ^{13}C{^{1}H} NMR (101 MHz, DMSO-d_6, 298 K): δ = 147.02 (CH$_{pyr}$), 146.83 (CCl), 145.99 (CH$_{pyr}$), 138.18 (CCH$_3$), 125.06 (CH$_{pyr}$), 120.66 (q, $^1J_{CF}$ = 322.2 Hz, CF$_3$), 48.02 (NCH$_3$), 19.28 (CCH$_3$) ppm. ^{19}F{^{1}H} NMR (376 MHz, DMSO-d_6, 298 K): δ = -79.34 ppm. HR-MS (m/z): calculated for C_7H_9ClN [M-OTf]$^+$ = 142.0418; found: 142.0413.

.

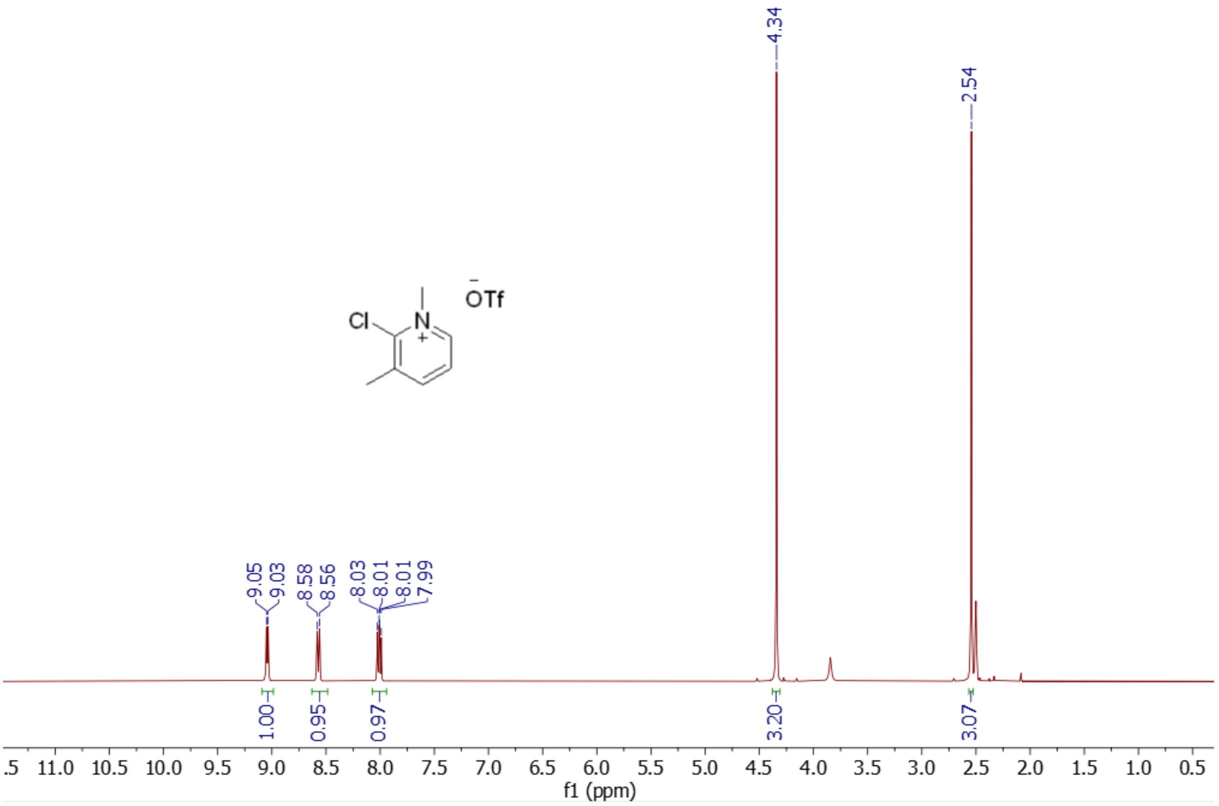

Figure S19. ^{1}H NMR spectrum (DMSO-d_6, 298 K, 300 MHz) of **prec4**.

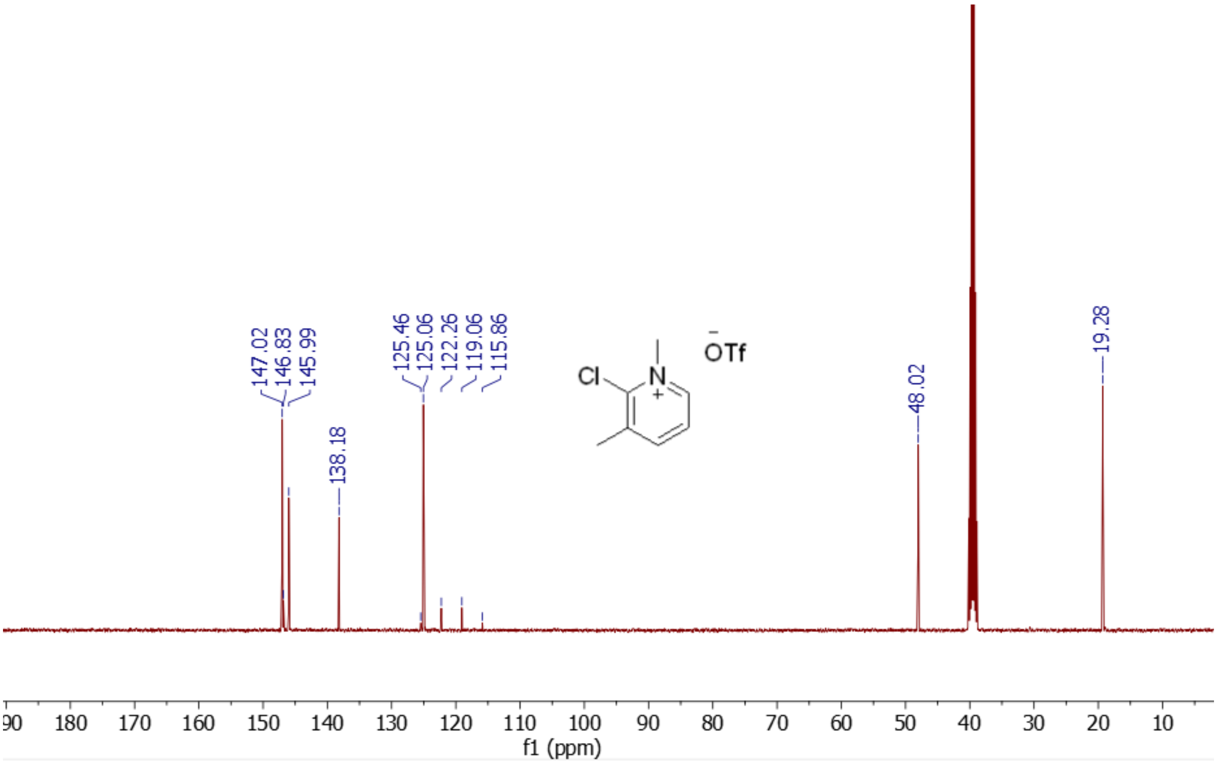

Figure S20. $^{13}C\{^1H\}$ NMR spectrum (DMSO-d_6, 298 K, 75 MHz) of **prec4**.

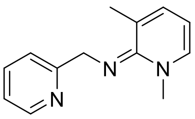

L_8 To a suspension of **prec4** (2.83 g, 9.71 mmol) and K_2CO_3 (1.48 g, 10.68 mmol) in MeCN (8 mL) was added pyridin-2-ylmethanamine (1 mL, 9.71 mmol) and the reaction mixture was stirred 16 h at 80 °C. The resulting suspension was filtered and washed with MeCN. The solvent was then evaporated under vacuum and the residue was dissolved in aq. KOH (2M, 50 mL, 100 mmol) and stirred 5 min before being extracted in CH_2Cl_2 (5 x 20 mL). Organics were dried over $MgSO_4$, filtered and evaporated to yield **L_8** as a brownish oil (2.03 g, 98%). ^1H NMR (400 MHz, CD_2Cl_2, 298 K): δ = 8.46 (dd, J_{HH} = 4.8, 1.6 Hz, 1H, CH_{pyr}), 7.71 (d, $^3J_{HH}$ = 7.4 Hz, 1H, CH_{pyr}), 7.67 (td, J_{HH} = 7.4, 1.6 Hz, 1H, CH_{pyr}), 7.11 (d, J_{HH} = 7.4, 4.8, 1.6 Hz, 1H, CH_{pyr}) 6.97 (dd, J_{HH} = 6.7, 1.8 Hz, 1H, CH_{PYE}), 6.63 (dd, $^3J_{HH}$ = 6.7, 1.8 Hz, 1H, CH_{PYE}), 5.55 (t, $^3J_{HH}$ = 6.7 Hz, 1H, CH_{PYE}), 5.15 (s, 2H, CH_2), 3.40 (s, 3H, PYE-NCH_3), 2.41 (s, 3H, PYE-CCH_3) ppm. $^{13}C\{^1H\}$ NMR (101 MHz, CD_2Cl_2, 298 K): δ = 164.88 (C_{pyr}), 152.69 (C_{PYE}), 148.80 (CH_{pyr}), 137.90 (CH_{PYE}), 136.60 (CH_{pyr}), 135.98 (CH_{PYE}), 125.72 (C_{PYA}–Me), 121.48 (CH_{pyr}) 121.32 (CH_{pyr}), 100.68 (CH_{PYE}), 55.15 (CH_2), 41.32 (PYE-NCH_3), 22.90 (PYE-CCH_3) ppm. HR-MS (m/z): calculated for $C_{13}H_{16}N_3$ $[M+H]^+$ = 214.1344; found: 214.1336.

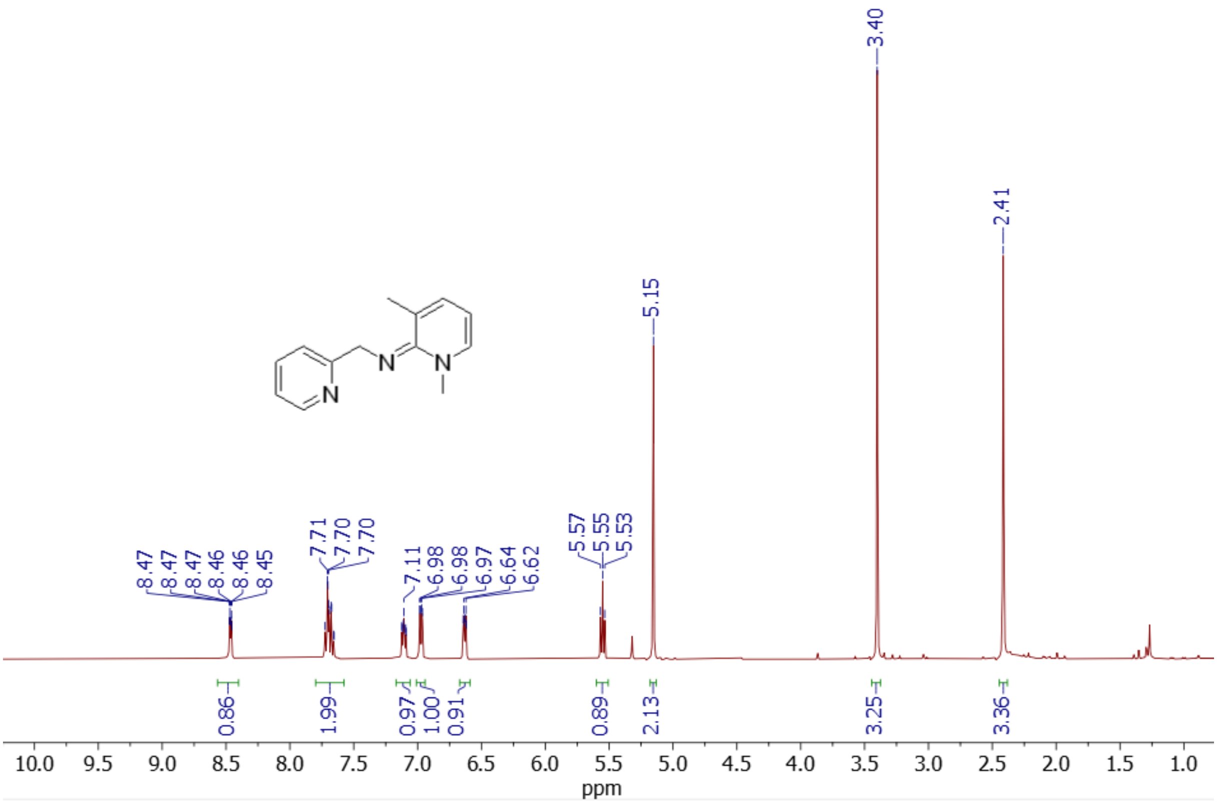

Figure S21. ^{1}H NMR spectrum (CD_2Cl_2, 298 K, 400 MHz) of **L$_8$**.

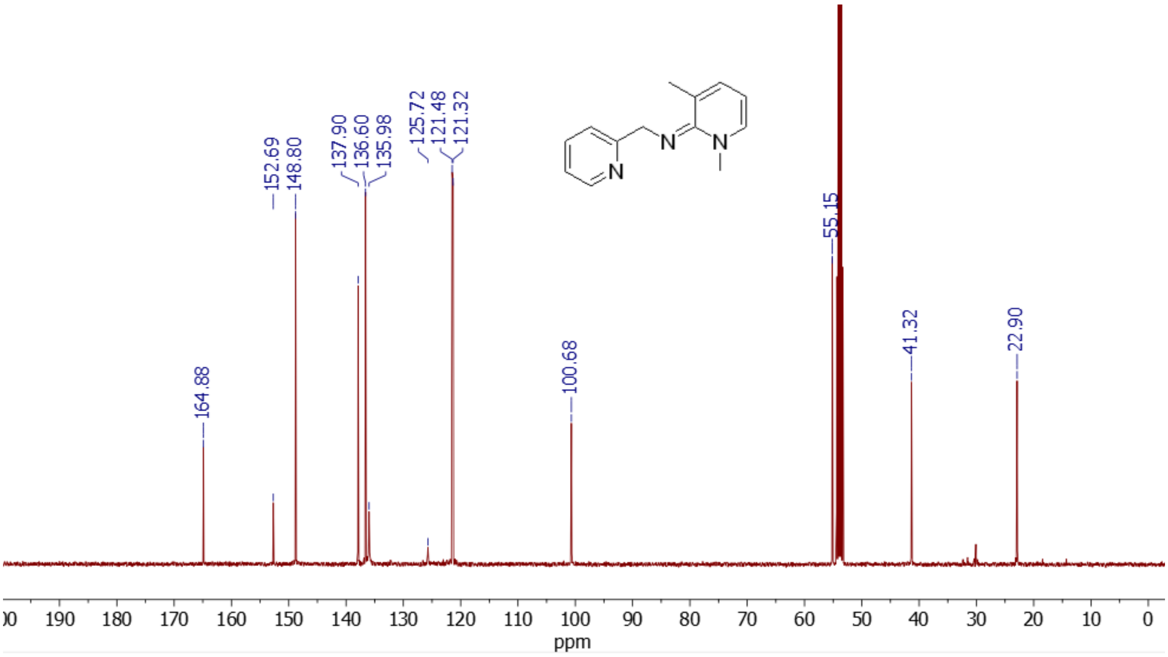

Figure S22. $^{13}C\{^{1}H\}$ NMR spectrum (CD_2Cl_2, 298 K, 101 MHz) of **8**.

S.1.4 General procedure for the preparation of PYA palladium complexes 1-8

The neutral PYA ligand (0.80 to 0.84 mmol, 1.00 to 1.05 eq.) and [Pd(cod)Cl_2] (212 mg, 0.8 mmol, 1 eq.) were dissolved in dry CH_2Cl_2 (5 mL). The resulting yellow mixture was stirred for 2 h at 23 °C and then concentrated to 2 mL. Upon addition of Et_2O (6 mL) a precipitate formed, which was filtered and washed with more Et_2O. The precipitation was repeated if necessary until no palladium precursor was detected. Then the precipitate was dried under vacuo to afford the complex as a bright yellow solid.

S.1.4.1 Synthesis of pyridyl-PYA Pd complex 1

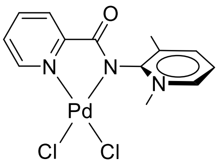

Following the general procedure reaction of **L₁** (270 mg, 1.19 mmol) and [Pd(cod)Cl_2] (308 mg, 1.08 mmol) yielded **1** as a bright orange solid (424 mg, 97%). Crystals suitable for Xray diffraction were grown by slow diffusion of Et_2O in a CH_2Cl_2 solution. ^{1}H NMR (400 MHz, CD_3CN, 298 K): δ = 9.15 (d, J_{HH} = 5.6, 1.5 Hz, 1H, CH_{pyr}), 8.38 (d, $^3J_{HH}$ = 6.2, 1H, CH_{PYA}), 8.23 (d, $^3J_{HH}$ = 7.7, 1H, CH_{PYA}), 8.16 (td, J_{HH} = 7.7, 1.5 Hz, 1H, CH_{pyr}), 7.88 (dd, J_{HH} = 7.7, 1.6 Hz, 1H, CH_{pyr}), 7.69 (ddd, J_{HH} = 7.7, 5.6, 1.6 Hz, 1H, CH_{pyr}), 7.60 (d, $^3J_{HH}$ = 7.7, 6.2, 1H, CH_{PYA}), 4.26 (s, 3H, NCH_3), 2.49 (s, 3H, PYA-CCH_3). ^{13}C{^{1}H} NMR (101 MHz, CD_3CN , 298 K): δ = 169.91 (*C*O), 158.25 (C_{PYA}), 152.62 (C_{pyr}), 149.67 (*C*H_{pyr}), 146.69 (*C*H_{PYA}), 142.77 (*C*H_{PYA}), 141.45 (*C*H_{pyr}), 138.70 (PYA-*C*CH_3), 129.31 (*C*H_{pyr}), 126.93 (*C*H_{pyr}), 123.94 (*C*H_{PYA}), 45.49 (N*C*H_3), 18.38 (PYA-*C*H_3), ppm. HR-MS (m/z): calculated for $C_{13}H_{13}Cl_2N_3NaOPd$ $[M+Na]^+$ = 425.9368; found: 425.9370. Elemental analysis calculated for $C_{13}H_{13}Cl_2N_3OPd \cdot 0.25\ CH_2Cl_2$ (%): C 37.37, H 3.20, N 9.87; found: C 37.69, H 3.30, N 9.79.

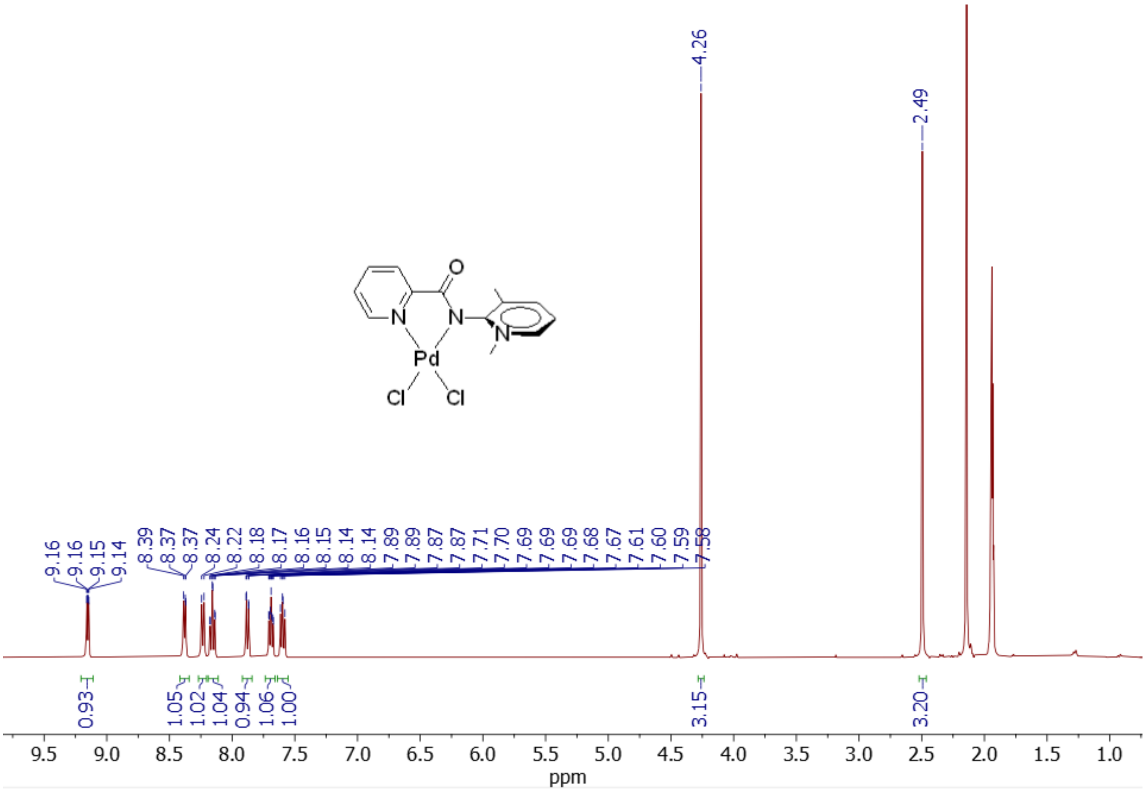

Figure S23. ^{1}H NMR spectrum (CD_3CN, 298 K, 400 MHz) of **1**.

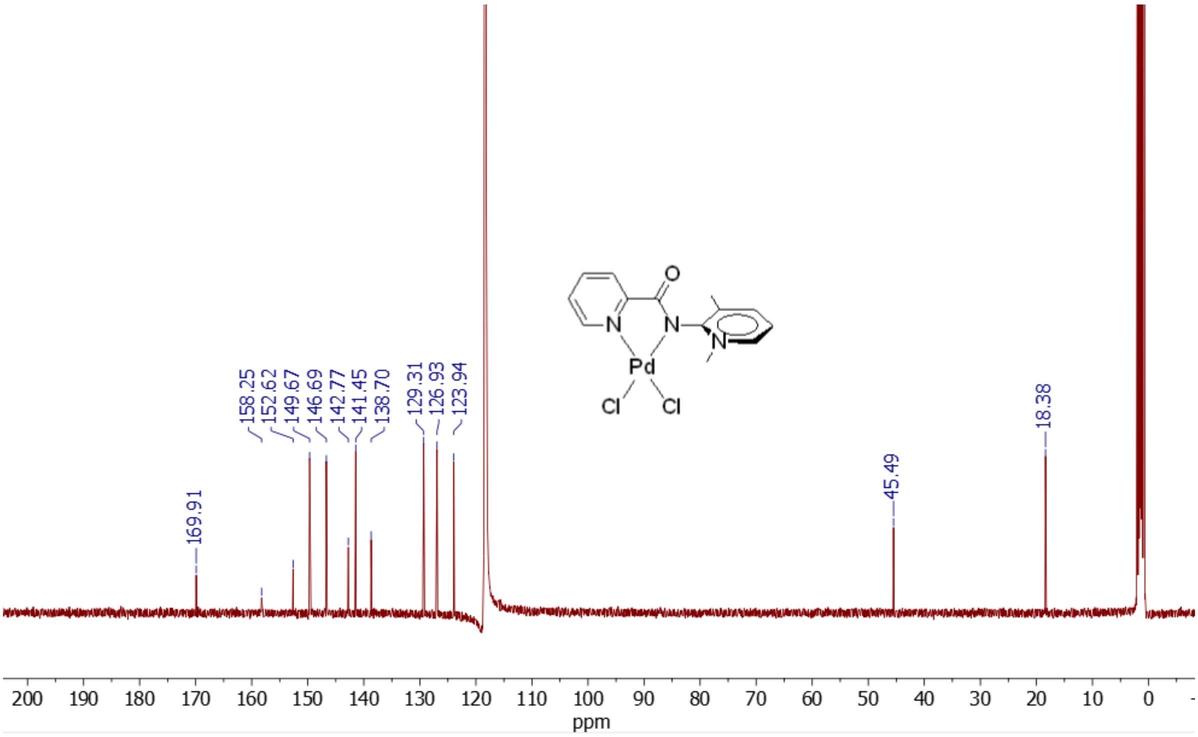

Figure S24. $^{13}C\{^{1}H\}$ NMR spectrum (CD_3CN, 298 K, 101 MHz) of **1**.

S.1.4.2 Synthesis of pyridyl-PYA Pd complex 2

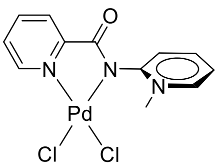

Following the general procedure from **L_2** (40 mg, 0.188 mmol) and [Pd(cod)Cl_2] (54 mg, 0.188 mmol). The product was further purified by microfiltration of a MeCN solution of the crude solid over Celite and subsequent precipitation upon Et_2O addition, yielding complex **2** as an orange solid (67 mg, 91%). Crystals suitable for X-ray diffraction were grown by slow diffusion of toluene in a DMSO solution of **2**. ^{1}H NMR (400 MHz, DMSO-d_6, 298 K): δ = 9.04 (dd, J_{HH} = 5.7, 1.5 Hz, 1H, CH_{pyr}), 8.88 (dd, J_{HH} = 6.3, 1.7 Hz, 1H, CH_{PYA}), 8.45 (td, $^3J_{HH}$ = 7.9, 1.5 Hz 1H, CH_{PYA}), 8.28 (td, $^3J_{HH}$ = 7.7, 1.5 Hz, 1H, CH_{pyr}), 7.94 – 7.87 (m, 2H, CH_{pyr} + CH_{PYA}), 8.16 (td, J_{HH} = 7.7, 1.5 Hz, 1H, CH_{pyr}), 7.86 – 7.78 (m, 2H, CH_{pyr} + CH_{PYA}), 4.24 (s, 3H, NCH_3) ppm. ^{13}C{^{1}H} NMR (101 MHz, , DMSO-d_6, 298 K): δ = 169.46 (*C*O), 157.40 (C_{PYA}), 151.24 (C_{pyr}), 148.45 (*C*H_{pyr}), 145.24 (*C*H_{PYA}), 144.36 (*C*H_{PYA}), 140.98 (*C*H_{pyr}), 128.68 (*C*H_{pyr} or *C*H_{PYA}), 128.48 (*C*H_{pyr} or *C*H_{PYA}), 126.10 (*C*H_{pyr}), 123.22 (*C*H_{PYA}), 43.78 (N*C*H_3) ppm. HR-MS (m/z): calculated for $C_{16}H_{19}ClN_3OPd$ [M–Cl+CH_3CN]$^+$ = 394.9891; found: 394.9878. Despite several attempts no satisfactory elemental analysis for **2** could be obtained. The closest match being $C_{12}H_{11}Cl_2N_3OPd$ · 0.5 $C_4H_{10}O$ (%): C 39.32, H 3.77, N 9.83; found: C 39.79, H 3.43, N 9.17.

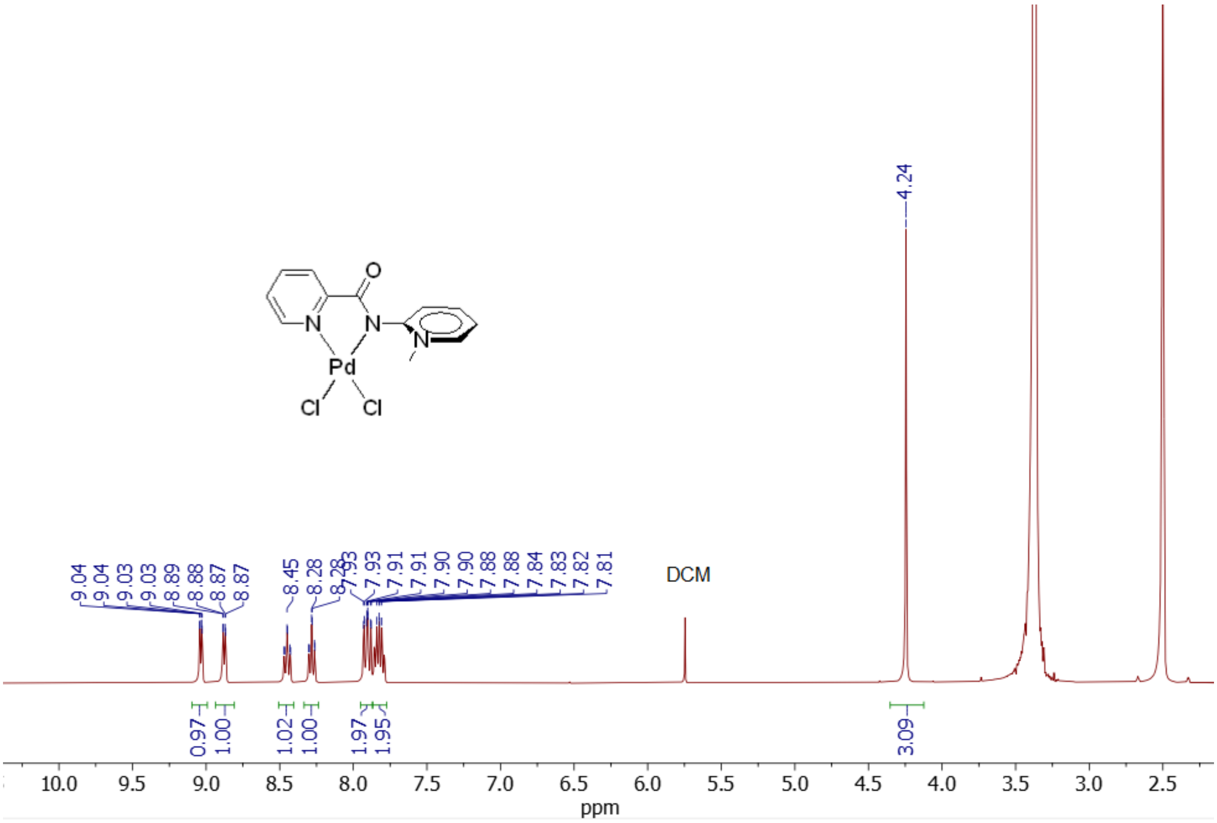

Figure S25. ^{1}H NMR spectrum (DMSO-d_6, 298 K, 400 MHz) of **2**.

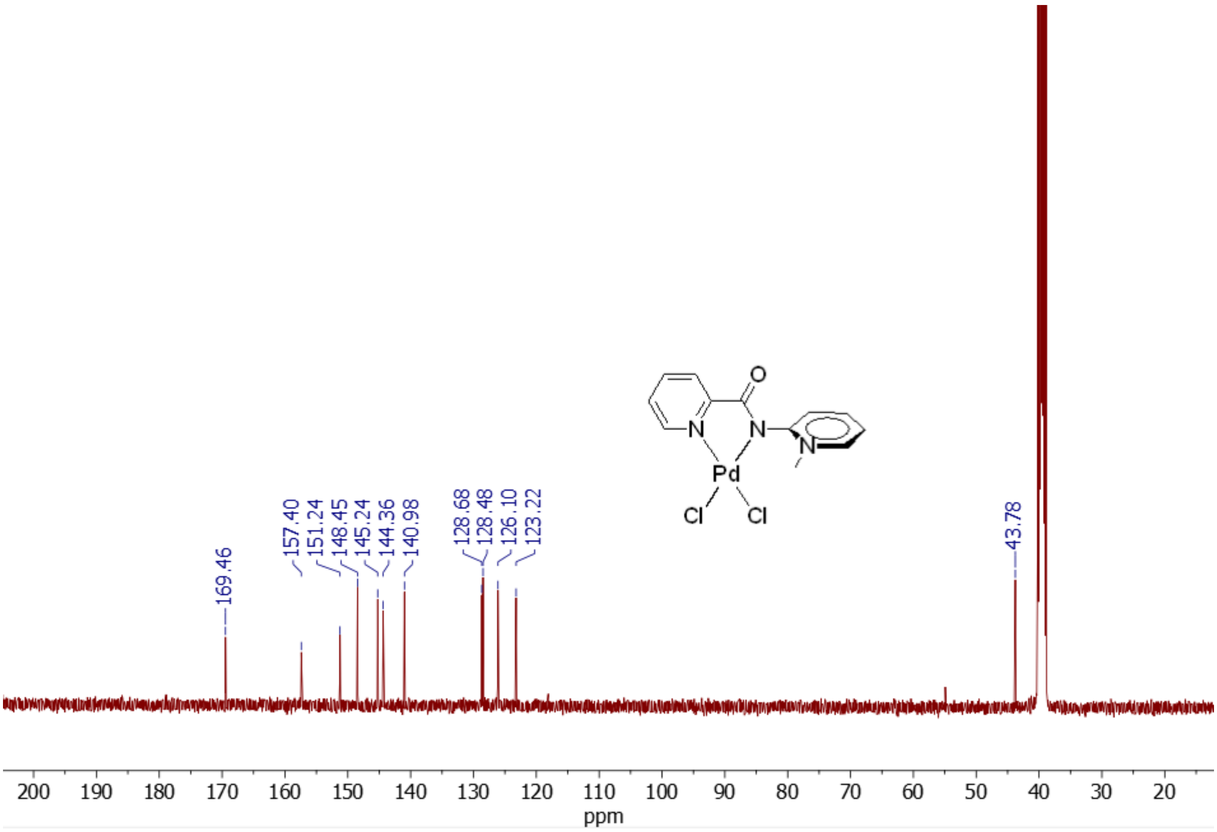

Figure S26. $^{13}C\{^1H\}$ NMR spectrum (DMSO-d_6, 298 K, 101 MHz) of **2**.

S.1.4.3 Synthesis of pyridyl-PYA Pd complex 3

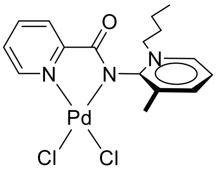

Following the general procedure, reaction of **L$_3$** (43 mg, 160 µmol) and [Pd(cod)Cl_2] (45 mg, 0.167 mmol) yielded complex **3** as a bright orange solid (60 mg, 80%). Crystals suitable for X-ray diffraction were grown by slow diffusion of Et_2O in a CH_2Cl_2 solution of **3**. ^1H NMR (400 MHz, CD_2Cl_2, 298 K): δ = 9.24 (d, J_{HH} = 5.7, 1.5 Hz, 1H, CH_{pyr}), 8.18 (dd, J_{HH} = 6.3, 1.6 Hz, 1H, CH_{PYA}), 8.14 (d, $^3J_{HH}$ = 7.6, 1H, CH_{PYA}), 8.08 (td, J_{HH} = 7.7, 1.5 Hz, 1H, CH_{pyr}), 7.93 (dd, J_{HH} = 7.7, 1.6 Hz, 1H, CH_{pyr}), 7.62 (ddd, J_{HH} = 7.7, 5.6, 1.6 Hz, 1H, CH_{pyr}), 7.55 (dd, $^3J_{HH}$ = 7.6, 6.3, 1H, CH_{PYA}), 5.40 (ddd, J_{HH} = 13.4, 9.1, 5.9 Hz, 1H, NCH_2), 4.20 (ddd, J_{HH} = 13.4, 9.1, 6.8 Hz, 1H, NCH_2), 2.56 (s, 3H, PYA-CCH_3), 2.01 – 1.81 (m, 2H, NCH_2CH_2), 1.41 (sextet, $^3J_{HH}$ = 7.4 Hz, 2H, CH_2CH_3), 0.92 (t, $^3J_{HH}$ = 7.4 Hz, 3H, CH_2CH_3). $^{13}C\{^1H\}$ NMR (101 MHz, CD_2Cl_2, 298 K): δ = 169.77 (*C*O), 157.95 (C_{PYA}), 151.83 (C_{pyr}), 149.54 (CH_{pyr}), 144.72 (CH_{PYA}), 140.26 (CH_{pyr}), 140.11 (CH_{PYA}), 139.15 (PYA-*C*CH_3), 129.48 (CH_{pyr}), 126.46 (CH_{pyr}), 123.05 (CH_{PYA}), 58.02 (NCH_2), 32.51 (NCH_2CH_3), 20.14 (CH_2CH_3), 18.68 (PYA-CH_3), 13.68 (CH_2CH_3) ppm. HR-MS (m/z): calculated for $C_{16}H_{19}ClN_3OPd$ $[M-Cl]^+$ = 410.0251; found: 410.0313. Elemental analysis calculated for $C_{16}H_{19}Cl_2N_3OPd \cdot 0.5\ CH_2Cl_2$ (%): C 40.52, H 4.12, N 8.59; found: C 40.26, H 4.05, N 8.51.

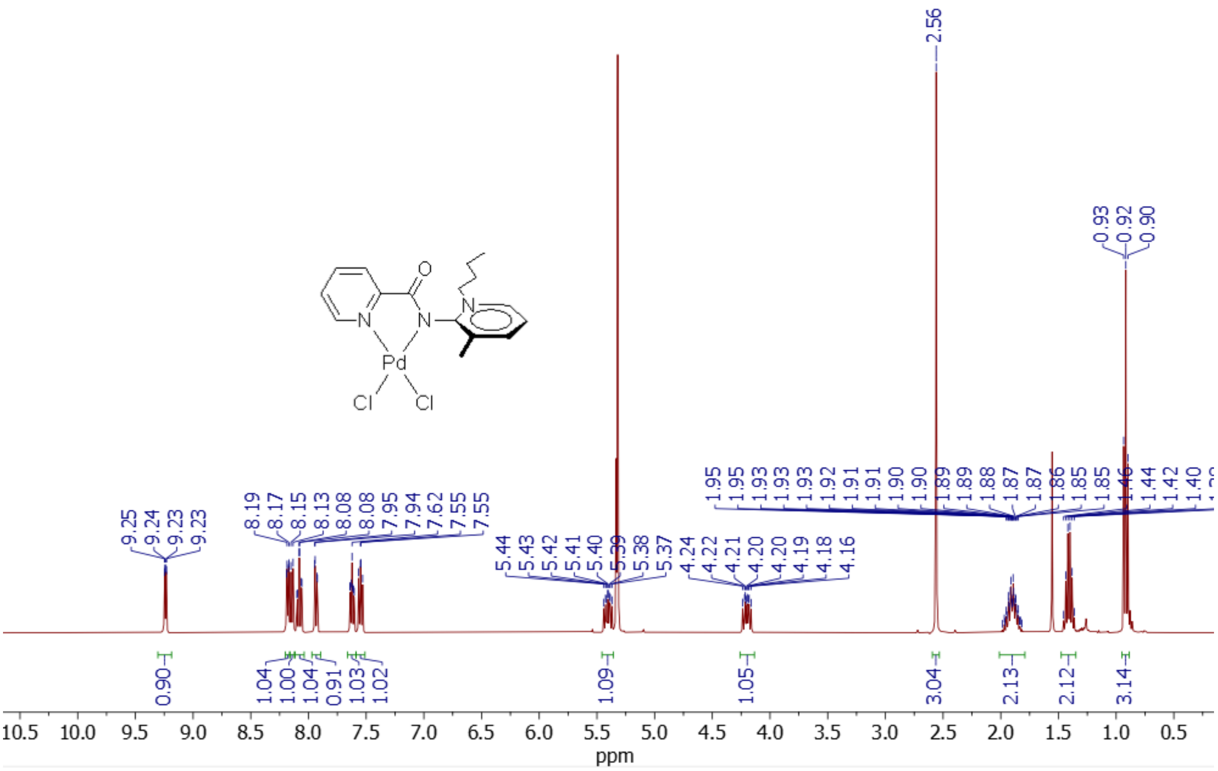

Figure S27. ^{1}H NMR spectrum (CD_2Cl_2, 298 K, 400 MHz) of **3**.

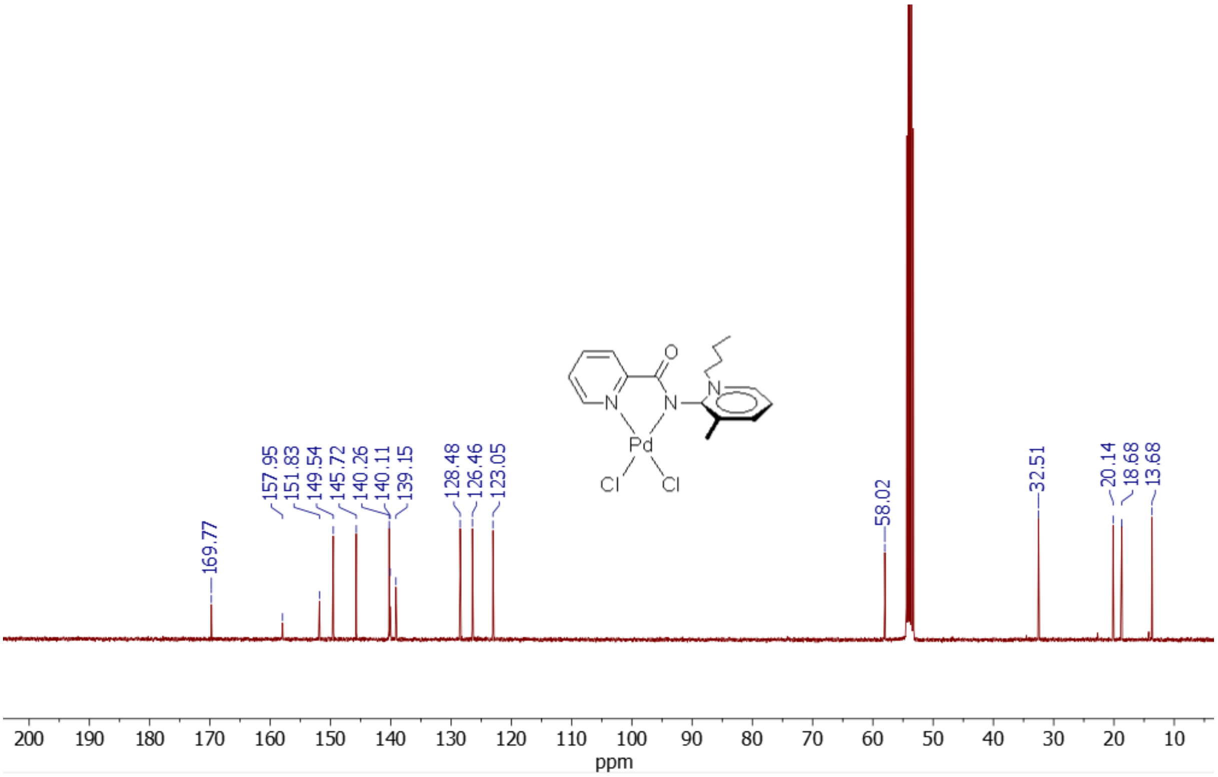

Figure S28. $^{13}C\{^{1}H\}$ NMR spectrum (CD_2Cl_2, 298 K, 101 MHz) of **3**.

S.1.4.4 Synthesis of pyridyl-PYA Pd complex 4

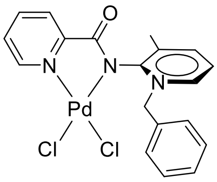

Following the general procedure, reaction of **L_4** (100 mg, 330 μmol) and [Pd(cod)Cl_2] (87 mg, 300 mmol) yielded complex **4** as a yellow solid (120 mg, 83%). ^{1}H NMR (400 MHz, CD_2Cl_2, 298 K): δ = 9.24 (d, $^3J_{HH}$ = 5.5 Hz, 1H, CH_{pyr}), 8.15 (d, $^3J_{HH}$ = 7.5 Hz, 1H, CH_{PYA}), 8.14 (d, $^3J_{HH}$ = 7.6, 1H, CH_{pyr}), 8.09 – 8.02 (m, 2H, CH_{pyr} + CH_{PYA}), 7.85 (d, $^3J_{HH}$ = 7.8 Hz, 1H, CH_{pyr}), 7.62 (dd, $^3J_{HH}$ = 7.7, 5.5 Hz, 1H, CH_{pyr}), 7.50 (t, $^3J_{HH}$ = 7.6 Hz, 1H, CH_{PYA}), 7.43 – 7.21 (m, 5H, CH_{benzyl}), 6.21 (d, $^2J_{HH}$ = 14.8 Hz, 1H, CH_2), 5.81 (d, $^2J_{HH}$ = 14.8 Hz, 1H, CH_2), 2.58 (s, 3H, PYA-CCH_3) ppm.). ^{13}C{^{1}H} NMR (101 MHz, CD_2Cl_2 , 298 K): δ = 169.40 (*C*O), 158.22 (*C*$_{PYA}$), 151.73 (*C*$_{pyr}$), 149.49 (*C*H_{pyr}), 145.92 (*C*H_{PYA}), 140.24 (*C*H_{pyr}), 139.87 (*C*H_{PYA}), 138.95 (PYA-*C*CH_3), 132.89 (*C*$_{benzyl}$), 130.06 (2C, *C*H_{benzyl}), 129.79 (3C, *C*H_{benzyl}), 128.45 (*C*H_{pyr}), 126.41 (*C*H_{pyr}), 123.02 (*C*H_{PYA}), 60.14 (*C*H_2), 18.71 (PYA-*C*H_3) ppm. HR-MS (m/z): calculated for $C_{19}H_{17}ClN_3OPd$ [M-Cl]$^+$ = 444.0095; found: 444.0073. Elemental analysis calculated for $C_{19}H_{17}Cl_2N_3OPd$ · 0.25 CH_2Cl_2 (%): C 46.07, H 3.51, N 8.37; found: C 46.05, H 3.66, N 8.06.

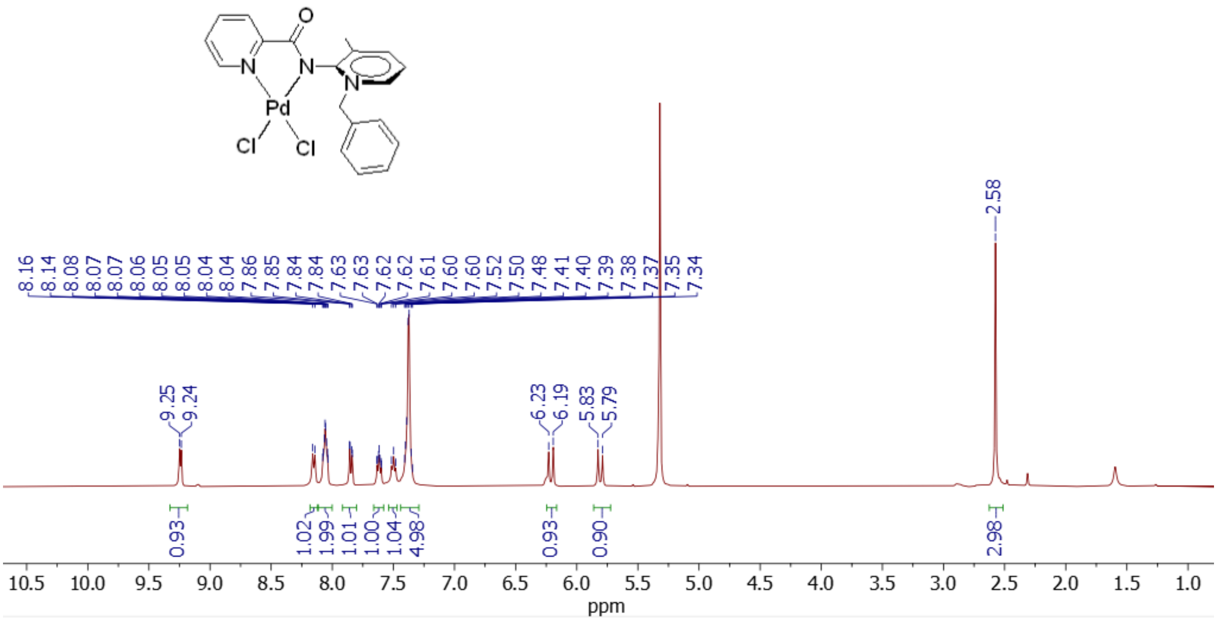

Figure S29. ^{1}H NMR spectrum (CD_2Cl_2, 298 K, 400 MHz) of **4**.

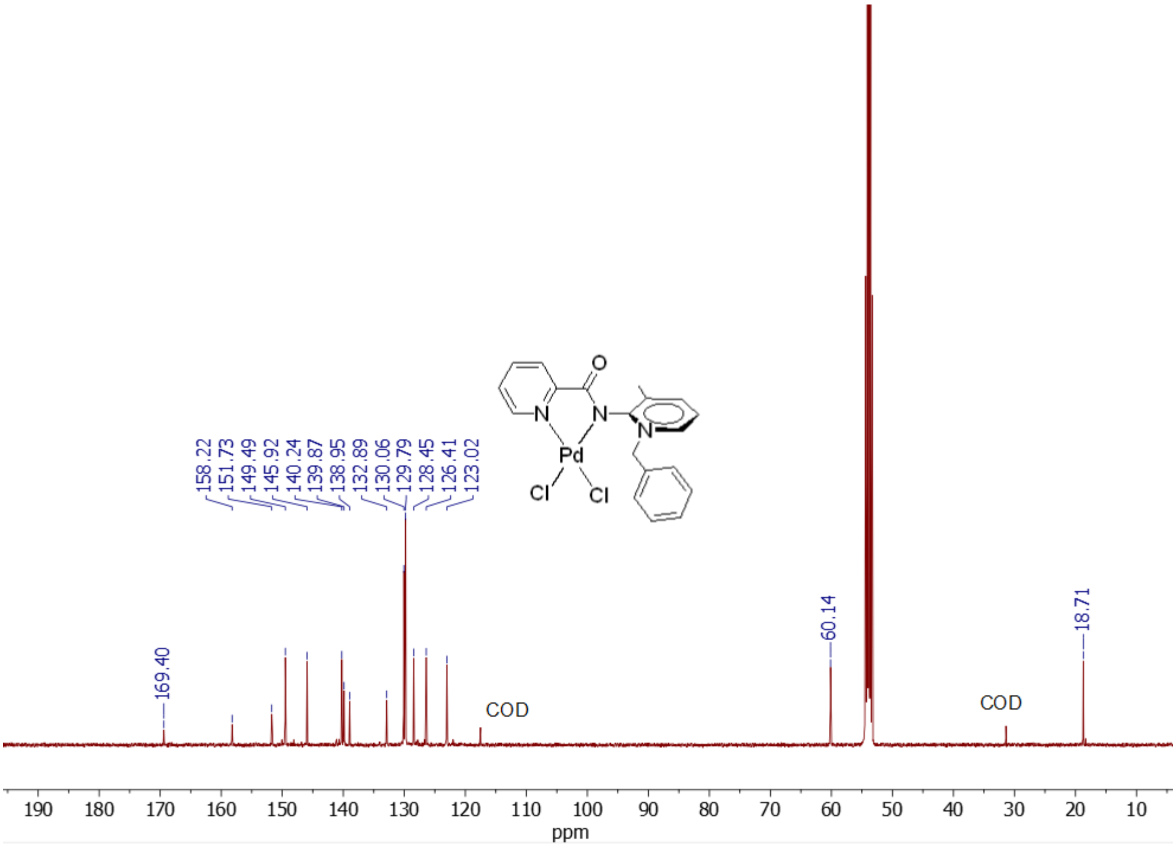

Figure S30. $^{13}C\{^1H\}$ NMR spectrum (CD_2Cl_2, 298 K, 101 MHz) of **4**.

S.1.4.5 Synthesis of pyridyl-PYA Pd complex 5

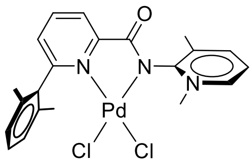

Following the general procedure, reaction of **L$_5$** (39 mg, 0.117 mmol) and [Pd(cod)Cl_2] (33 mg, 0.117 mmol) yielded complex **5** as a dark orange solid (57 mg, 95%). Crystals suitable for X-ray diffraction analysis were grown by diffusion of n-heptane into a CH_2Cl_2 solution of **5**. ^{1}H NMR (400 MHz, CD_2Cl_2, 298 K): δ = 8.17 – 8.10 (m, 2H, 2 CH_{PYA}), 8.09 – 8.01 (m, 2H, 2 CH_{pyr}), 7.50 (dd, $^3J_{HH}$ = 7.7, 6.2 Hz, 1H, CH_{PYA}), 7.40 (dd, $^3J_{HH}$ = 7.4, 2.4 Hz, 1H, CH_{pyr}), 7.25 (t, $^3J_{HH}$ = 7.5 Hz, 1H, CH_{xyl}), 7.10 (d, $^3J_{HH}$ = 7.5 Hz, 1H, CH_{xyl}), 4.35 (s, 3H, NCH_3), 2.62 (s, 3H, PYA-CCH_3), 2.33 (d, 6H, xyl-CCH_3). $^{13}C\{^1H\}$ NMR (101 MHz, CD_2Cl_2 , 298 K): δ = 168.89 (*C*O), 163.65 (C_{pyr}), 158.32 (C_{PYA}), 153.94 (C_{pyr}-xyl), 145.75 (CH_{PYA}), 140.76 (CH_{PYA}), 139.76 (CH_{pyr}), 139.46 (PYA-*C*CH$_3$), 139.12 (C_{xyl}-pyr), 136.60 (xyl-*C*CH$_3$), 136.45 (xyl-*C*CH$_3$), 132.65 (CH_{pyr}), 129.30 (CH_{xyl}), 127.76 (CH_{xyl}), 127.69 (CH_{xyl}), 125.08 (CH_{pyr}), 122.69 (CH_{PYA}), 45.59 (N*C*H$_3$), 21.33 (xyl-*C*H$_3$), 21.27(xyl-*C*H$_3$), 19.00 (PYA-*C*H$_3$) ppm. HR-MS (m/z): calculated for $C_{21}H_{21}ClN_3OPd$ [M–Cl]$^+$ = 472.0408; found: 472.0410. Elemental analysis calculated for $C_{21}H_{21}Cl_2N_3OPd \cdot 0.75\ CH_2Cl_2$ (%): C 45.64, H 3.96, N 7.34; found: C 45.65, H 3.87, N 7.10.

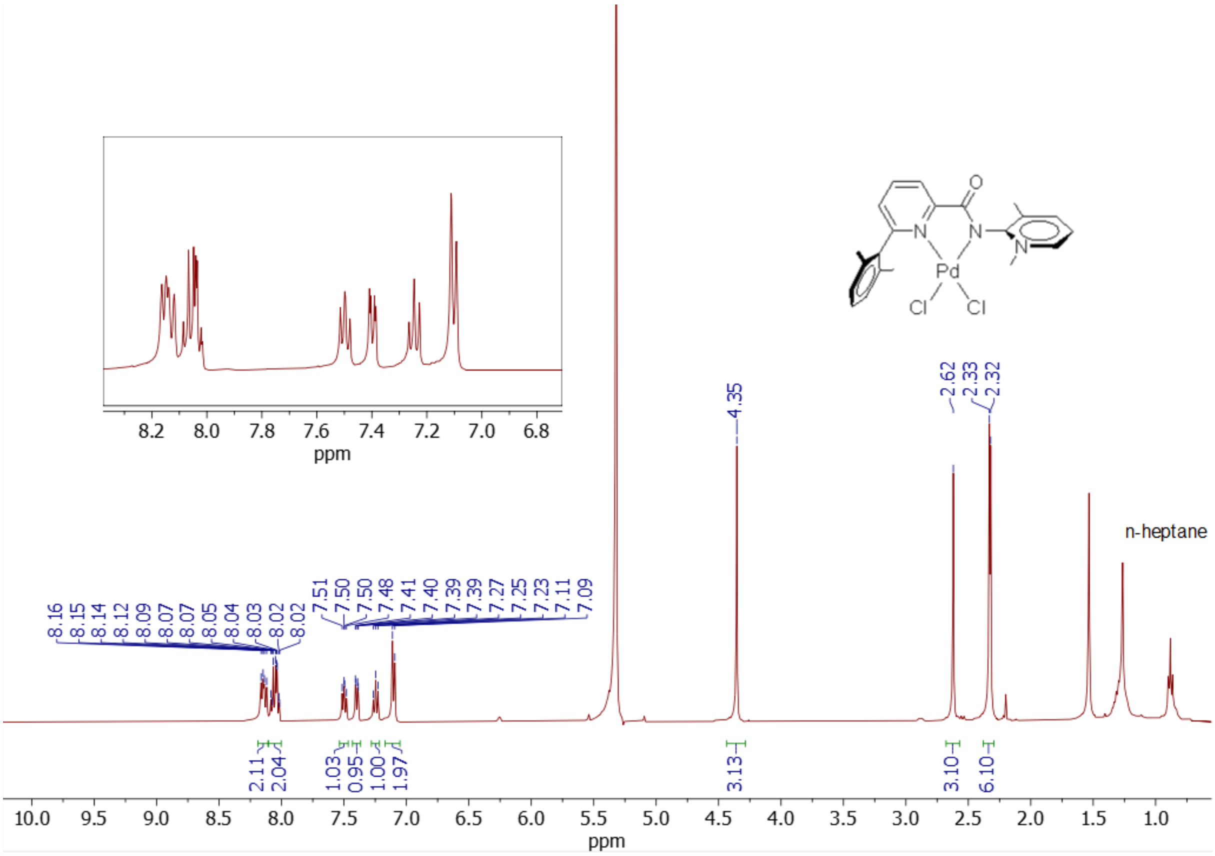

Figure S31. ^{1}H NMR spectrum (CD_2Cl_2, 298 K, 400 MHz) of **5**.

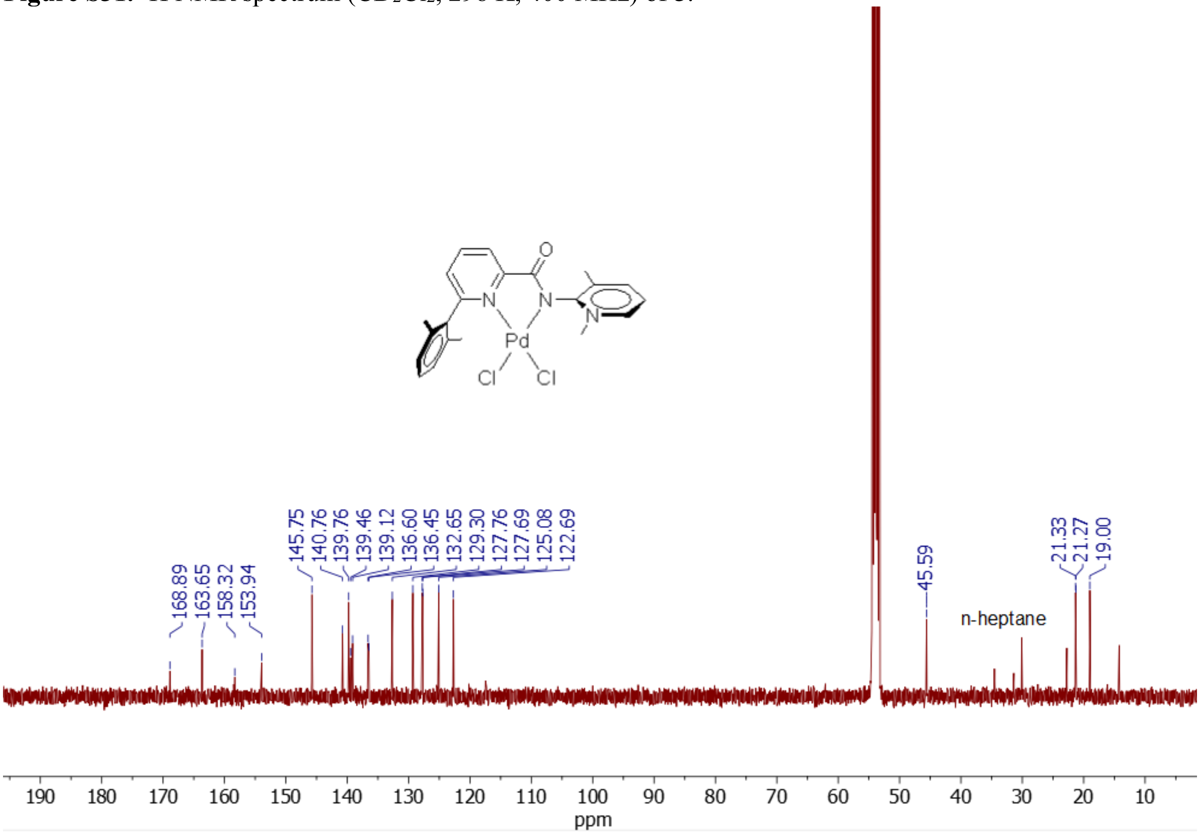

Figure S32. $^{13}C\{^{1}H\}$ NMR spectrum (CD_2Cl_2, 298 K, 101 MHz) of **5**.

S.1.4.6 Synthesis of pyridyl-PYA Pd complex 7

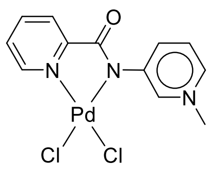

[L_7H]I (200 mg, 0.57 mmol) and NH_4PF_6 (465 mg, 2.85 mmol) were separately dissolved in a minimal amount of H_2O, combined, and the resulting suspension was stirred at 23 °C for 10 min. The suspension was filtered and and the filtrate washed with H_2O and Et_2O. The residue was dried under vacuum to yield **[L_7H]PF_6** as a colourless solid (195 mg, 95%). **[L_7H]PF_6** (180 mg, 0.5 mmol), [Pd(cod)Cl_2] (143 mg, 0.5 mmol) and Cs_2CO_3 (490 mg, 1.5 mmol) were suspended in CH_2Cl_2 (20 mL) and stirred at 23 °C for 16 h. The resulting orange solution was filtered and washed with CH_2Cl_2 until washings were clear. The combined filtrates were concentrated, triturated into Et_2O, and the residue was collected and dried under vacuum to yield **7** as an orange solid (146 mg, 75%). ^{1}H NMR (400 MHz, CD_3CN, 298 K): δ = 9.16 (d, J_{HH} = 5.6, 1.5 Hz, 1H, CH_{pyr}), 8.70 (t, $^4J_{HH}$ = 1.5 Hz, 1H, CH_{PYA}), 8.42 (dt, J_{HH} = 8.2, 1.5 Hz, 1H, CH_{PYA}), 8.20 (d, $^3J_{HH}$ = 6.1 Hz, 1H, CH_{PYA}), 8.10 (td, J_{HH} = 7.7, 1.5 Hz, 1H, CH_{pyr}), 7.88 (dd, J_{HH} = 7.7, 1.6 Hz, 1H, CH_{pyr}), 7.78 (dd, J_{HH} = 8.2, 6.1 Hz, 1H, CH_{PYA}), 7.61 (ddd, J_{HH} = 7.7, 5.6, 1.6 Hz, 1H, CH_{pyr}), 4.24 (s, 3H, NCH_3) ppm. ^{13}C{^{1}H} NMR (101 MHz, CD_3CN, 298 K): δ = 171.72 (*C*O), 154.37 (C_{pyr}), 149.40 (*C*H_{pyr}), 147.51 (C_{PYA}), 145.98 (*C*H_{PYA}), 144.79 (*C*H_{PYA}), 141.21 (*C*H_{pyr}), 139.35 (*C*H_{PYA}), 128.63 (*C*H_{pyr}), 126.77 (*C*$H_{pyr\ or}$ CH_{PYA}), 126.66 (*C*$H_{pyr\ or}$ CH_{PYA}), 48.86 (N*C*H_3) ppm. HR-MS (m/z): calculated for $C_{12}H_{11}ClN_3OPd$ $[M–Cl]^+$ = 353.9625; found: 353.9619. Elemental analysis calculated for $C_{12}H_{11}Cl_2N_3OPd \cdot 0.5\ CH_2Cl_2$ (%): C 34.67, H 2.79, N 9.70; found: C 35.09, H 2.91, N 9.40.

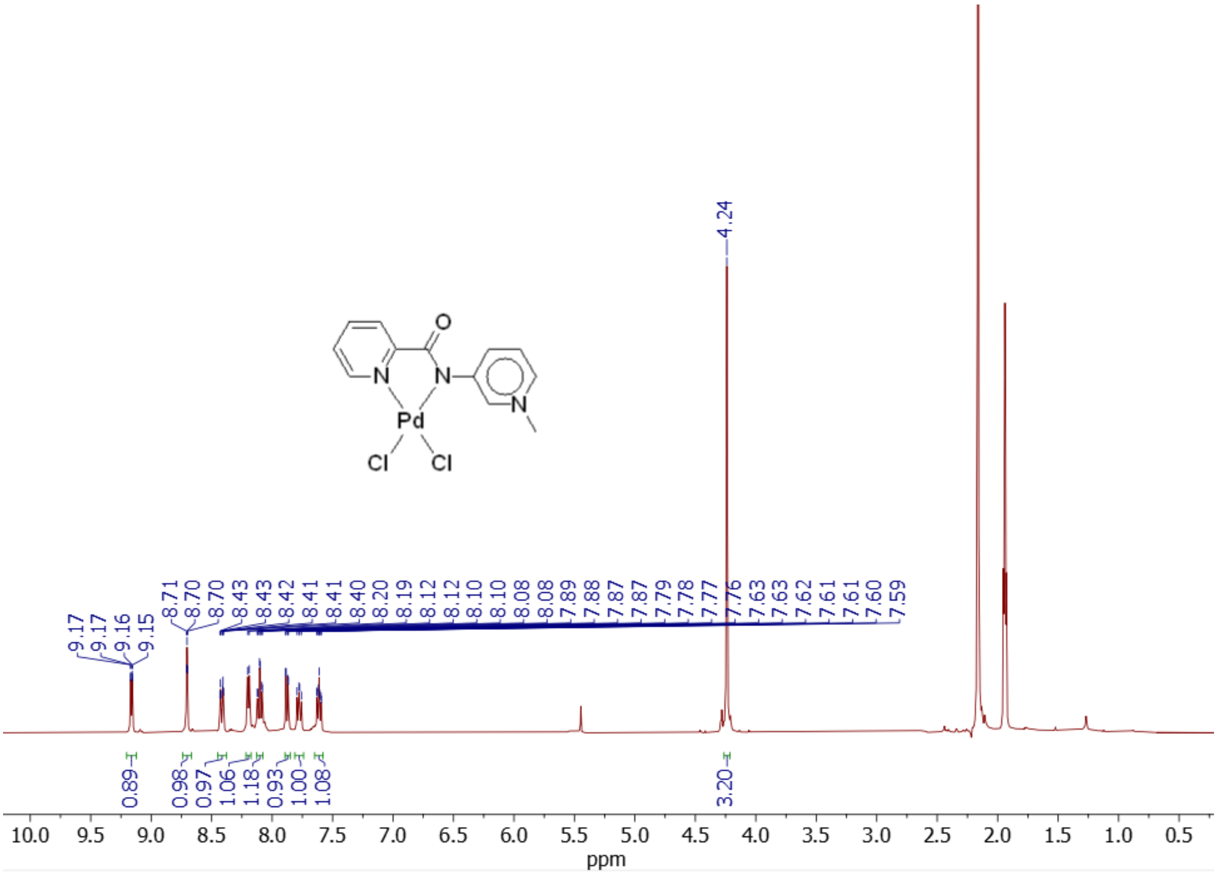

Figure S33. ^{1}H NMR spectrum (CD_3CN, 298 K, 400 MHz) of **7**.

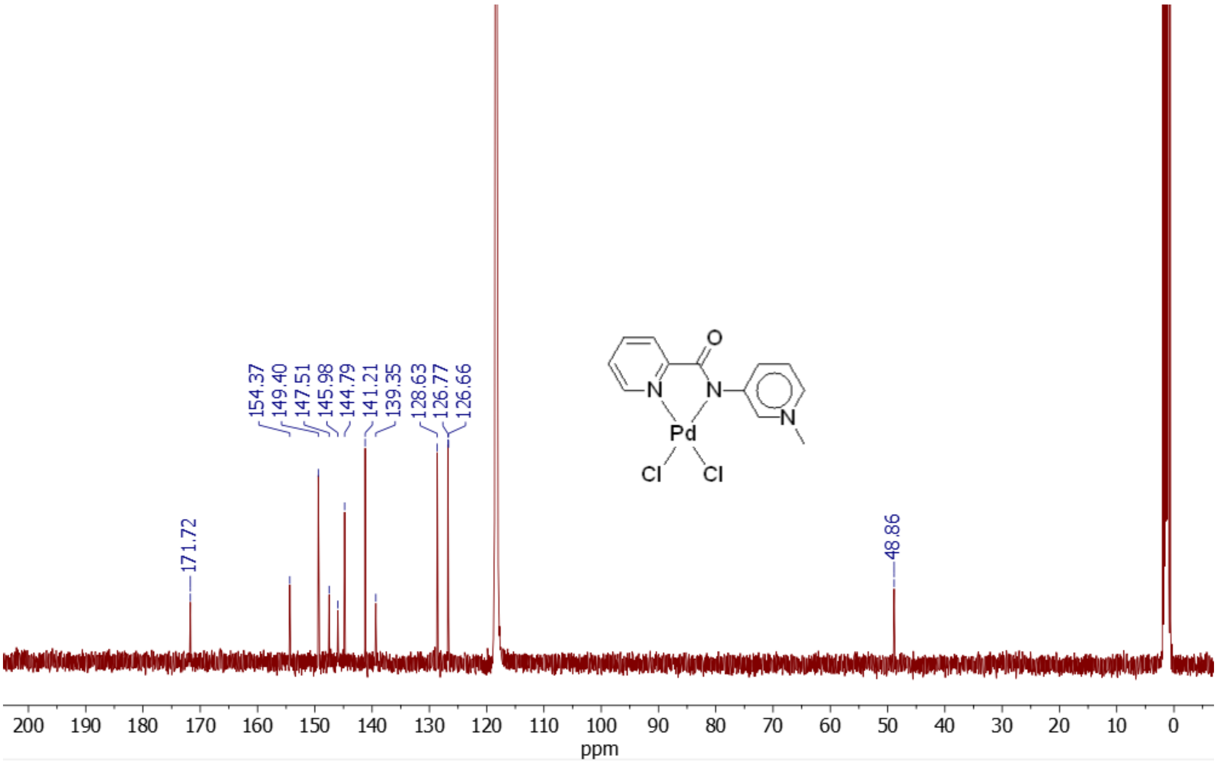

Figure S34. $^{13}C\{^{1}H\}$ NMR spectrum (CD_3CN, 298 K, 101 MHz) of **7**.

S.1.4.8 Synthesis of pyridyl-PYA Pd complex 8

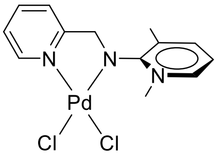

In a N_2 atmosphere, [Pd(cod)Cl_2] (86 mg, 0.3 mmol) was added a solution of $\mathbf{L_8}$ (64 mg, 0.3 mmol) in MeCN (2 mL) and the resulting bright red suspension was stirred for 1 h at 50 °C. The solvent was then evaporated, the residue was dissolved in CH_2Cl_2 (1 mL) and precipitated with Et_2O (5 mL). The precipitate was filtered, washed with more Et_2O and dried under vacuum to yield **8** as a bright red solid (82 mg, 70%). Crystals suitable for X-ray diffraction analysis were grown by slow diffusion of Et_2O into a 1:1 MeCN/ CH_2Cl_2 solution of **8**. ^{1}H NMR (400 MHz, CD_2Cl_2, 298 K): δ = 8.94 (d, J_{HH} = 5.6, 1.5 Hz, 1H, CH_{pyr}), 7.84 (td, J_{HH} = 7.7, 1.5 Hz, 1H, CH_{pyr}), 7.61 (dd, J_{HH} = 6.6, 1.8 Hz 1H, CH_{PYE}), 7.50 (d, $^3J_{HH}$ = 7.3 Hz, 1H, CH_{PYE}), 7.35 – 7.29 (m, 2H, CH_{pyr}), 6.75 (t, $^3J_{HH}$ = 7.0 Hz 1H, CH_{PYE}), 4.35 (s, 3H, NCH_3), 2.59 (s, 3H, PYE-CCH_3) ppm. Methylene protons not resolved. At 213 K, they appear as a pair of doublets (δ = 5.64 and 4.21 ppm; $^2J_{HH}$ = 15.6 Hz) and at 328 K as a broad singlet (δ = 4.92 ppm; Fig. S36). ^{13}C{^{1}H} NMR (101 MHz, CD_2Cl_2, 298 K): δ = 165.08 (C_{PYE}), 154.55 (C_{pyr}), 150.71 (CH_{pyr}), 142.98 (CH_{PYE}), 139.81 (CH_{PYE}), 139.18 (CH_{pyr}), 133.73 (PYE-CCH$_3$), 123.31 (CH_{pyr}), 119.94 (CH_{pyr}), 113.81 (CH_{PYE}), 63.09 (CH_2), 45.92 (NCH_3), 21.93 (PYE-CH_3) ppm. HR-MS (m/z): calculated for $C_{13}H_{15}Cl_2N_3NaPd$ $[M+Na]^+$ = 411.9576; found: 411.9566. Despite several attempts, no satisfactory elemental analysis data could be obtained.

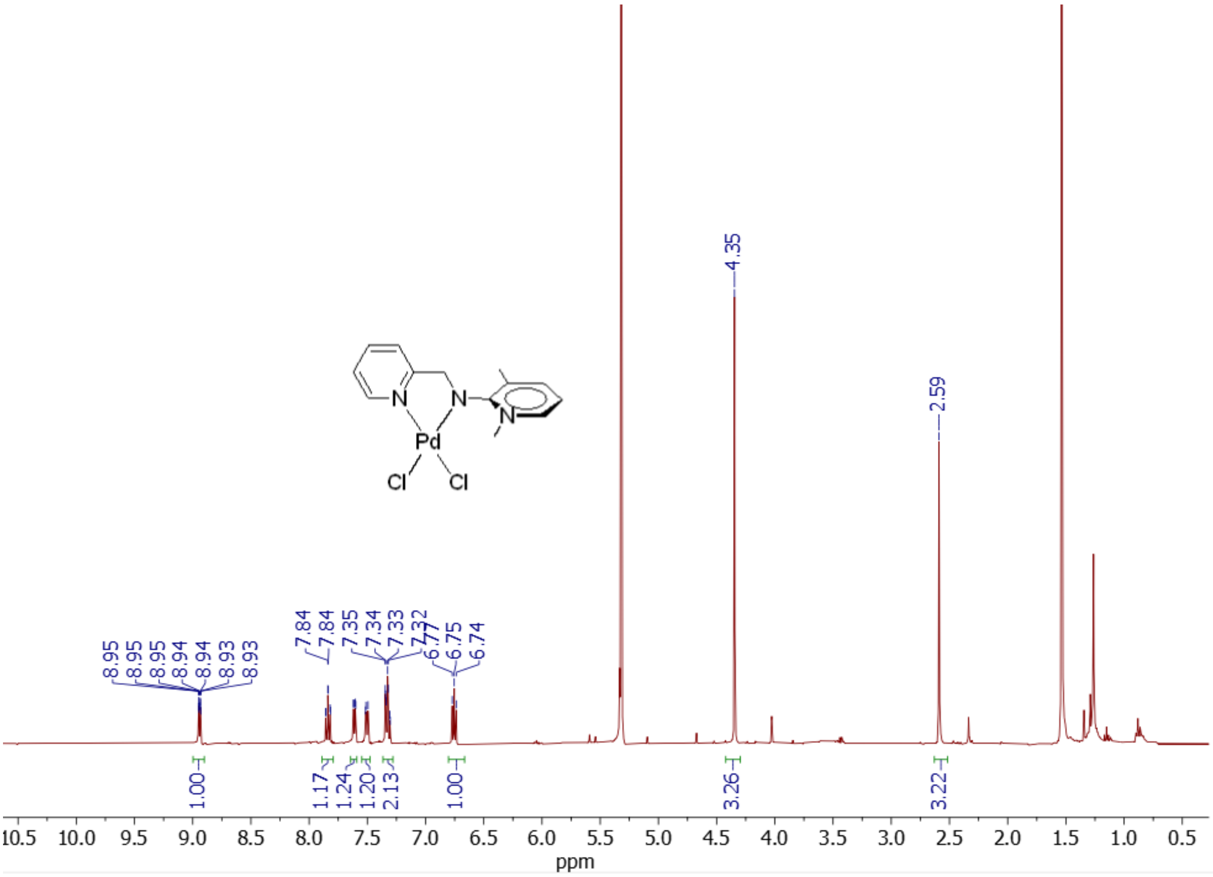

Figure S35. ^{1}H NMR spectrum (CD_2Cl_2, 298 K, 400 MHz) of **8**.

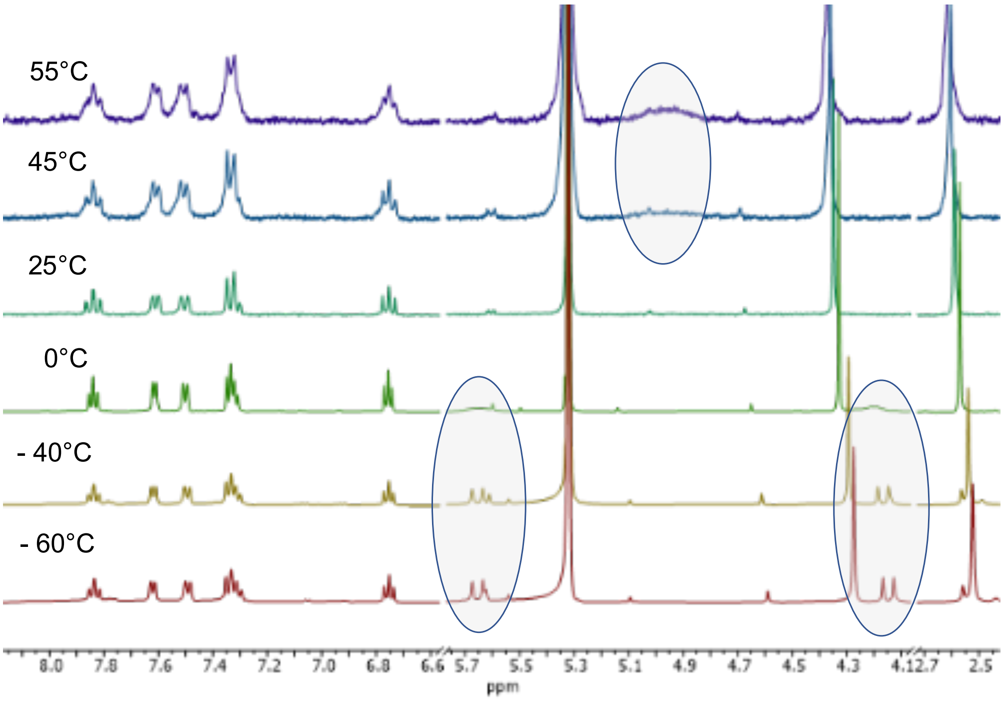

Figure S36. Variable temperature ^{1}H NMR spectra of complex **8** (CD_2Cl_2, 213 - 328 K, 400 MHz), indicating the coalescence of the AB doublets around 4.2 and 5.6 ppm at 25 °C to a singlet at 5 ppm.

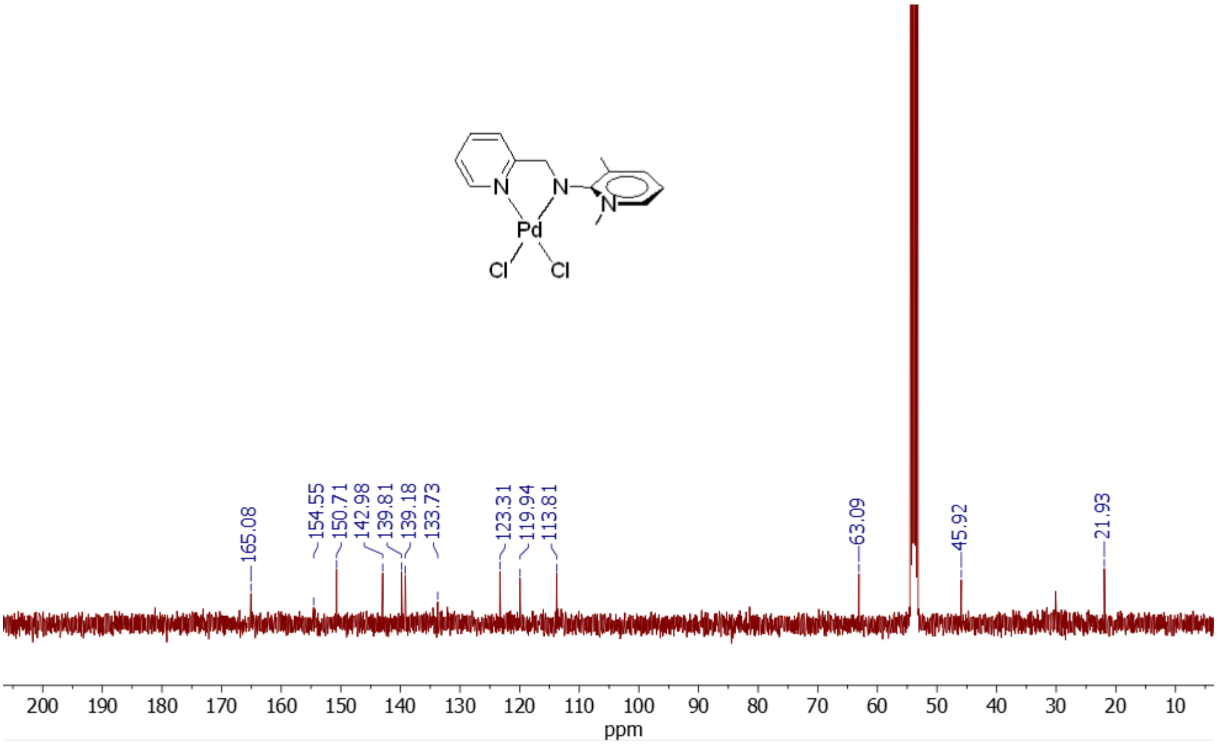

Figure S37. $^{13}C\{^1H\}$ NMR spectrum (CD_2Cl_2, 298 K, 101 MHz) of **8**.

S.2. Crystal structure determinations

Crystals of **[L_1H]PF_6, L_1, 1**, **2**, **3, 5** and **8** immersed in parabar oil were mounted at ambient conditions and transferred into the stream of nitrogen (173 K). Measurements for **[L_1H]PF_6, L_1, 1**, **2** and **8** were made on a RIGAKU Synergy S area-detector diffractometer1 using mirror optics monochromated Cu Kα radiation (λ = 1.54184 Å). Measurements for **3** and **5** were made on an *Oxford Diffraction SuperNova* area-detector diffractometer using mirror optics monochromated Mo *K*α radiation (λ = 0.71073 Å) and Al filtered.

Data reduction was performed using the *CrysAlisPro* [S3] program. The intensities were corrected for Lorentz and polarization effects, and an absorption correction based on the multi-scan method using SCALE3 ABSPACK in *CrysAlisPro* [S3] was applied.

All the structures were solved by intrinsic phasing using *SHELXT* [S4], which revealed the positions of all non-hydrogen atoms of the title compound. All non-hydrogen atoms were refined anisotropically. For **[L_1H]PF_6, L_1, 5** H-atoms were located from the difference density map and had their positions and isotropic displacement parameters refined freely. For **1, 3** and **8** H-atoms were assigned in geometrically calculated positions and refined using a riding model where each H-atom was assigned a fixed isotropic displacement parameter with a value equal to 1.2Ueq of its parent atom (1.5Ueq for methyl groups). For **2,** H-atoms were located from the difference density map and had their positions and isotropic displacement parameters refined freely, except for H2 and H5 (CIF labelling), where the atoms were refined using a riding model where each H-atom was assigned a fixed isotropic displacement parameter with a value equal to 1.2Ueq of its parent atom. In addition, anharmonic motion was refined for Pd1.

Refinements were carried out on F^2 using full-matrix least-squares procedures, which minimized the function $\Sigma w(F_o^2 - F_c^2)^2$. The weighting scheme was based on counting statistics and included a factor to downweight the intense reflections. All calculations were performed using the *SHELXL-2014/7* [S5] program in OLEX2.[S6]

A disorder solvent could not be modeled in **1**, therefore it is not included in the model and instead a mask was used to calculate the electron density present in the void area and its contribution was included into the calculated structure factors (total electron count 63e).

The structure of **3** was twinned by a polymorph (monoclinic crystal system). The multi crystal separation during the data reduction led to an overall 66% completeness of the data only due to reflection overlap. Since the unit cells differ there is no twin law that can be use in the refinement and therefore the refinement had to proceed using the major component polymorph (orthorhombic crystal system) against the reflections containing the overlap, which leads to artificial residual densities around the pseudo translated Pd position on the second polymorph (about 0.25 along the b unit cell axis). In addition, the structure is refined as an inversion twin.

For the structure of **5**, a disorder model was used for part of the structure where the occupancies of each disorder component was refined through the use of a free variable. The sum of equivalent components was constrained to 1 (100%). Final occupancies are listed in the associated crystallographic data.

For **8**, twinning was detected on the frames where the second component corresponded to a twofold rotation around [-0.26 -0.96 0.04] (reciprocal) or [-0.48 -0.88 -0.07] (direct). The refinement proceeded against reflections of the major component only.

Data collection and refinement parameters for **[L$_1$H]PF$_6$**, **L$_1$**, **1**, **2**, **3, 5** and **8** are given in Tables S1–S4. Crystallographic data for all structures reported in this paper have been deposited with the Cambridge Crystallographic Data Centre (CCDC) as supplementary publication numbers 2115311–2115316.

Table S1. Crystal data and structure refinement for **[L$_1$H]PF$_6$** and **L$_1$**.

CCDC No.	2297421	2297422
Empirical formula	$C_{13}H_{14}F_6N_3OP$	$C_{13}H_{13}N_3O$
Formula weight	373.24	227.26
Temperature/K	173.01(10)	173.01(10)
Crystal system	monoclinic	monoclinic
Space group	P2$_1$/n	P2$_1$/c
a/Å	21.02657(6)	10.25664(8)
b/Å	14.43623(4)	9.94244(8)
c/Å	21.17731(6)	11.26378(9)
α/°	90	90
β/°	90.6664(2)	91.5286(7)
γ/°	90	90
Volume/Å^3	6427.82(3)	1148.226(15)
Z	16	4
ρ_{calc}g/cm^3	1.543	1.315
μ/mm^{-1}	2.208	0.697
F(000)	3040.0	480.0
Crystal size/mm^3	0.432 × 0.338 × 0.207	0.155 × 0.13 × 0.075
Radiation	Cu Kα (λ = 1.54184)	Cu Kα (λ = 1.54184)
2Θ range for data collection/°	5.89 to 147.988	8.624 to 140.468
Index ranges	$-26 \le h \le 26$, $-18 \le k \le 18$, $-26 \le l \le 26$	$-12 \le h \le 12$, $-12 \le k \le 12$, $-13 \le l \le 13$
Reflections collected	246423	22145
Independent reflections	13039 [R_{int} = 0.0341 R_{sigma} = 0.0102]	2186 [R_{int} = 0.0303, R_{sigma} = 0.0123]
Data/restraints/parameters	13039/0/1090	2186/0/207
Goodness-of-fit on F^2	1.050	1.042
Final R indexes [I>=2σ (I)]	R_1 = 0.0424, wR_2 = 0.1161	R_1 = 0.0312, wR_2 = 0.0806
Final R indexes [all data]	R_1 = 0.0434, wR_2 = 0.1168	R_1 = 0.0320, wR_2 = 0.0813
Largest diff. peak/hole / e Å^{-3}	0.73/-0.39	0.21/-0.16

Table S2. Crystal data and structure refinement for complexes **1** and **2**.

Identification code	2297423	2297424
Empirical formula	$C_{13}H_{13}Cl_2N_3OPd$	$C_{12}H_{11}Cl_2N_3OPd$
Formula weight	404.56	390.561
Temperature/K	173.01(10)	173.00(10)
Crystal system	orthorhombic	monoclinic
Space group	Pbca	$P2_1/n$
a/Å	13.80951(16)	10.44149(12)
b/Å	14.64287(17)	11.38265(13)
c/Å	20.4888(2)	11.56249(13)
α/°	90	90
β/°	90	90.8439(10)
γ/°	90	90
Volume/Å^3	4143.06(8)	1374.07(3)
Z	8	4
ρ_{calc}g/cm^3	1.297	1.888
μ/mm^{-1}	9.593	14.435
F(000)	1600.0	768.0
Crystal size/mm^3	0.254 × 0.091 × 0.059	0.214 × 0.18 × 0.113
Radiation	Cu Kα (λ = 1.54184)	Cu Kα (λ = 1.54184)
2Θ range for data collection/°	9.806 to 136.152	10.9 to 152.88
Index ranges	$-16 \le h \le 16$, $-17 \le k \le 17$, $-22 \le l \le 24$	$-12 \le h \le 13$, $-14 \le k \le 14$, $-14 \le l \le 14$
Reflections collected	30476	27616
Independent reflections	3774 [R_{int} = 0.0572, R_{sigma} = 0.0252]	2890 [R_{int} = 0.0692, R_{sigma} = 0.0235]
Data/restraints/parameters	3774/0/183	2890/0/234
Goodness-of-fit on F^2	1.095	1.045
Final R indexes [I>=2σ (I)]	R_1 = 0.0394, wR_2 = 0.1222	R_1 = 0.0291, wR_2 = 0.0747
Final R indexes [all data]	R_1 = 0.0425, wR_2 = 0.1250	R_1 = 0.0292, wR_2 = 0.0747
Largest diff. peak/hole / e Å^{-3}	1.33/-0.77	0.62/-0.80

Table S3. Crystal data and structure refinement for **3** and **5**.

Identification code	2297425	2297426
Empirical formula	$C_{17}H_{21}Cl_4N_3OPd$	$C_{21}H_{21}Cl_2N_3OPd$
Formula weight	531.57	508.71
Temperature/K	173.00(10)	173.01(10)
Crystal system	orthorhombic	monoclinic
Space group	$Pca2_1$	C2/c
a/Å	19.4911(2)	30.25121(18)
b/Å	9.94886(11)	9.45051(5)
c/Å	21.9647(3)	14.29074(7)
α/°	90	90
β/°	90	93.7911(5)
γ/°	90	90
Volume/Å^3	4259.27(9)	4076.63(4)
Z	8	8
ρ_{calc}g/cm^3	1.658	1.658

μ/mm^{-1}	1.366	1.190
F(000)	2128.0	2048.0
Crystal size/mm^3	0.19 × 0.142 × 0.086	0.218 × 0.132 × 0.073
Radiation	Mo Kα (λ = 0.71073)	Mo Kα (λ = 0.71073)
2Θ range for data collection/°	4.094 to 60.82	4.516 to 59.148
Index ranges	-27 ≤ h ≤ 27, -14 ≤ k ≤ 14, -31 ≤ l ≤ 31	-42 ≤ h ≤ 42, -13 ≤ k ≤ 13, -19 ≤ l ≤ 19
Reflections collected	62815	55339
Independent reflections	12785 [R_{int} = 0.0391, R_{sigma} = 0.0305]	5712 [R_{int} = 0.0342, R_{sigma} = 0.0165]
Data/restraints/parameters	12785/1/474	5712/43/258
Goodness-of-fit on F^2	1.085	1.101
Final R indexes [I>=2σ (I)]	R_1 = 0.0892, wR_2 = 0.2152	R_1 = 0.0207, wR_2 = 0.0533
Final R indexes [all data]	R_1 = 0.0925, wR_2 = 0.2198	R_1 = 0.0221, wR_2 = 0.0540
Largest diff. peak/hole / e Å^{-3}	11.27/-1.35	0.46/-0.64

Table S4. Crystal data and structure refinement for **8**.

Identification code	2297427
Empirical formula	$C_{13}H_{15}Cl_2N_3Pd$
Formula weight	390.58
Temperature/K	173.01(10)
Crystal system	monoclinic
Space group	$P2_1/c$
a/Å	9.85526(8)
b/Å	13.58959(9)
c/Å	11.48119(9)
α/°	90
β/°	107.9973(8)
γ/°	90
Volume/Å^3	1462.43(2)
Z	4
ρ_{calc}g/cm^3	1.774
μ/mm^{-1}	13.506
F(000)	776.0
Crystal size/mm^3	0.324 × 0.102 × 0.06
Radiation	Cu Kα (λ = 1.54184)
2Θ range for data collection/°	9.436 to 159.936
Index ranges	-12 ≤ h ≤ 12, -17 ≤ k ≤ 17, -14 ≤ l ≤ 14
Reflections collected	29939
Independent reflections	3184 [R_{int} = 0.0563, R_{sigma} = 0.0225]
Data/restraints/parameters	3184/0/175
Goodness-of-fit on F^2	1.098
Final R indexes [I>=2σ (I)]	R_1 = 0.0307, wR_2 = 0.0851
Final R indexes [all data]	R_1 = 0.0308, wR_2 = 0.0852
Largest diff. peak/hole / e Å^{-3}	0.95/-0.71

Table S5. Extended solid-state X-ray and solution ^{1}H NMR metrics for **$[L_1H]^+$**, **L_1** and complex **1**.[a]

	$[L_1H]^{+\,b}$	L_1	1
C1–N_{PYA}	1.401(4)	1.333(1)	1.395(4)
C2–C3	1.384(3)	1.370(2)	1.373(5)
C3–C4	1.368(5)	1.397(2)	1.369(5)
C1–N1	1.355(3)	1.378(1)	1.344(4)
N1–C5	1.353(3)	1.3648(14)	1.355(5)
N_{PYA}–C6	1.355(2)	1.343(14)	1.340(4)
O1–C6	1.216(3)	1.237(2)	1.221(4)
Pd–Cl1	---	---	2.303(9)
Pd–Cl2	---	---	2.2935(11)
$\theta^{\,c}$	89(7)	39.9(1)	87.8(4)
δ_{NCH3}	4.32	3.86	4.34
$\delta_{H(4)}$	7.83	6.73	7.54

[a] bond lengths in Å, bond angles in deg, chemical shifts in ppm (in CD_2Cl_2); [b] average value from 4 independent molecules in the asymmetric unit; [c] torsion angle between amide N–C=O group and PYA heterocycle defined by N1–C1–N_{PYA}–C6, see also Fig. S38.

Note that in **L_1** the carbonyl bond length is increased compared to **$[L_1H]^+$**, suggesting contribution from an imidate resonance structure in **L_1**. However, this zwitterionic L-type resonance form contributes less to **1** when considering that the O1-C6 bond length is essentially identical to that of the protonated ligand precursor **$[L_1H]^+$**.

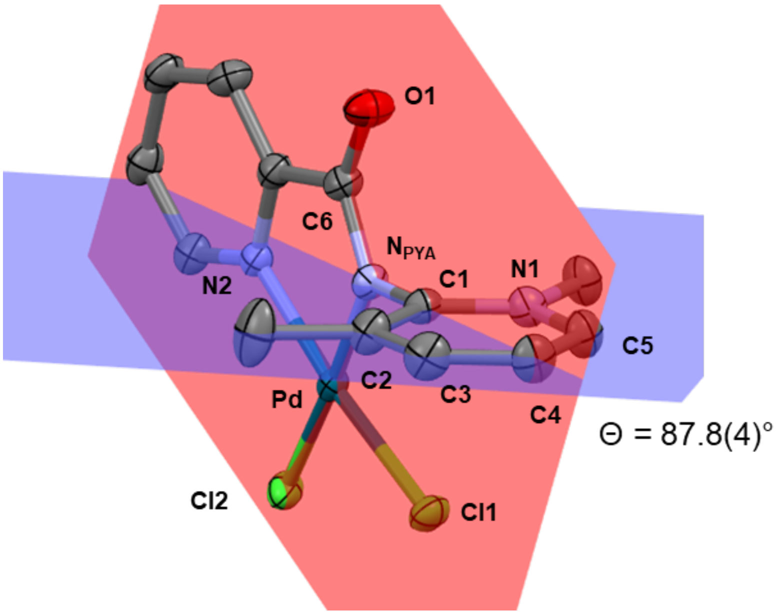

Figure S38. Graphical representation of the dihedral angle θ in complex **1.** Planes were generated from C6-N_{PYA}-C1 (red, amide plane) and N_{PYA}-C1-N1 (blue, pyridinium plane). Analogous atoms were used for the determination of θ in all other structures except **8**, where the atoms Pd-N_{PYE}-C1 were used to generate the red plane.

S.3. Buried volume calculations

Buried volume calculations were carried out using the SambVca 2.1 Software.[S8] The calculations were performed using the following parameters: Bonds radii scaled by 1.17x; Sphere radius of 3.5; Distance of the coordination point from the center of the sphere 0.0; Mesh spacing for numerical integration 0.10; H atoms were not included in the calculations.

Complex **1**:

%V Free	%V Buried	% V Tot/V Ex
59.8	40.2	99.9

Quadrant	V f	V b	V t	%V f	%V b
SW	30.6	14.3	44.9	68.1	31.9
NW	28.1	16.8	44.9	62.7	37.3
NE	24.5	20.4	44.9	54.5	45.5
SE	24.1	20.8	44.9	53.7	46.3

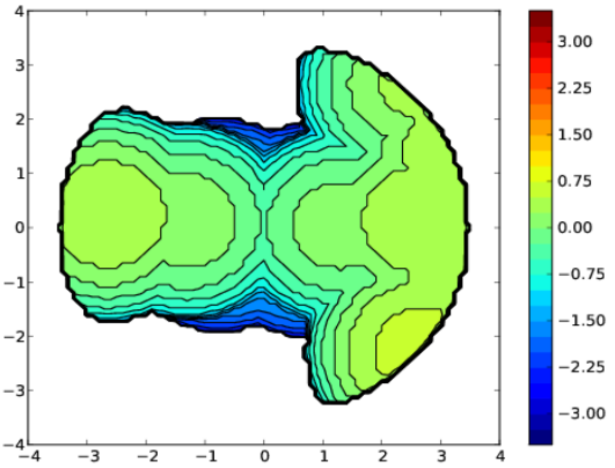

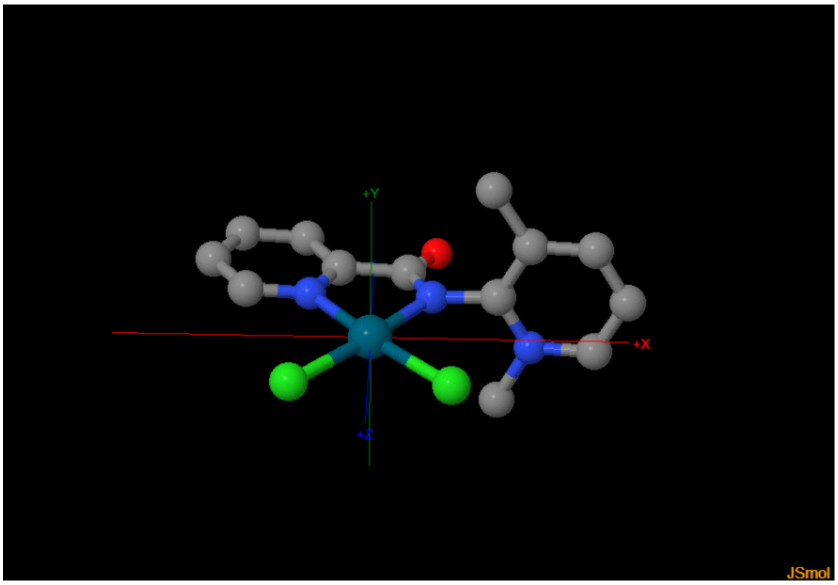

Complex **2**:

%V Free	%V Buried	% V Tot/V Ex
61.5	**38.5**	99.9

Quadrant	V f	V b	V t	%V f	%V b
SW	30.7	14.2	44.9	**68.3**	31.7
NW	28.2	16.6	44.9	**62.9**	37.1
NE	23.6	21.2	44.9	**52.7**	47.3
SE	27.8	17.0	44.9	**62.0**	38.0

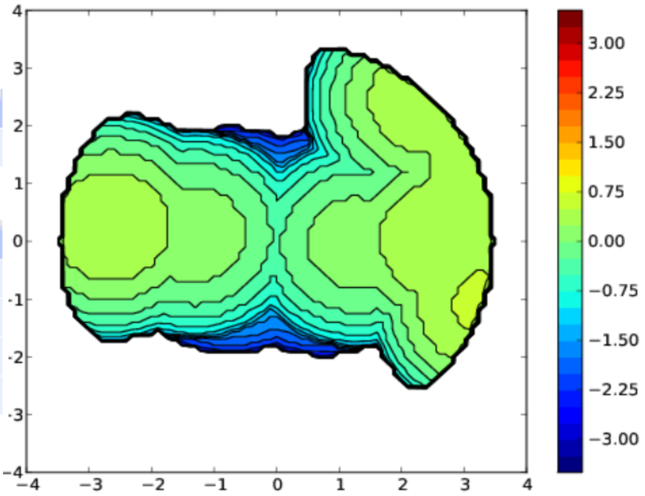

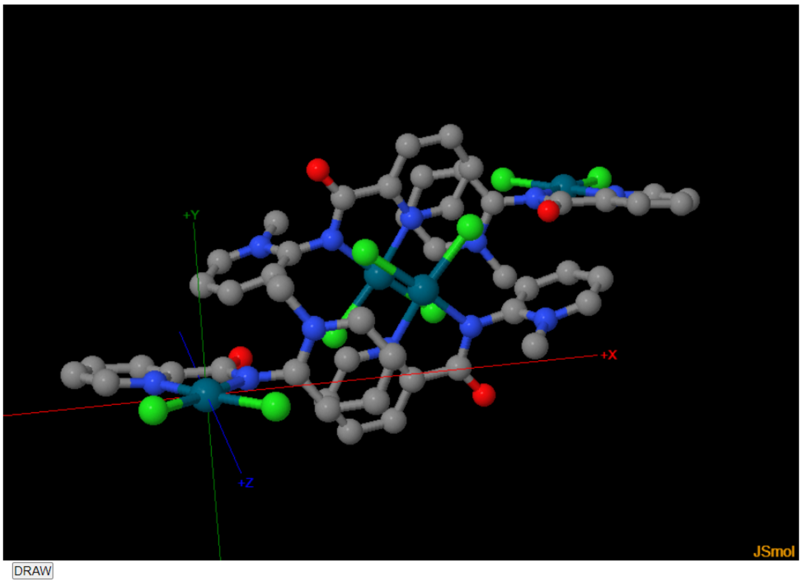

Complex **3**:

%V Free	%V Buried	% V Tot/V Ex
56.7	**43.3**	99.9

Quadrant	V f	V b	V t	%V f	%V b
SW	25.6	19.2	44.9	**57.1**	42.9
NW	29.9	14.9	44.9	**66.7**	33.3
NE	26.9	18.0	44.9	**60.0**	40.0
SE	19.3	25.6	44.9	**43.0**	57.0

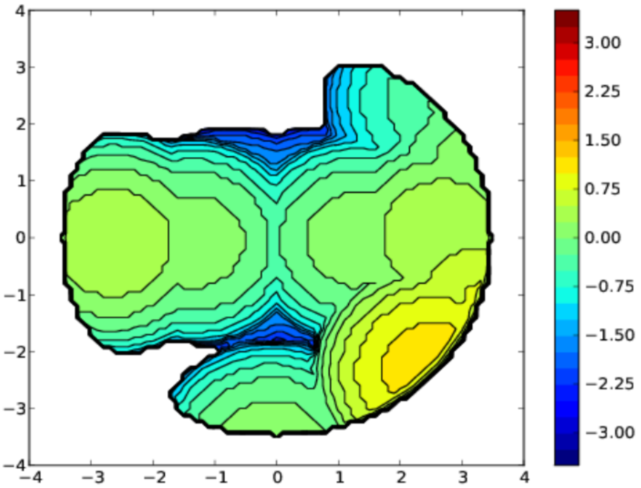

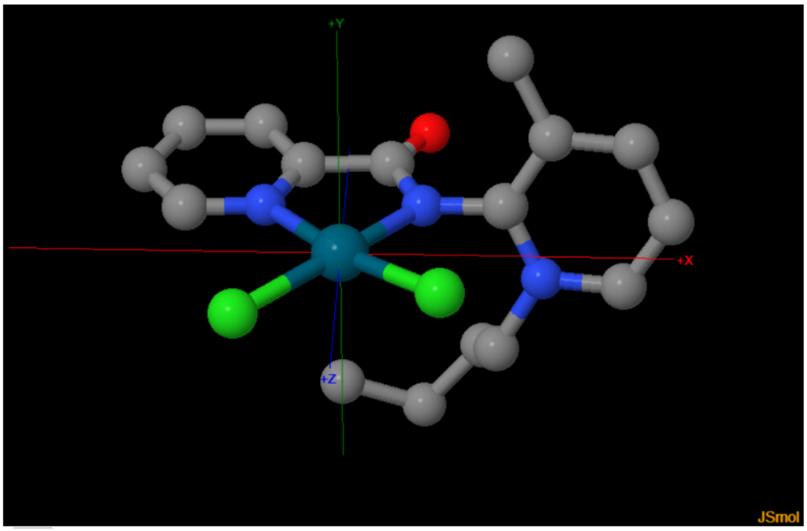

Complex **5**:

%V Free	%V Buried	% V Tot/V Ex
51.7	**48.3**	99.9

Quadrant	V f	V b	V t	%V f	%V b
SW	31.0	13.9	44.9	**69.0**	31.0
NW	13.9	31.0	44.9	**30.9**	69.1
NE	23.8	21.1	44.9	**53.0**	47.0
SE	24.2	20.7	44.9	**53.9**	46.1

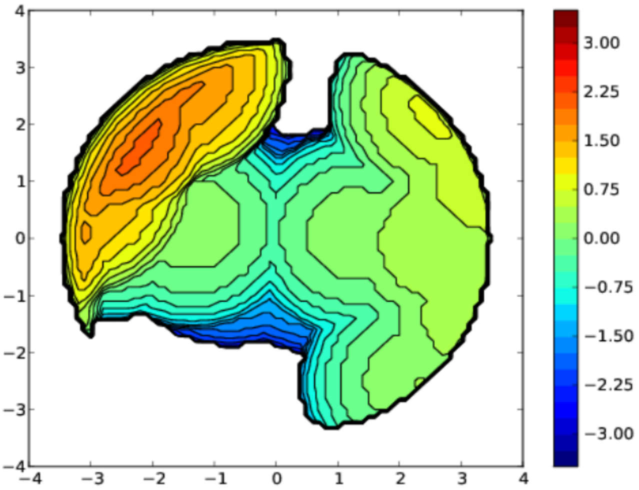

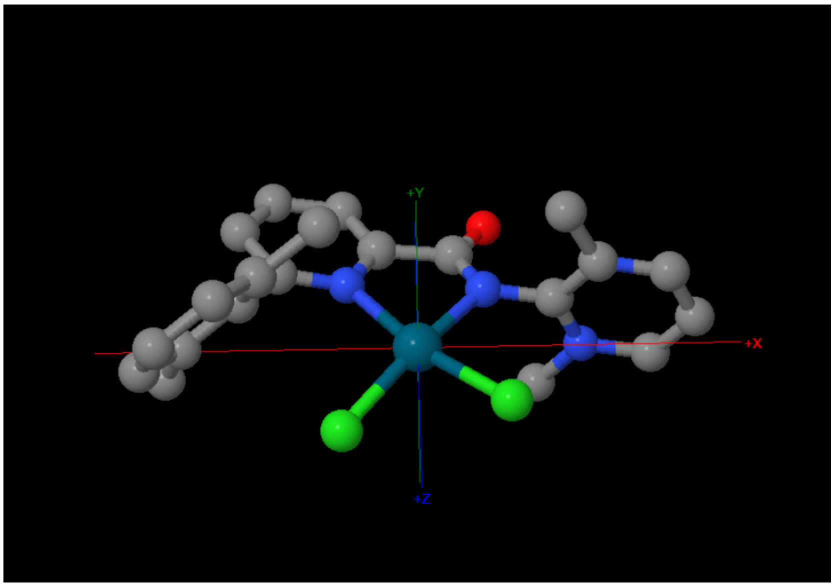

Complex **8**:

%V Free	%V Buried	% V Tot/V Ex
58.4	**41.6**	99.9

Quadrant	V f	V b	V t	%V f	%V b
SW	28.3	16.5	44.9	**63.1**	36.9
NW	30.1	14.8	44.9	**67.0**	33.0
NE	25.1	19.8	44.9	**55.9**	44.1
SE	21.3	23.6	44.9	**47.4**	52.6

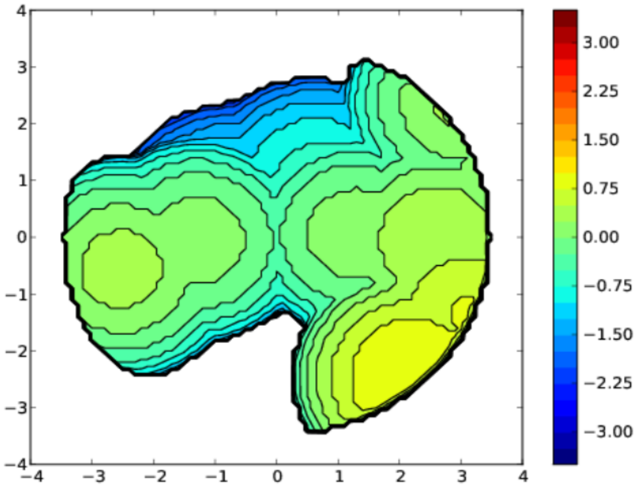

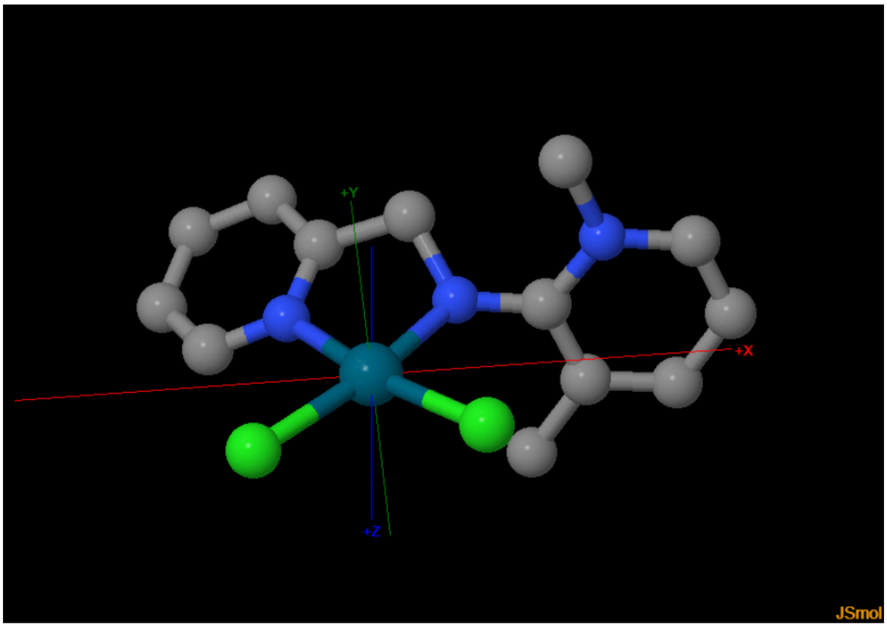

S.4. Catalytic α-arylation of ketones

Typical procedure: NaOtBu (106 mg, 1.1 mmol), complex **1** (4.05 mg, 0.01 mmol) and hexamethylbenzene (32.5 mg, 0.2 mmol) were placed in a 10 mL microwave vial that was closed, evacuated and backfilled with N_2 three times. Dry 1,4-dioxane (1.0 mL), ketone (1.0 mmol) and aryl halide (1.0 mmol) were then added in this order. The resulting suspension was heated at 105°C and vigorously stirred for the indicated time. The course of the reaction was followed by taking aliquots (20 µL) and diluting them with either $CDCl_3$ or wet 1,4-dioxane for NMR or GC-MS analysis, respectively. At the end of the reaction the suspension was poured into H_2O (10 ml), extracted with CH_2Cl_2 (5 x 10 mL), dried over Na_2SO_4, filtered and concentrated under vacuum. If necessary, the crude product was purified by gradient flash column chromatography (0 to 15% EtOAC in n-hexane; SiO_2).

S.4.1. Optimization of reaction conditions

Table S6. Screening of different bases for the α-arylation of propiophenone with bromobenzene.[a]

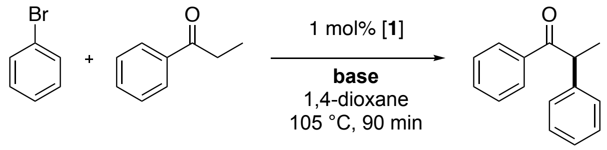

entry	base	mol eq.	spectroscopic yield[b]	ketone conversion
1	none	---	<1%	<1%
2	DBU	1.1	<1%	<1%
3	Cs_2CO_3	1.1	4%	5%
4	K_3PO_4	1.1	3%	4%
5	NaO*t*Bu	1.0	67%	79%
**6**	**NaO*t*Bu**	**1.1**	**91%**	**94%**
7	NaO*t*Bu	1.2	90%	98%
8	NaO*t*Bu	1.3	77%	94%
9[c]	NaO*t*Bu (THF solution)	1.1	77%	95%
10[d]	NaO*t*Bu + THF	1.1	73%	84%
11[e]	NaO*t*Bu (THF solution)	1.8	45%	75%
12	LiO*t*Bu	1.1	72%	76%
13	KO*t*Bu	1.1	59%	84%

[a] Reaction conditions: ArBr (1.0 mmol), propiophenone (1.0 mmol), [**1**] (0.01 mmol), 1,4-dioxane (1 mL) in a 10 mL microwave vial, 105 °C for 90 min under N_2; [b] yields determined by GC analysis with hexamethylbenzene as internal standard; [c] NaO*t*Bu (2 M in THF, 0.53 mL, 1.1 mmol) was used yielding a dioxane/THF ratio of ~65:35; [d] THF (53 µL) was added to the reaction mixture to mimic the solvent ratio of entry 9; [e] NaOtBu solution (2 M in THF, 0.87 mL, 1.8 mmol) was used yielding a dioxane/THF ratio of ~53/47.

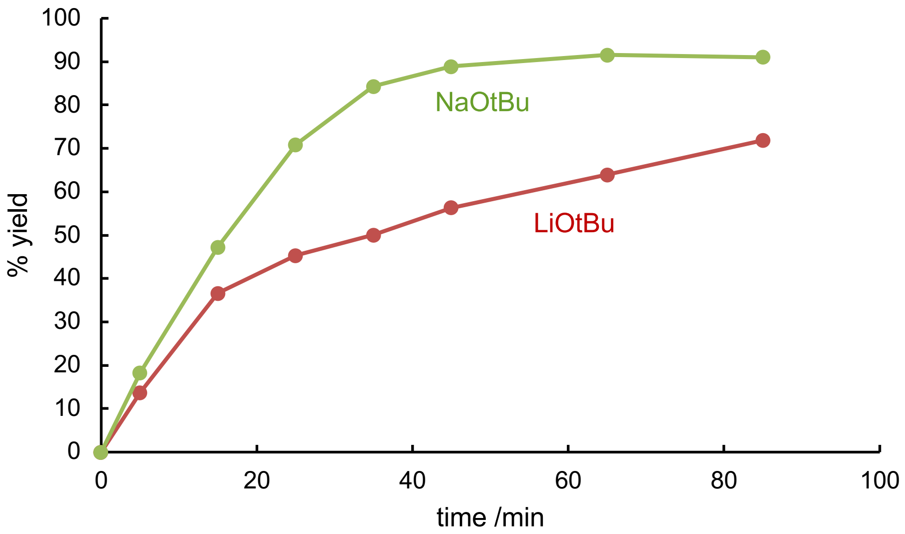

Figure S39. Influence of the base cation on the reaction rate. Conditions: ArBr (1.0 mmol), propiophenone (1.0 mmol), **2** (0.01 mmol), base (1.1 mmol), solvent (1.0 mL) in a 10 mL microwave vial, 105 °C for 90 min under N_2. Yields determined by GC analysis with hexamethylbenzene as internal standard.

Table S7. Screening of solvents for the α-arylation of propiophenone with bromobenzene.[a]

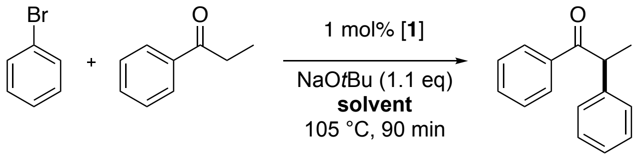

entry	solvent	spectroscopic yield [b]	ketone conversion	PhBr conversion
1	*n*BuOH	3%	60%	88%
2	EtOH	3%	66%	89%
3	$MeNO_2$	<1%	<1%	<1%
4	MeCN	2%	79%	18%
5	Toluene	47%	71%	63%
6	THF	41%	67%	63%
7	2-MeTHF	58%	67%	69%
**8**	**1,4-dioxane**	**91%**	**94%**	**99%**

[a] Reaction conditions: ArBr (1.0 mmol), propiophenone (1.0 mmol), [**1**] (0.01 mmol), NaO*t*Bu (1.1 mmol), solvent (1.0 mL) in a 10 mL microwave vial, 105 °C for 90 min under N_2; [b] yields and conversions determined by GC analysis with hexamethylbenzene as internal standard.

Table S8. Influence of the concentration of the reactants on the α-arylation of propiophenone.[a]

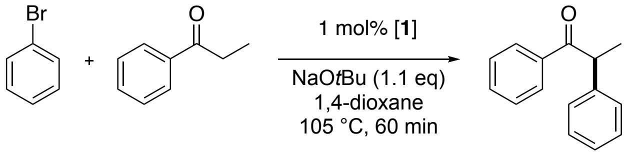

entry	substrate concentration	spectroscopic yield[b]	ketone conv	PhBr conv	TOF_{max} /h^{-1}
1[c]	0.1 M	8%	36%	34%	---
2	0.5 M	77%	91%	89%	---
3	1.0 M	91%	94%	>99%	220
**4**	**2.0 M**	**87%**	**96%**	**>99%**	**280**
5	4.0 M	80%	86%	85%	270
6[d]	8.0 M	85%	86%	87%	360

[a] Reaction conditions: Bromobenzene (1.0 mmol), propiophenone (1.0 mmol), complex **1** (0.01 mmol), NaO*t*Bu (1.1 mmol), 1,4-dioxane (10 to 0.25 mL) in a 10 mL microwave vial, 105 °C for 1 h under N_2; [b] yields and conversions determined by GC analysis with hexamethylbenzene as internal standard; [c] reaction was performed in a 30 mL microwave vial. [d] all the quantities were doubled and the solvent volume used was 0.25 mL.

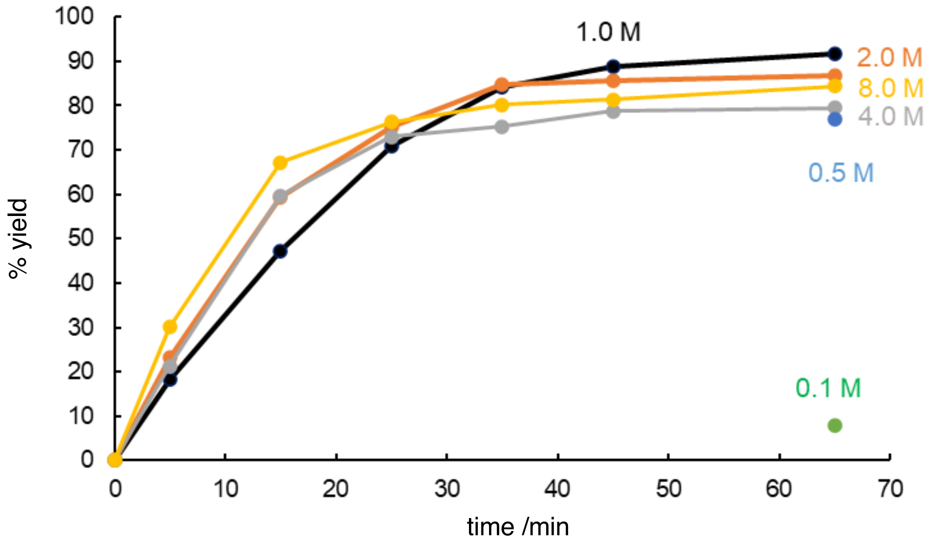

Figure S40. Time conversion profile for the α-arylation of propiophenone with bromobenzene catalyzed by **1** at different substrate concentrations (molarity with respect to propiophenone); ratio of ketone/ bromobenzene/ base /catalyst consistently at 1:1:1.1:0.01 as detailed in Table S8.

Table S9. Variation of the substrate stoichiometry in the α-arylation of propiophenone.[a]

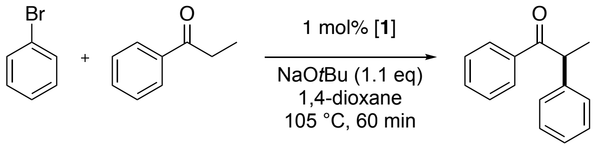

entry	substrate in excess	spectroscopic yield	ketone conversion	PhBr conversion
1	none	87%	96%	>99%
2 [b]	PhBr	92%	97%	82%
3 [c]	ketone	86%	86%	>99%

[a] Reaction conditions: PhBr (1.0 mmol), propiophenone (1.0 mmol), complex **1** (0.01 mmol), NaO*t*Bu (1.1 mmol), 1,4-dioxane (0.5 mL) in a 10 mL microwave vial, 105 °C, 60 min, under N_2, yields relative to the limiting substrate; [b] PhBr (1.2 mmol); [c] propiophenone (1.2 mmol).

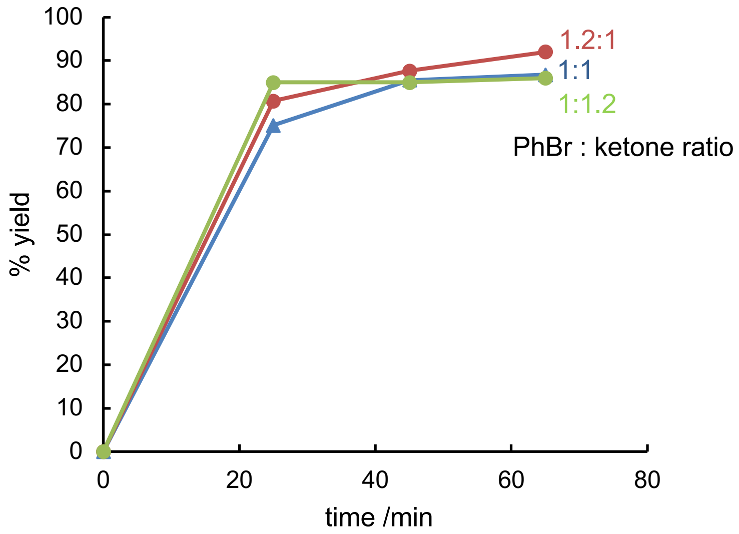

Figure S41. Conversion profile for the α-arylation of propiophenone with bromobenzene at different reagent stoichiometries; for conditions see Table S9.

Table S10. Effect of the reaction temperature on the α-arylation of propiophenone catalyzed by complexes **1**.[a]

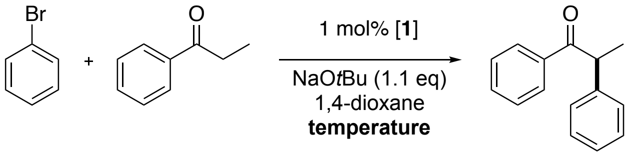

entry	temperature / °C	spectroscopic yield	ketone conversion	PhBr conversion	TOF_{max} /h^{-1}
1	85	70% [b]	76%	72%	30
2	95	71% [b]	91%	83%	58
3	105	87%	96%	>99%	280
4	115	85%	96%	98%	450
5	125	86%	97%	99%	1040

[a] Reaction conditions: ArBr (1.0 mmol), propiophenone (1.0 mmol), complex **1** (0.01 mmol), NaO*t*Bu (1.1 mmol), 1,4-dioxane (0.5 mL) in a 10 mL microwave vial, 1 h reaction time under N_2; [b] spectroscopic yield after 150 min.

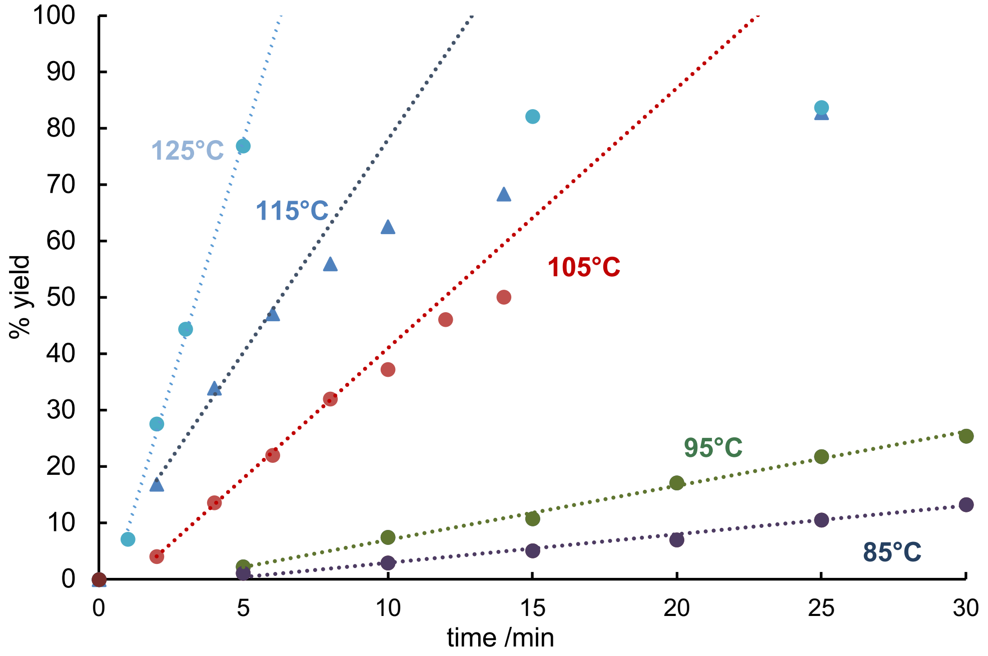

Figure S42. Time dependent conversion profiles for the coupling of propiophenone and bromobenzene catalyzed by complex **1** at different temperatures, dotted lines indicate initial rates. Reaction conditions: ArBr (1.0 mmol), propiophenone (1.0 mmol), complex **1** (0.01 mmol), NaO*t*Bu (1.1 mmol), 1,4-dioxane (0.5 mL) in a 10 mL microwave vial. Spectroscopic yields measured by GC-FID with hexamethylbenzene used as internal standard.

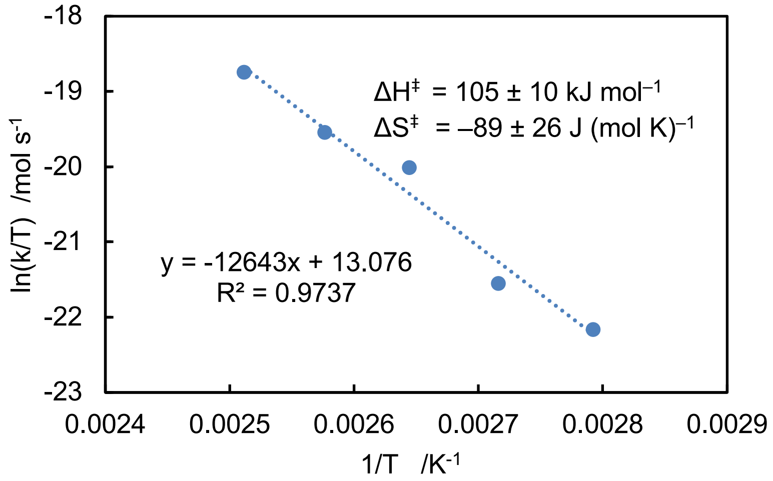

Figure S43. Eyring plot for the α-arylation of propiophenone catalyzed by complex **1**. Least-square regression and integration of standard errors from the least-square method gives a slope of –12,643 (±1199) and an intercept of 13.08 (±3.18). From the Eyring equation (1)

$$\ln(k/T) = (1/T) \times \Delta H^{\ddagger}/R + \ln(k_B/h) + \Delta S^{\ddagger}/R \qquad (1)$$

$\Delta H^{\ddagger}$ was calculated from the slope,[S9] as follows:

$$\mathbf{\Delta H^{\ddagger}} = 12{,}643\ (\pm 1199) \times 8.31 = \mathbf{105.1\ (\pm 9.9)\ kJ\ mol^{-1}}$$

$\Delta S^{\ddagger}$ was calculated from the intercept as follows:

$$13.076\ (\pm 3.178) = \ln(k_B/h) + \Delta S^{\ddagger}/R$$

and hence: $\mathbf{\Delta S^{\ddagger}} = [13.08\ (\pm 3.18) - 23.76] \times 8.31 = \mathbf{-88.8\ (\pm 26.4)}\ \mathrm{J\ (K\ mol)^{-1}}$

S.4.2. Variation of catalyst precursors

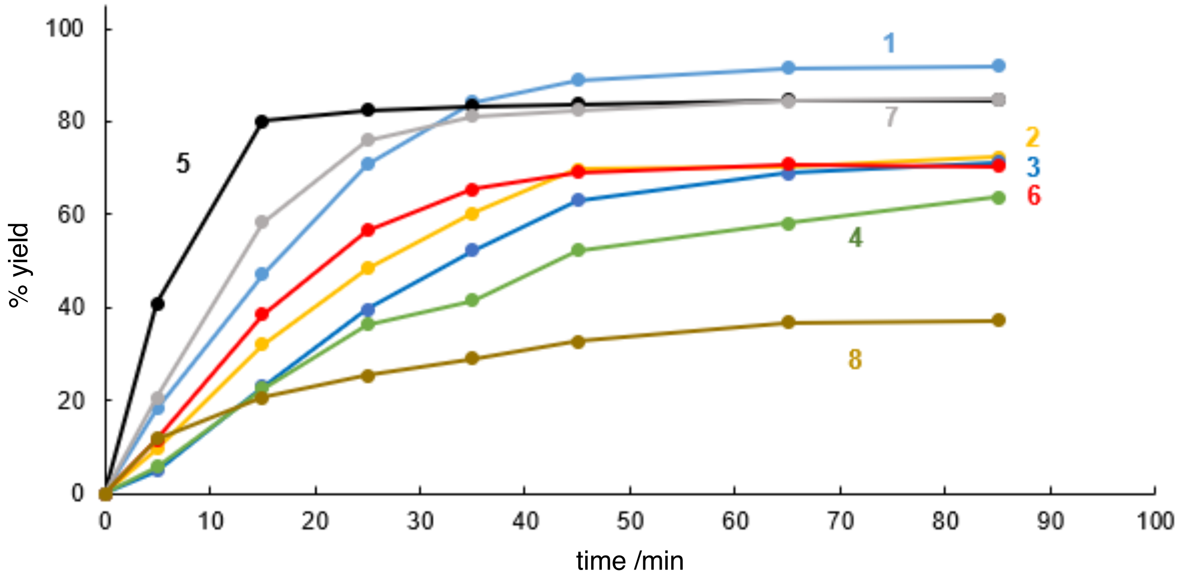

Figure S44. Time dependent conversion profile of the α-arylation of propiophenone with bromobenzene catalyzed by different palladium complexes. Reaction conditions: ArBr (1.0 mmol), propiophenone (1.0 mmol), [Pd] (0.01 mmol), NaO*t*Bu (1.1 mmol), 1,4-dioxane (1.0 mL) in a 10 mL microwave vial, 105 °C, 90 min, N_2 atmosphere.

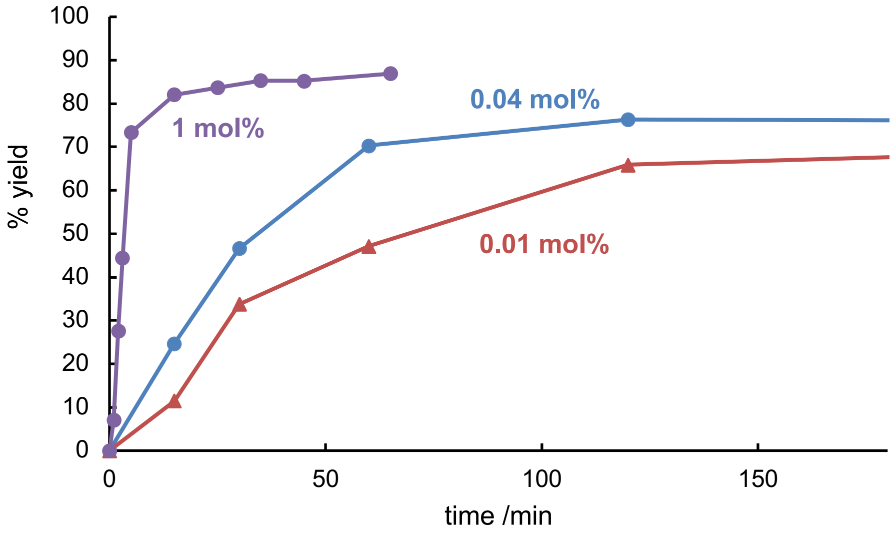

Figure S45. Time-conversion profile for the α-arylation of propiophenone upon lowering the catalyst loading. Reaction conditions for 1 mol% catalyst loading: PhBr (1.0 mmol), propiophenone (1.0 mmol), complex **1** (0.01 mmol), NaO*t*Bu (1.1 mmol), 1,4-dioxane (0.5 mL) in a 10 mL microwave vial, 90 min at 125°C; for 0.04 mol% catalyst loading: PhBr (3.09 mmol), propiophenone (3.09 mmol), complex **1** (1.24 µmol), NaO*t*Bu (3.40 mmol), 1,4-dioxane (1.55 mL) in a 10 mL microwave vial, 90 min at 125 °C. For 0.01 mol% catalyst loading: PhBr (12.36 mmol), propiophenone (12.36 mmol), complex **1** (1.24 µmol), NaO*t*Bu (13.59 mmol), 1,4-dioxane (6.2 mL) in a 25 mL microwave vial, 90 min at 125 °C.

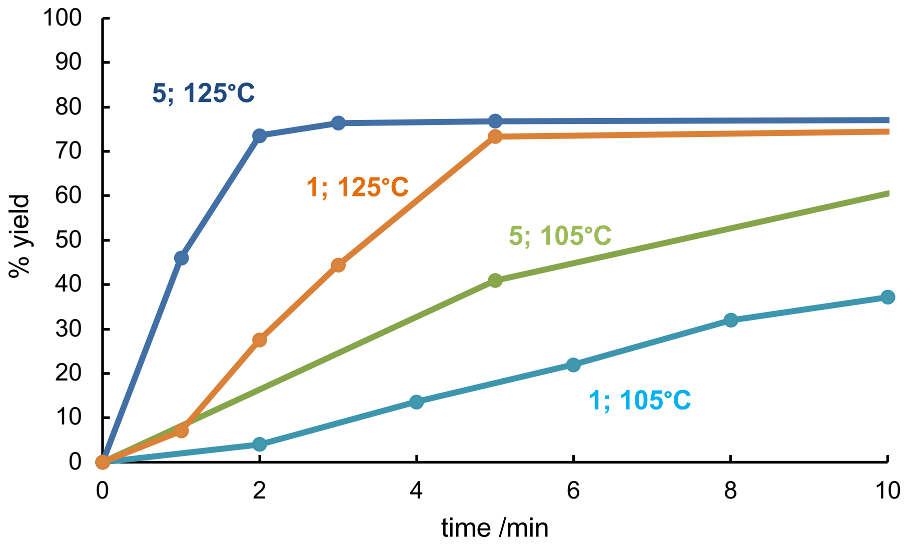

Figure S46. Time conversion profiles of the α-arylation of propiophenone with complexes **1** and **5** at 105 and 125 °C. Reaction conditions: PhBr (1.0 mmol), propiophenone (1.0 mmol), [Pd] (0.01 mmol), NaO*t*Bu (1.1 mmol), 1,4-dioxane (0.5 mL) in a 10 mL microwave vial.

S.4.3. Variation of substrates

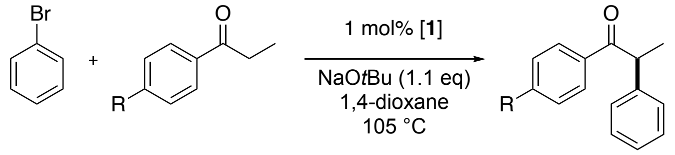

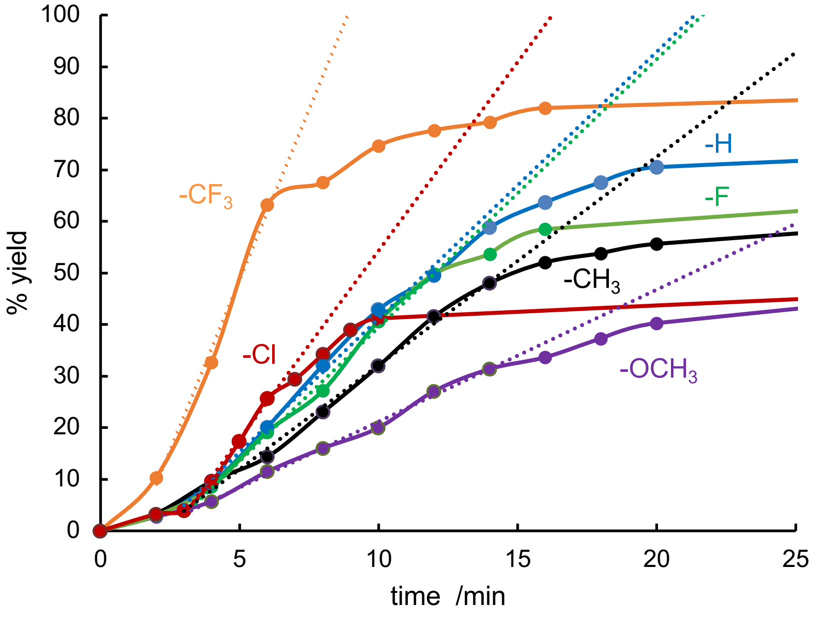

Figure S47. Maximum rate determination for the α-arylation of various *para*-substituted propiophenones with bromobenzene. Reaction conditions: PhBr (1.2 mmol), propiophenone (1.0 mmol), complex **1** (0.01 mmol), NaO*t*Bu (1.1 mmol), 1,4-dioxane (0.5 mL) in a 10 mL microwave vial at 105°C. Spectroscopic yields determined by GC-FID relative to hexamethylbenzene as internal standard. Dotted lines represent maximum rates.

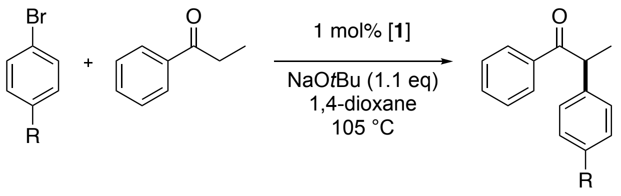

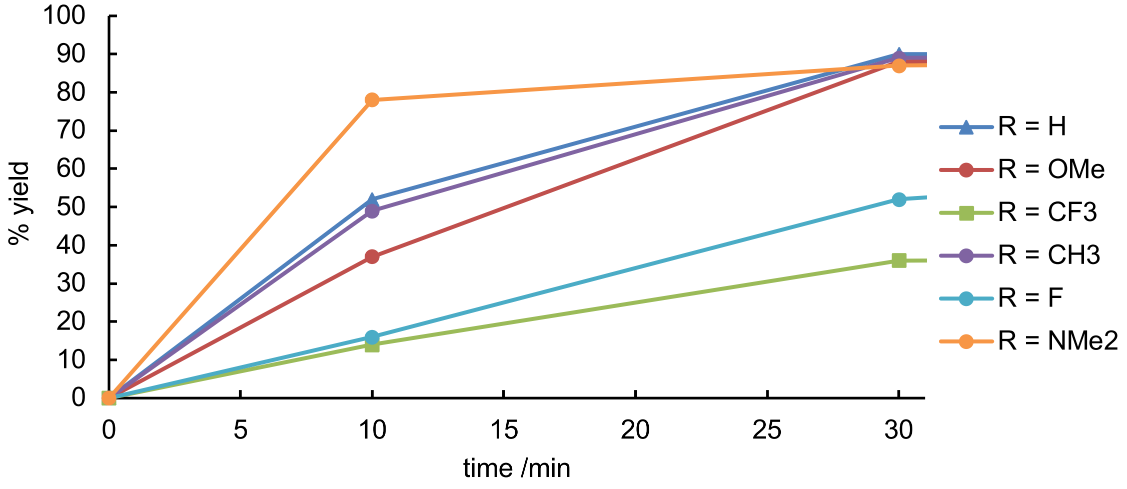

Figure S48. Initial rate determination (TOF_{10min}) for the α-arylation of propiophenone with various *para*-substituted bromobenzenes. Reaction conditions: ArBr (1.0 mmol), propiophenone (1.2 mmol), complex **1** (0.01 mmol), NaO*t*Bu (1.32 mmol), 1,4-dioxane (0.5 mL) in a 10 mL microwave vial at 105 °C. Spectroscopic yields determined by GC-FID relative to hexamethylbenzene as internal standard.

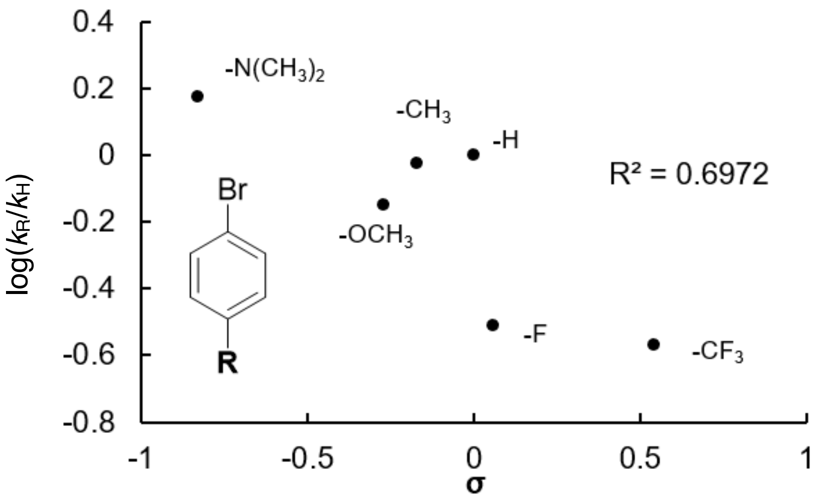

Figure S49. Hammett plot for the arylation of 4-substituted propiophenones (see Fig S48 for reaction conditions and initial rates) showing a very poor correlation.

Table S11. Reaction optimization for the α-arylation of propiophenone with phenyl chloride catalyzed by complex **1**.[a]

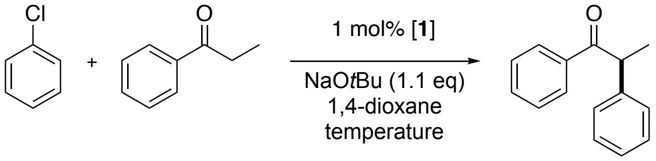

entry	temperature /°C	cat. loading /%	eq. PhCl	ketone conv /%	yield /%	TON
1	105	1	1.2	30 %	8	8
2	125	1	1.2	57 %	15	15
3	135	1	1.2	31%	7	7
4	135	1	1.0	33%	6	6
5	150	1	1.2	38%	8	8
6	150	1	2.0	38%	8	8
7	125	2.5	1.2	47%	7	2.8
8	125	5	1.2	63%	18	4.5

[a] Reaction conditions: PhCl (1.0–2.0 mmol), propiophenone (1.0 mmol), complex **1** (0.01- 0.05 mmol), NaO*t*Bu (1.1 mmol), 1,4-dioxane (0.5 mL) in a 10 mL microwave vial, 60 min at indicated temperature; yields did not increase upon extension of reaction times.

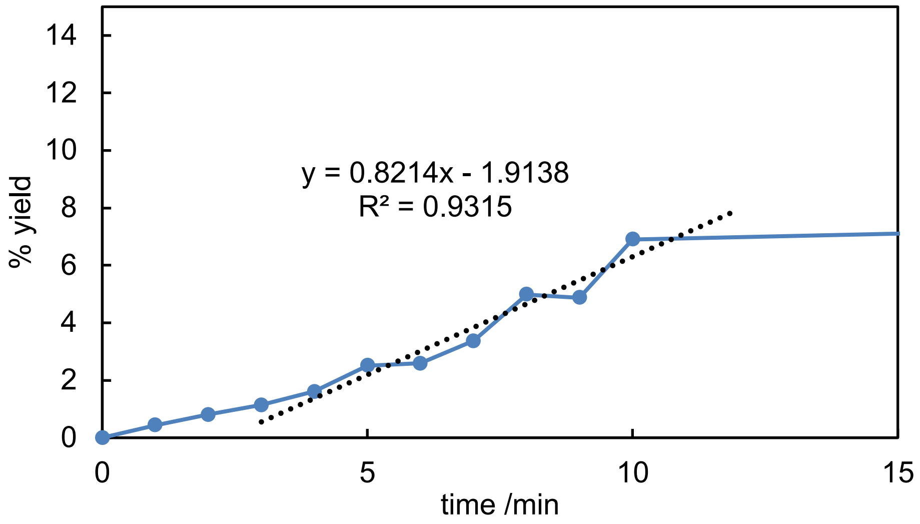

Figure S50. Initial time conversion profile for the α-arylation of propiophenone with chlorobenzene as substrate, dotted line indicates maximum rate (TOF_{max} = 50 h^{-1}). The yield after 60 min is 9%, barely higher than the yield after 10 min. Reaction conditions: PhCl (1.2 mmol), propiophenone (1.0 mmol), complex **1** (0.01 mmol), NaO*t*Bu (1.1 mmol), 1,4-dioxane (1.0 mL) in a 10 mL microwave vial at 125 °C.

Substrate scope

^{1}H NMR spectroscopy was typically used to determine spectroscopic yields of α-arylated ketone products. Hexamethylbenzene was used as internal standard. Aliquots (20 μL) were taken from the reaction mixture after purging a syringe three times with nitrogen gas and were directly dissolved in $CDCl_3$ (0.3 mL) and analysed by ^{1}H NMR spectroscopy.

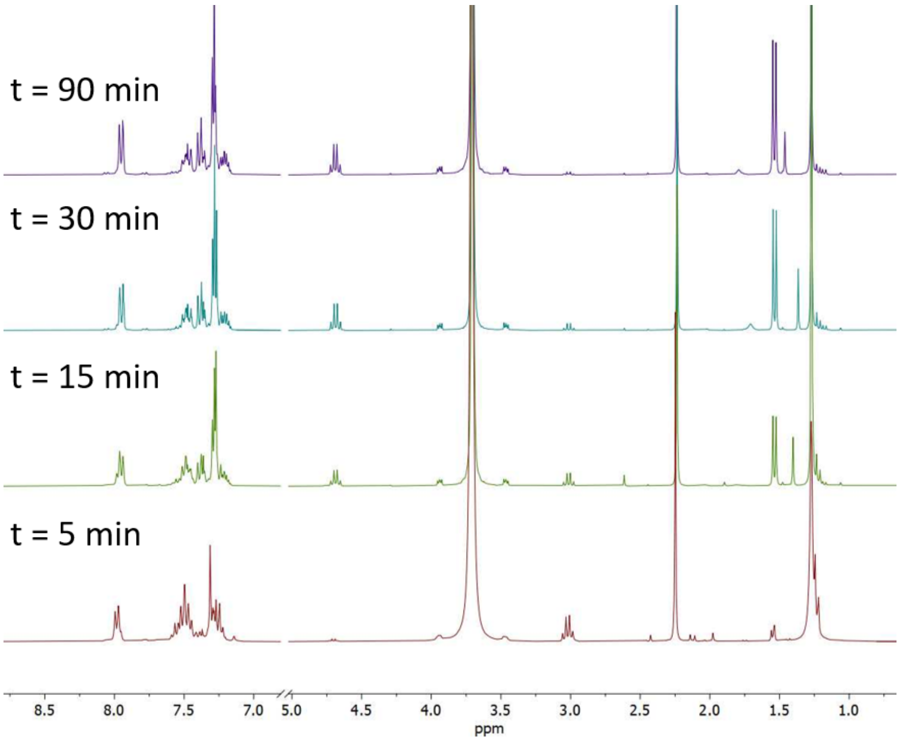

Figure S51. Stacked ^{1}HNMR spectra of a typical catalytic run of bromobenzene (**9d**) and propiophenone (**10c**) in 1,4-dioxane.

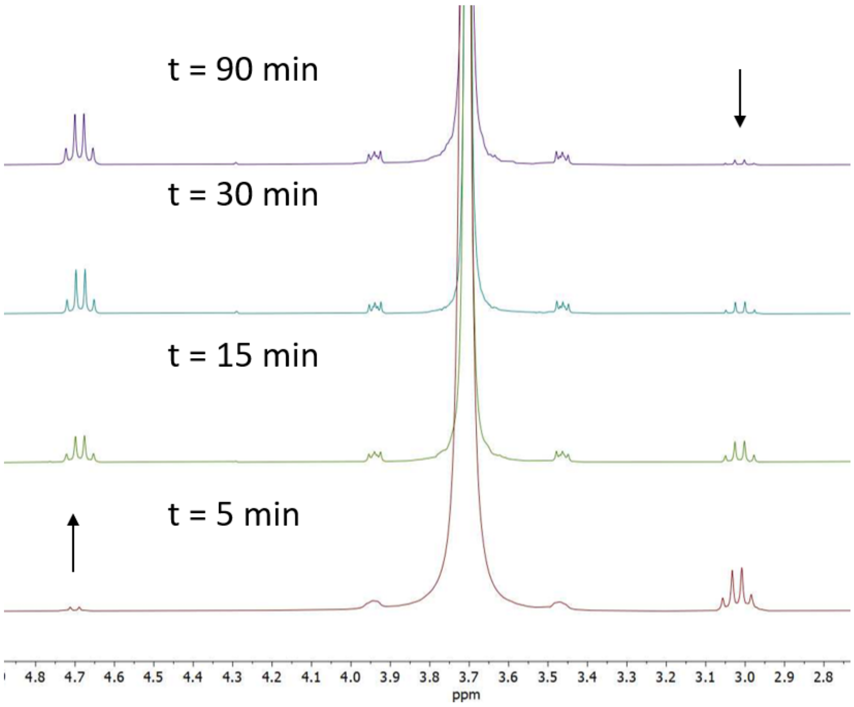

Figure S52. Zoom of the Stacked ^{1}H NMR spectra from Fig. S51 indicating the signals at δ_H = 3.0 and 4.7 used for quantifying conversion and yield, respectively.

S.4.4. Characterization of products

All products were characterized as crude mixtures or isolated products when the ^{1}H NMR spectroscopic yield was not clear.

Product 11ac

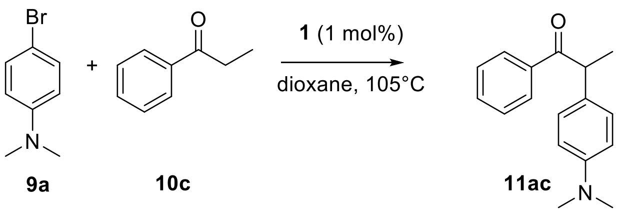

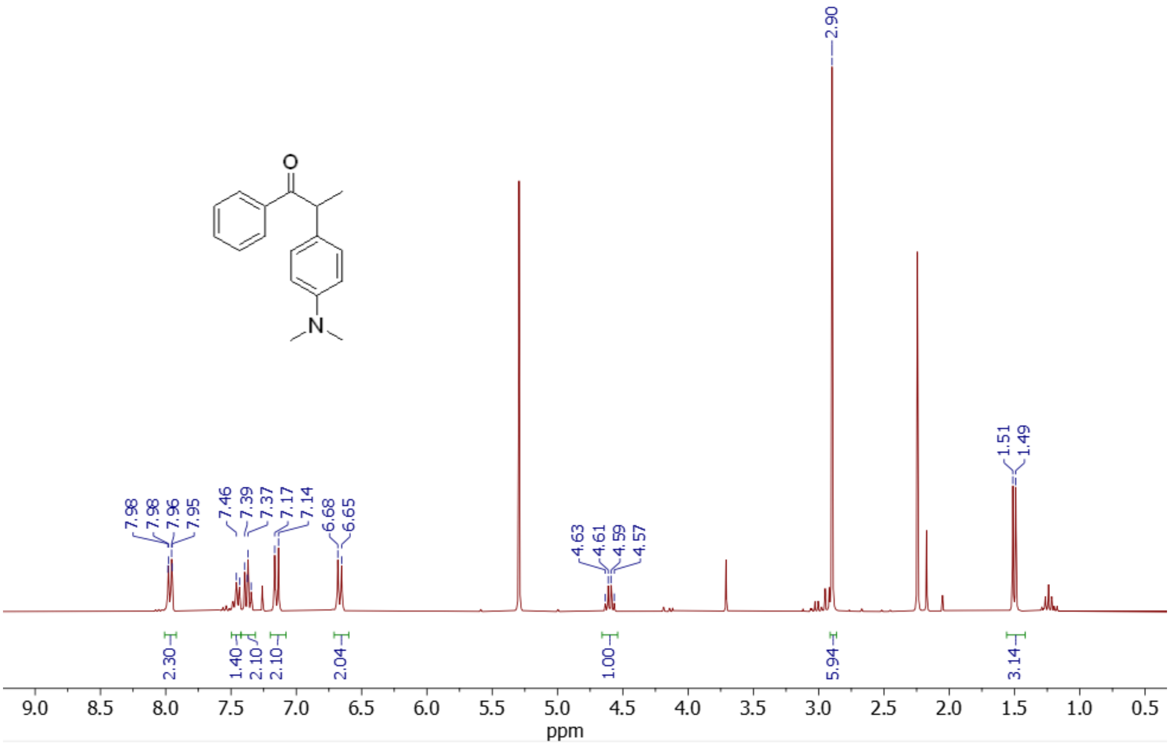

Figure S53. ^{1}H NMR spectrum ($CDCl_3$, 298 K, 300 MHz) of **11ac** from the crude reaction mixture, in agreement with literature data.[S10] HR-ESI-MS (m/z): calculated for $C_{17}H_{20}NO$ $[M+H]^+$ = 254.1545; found: 254.1536.

Product 10bc

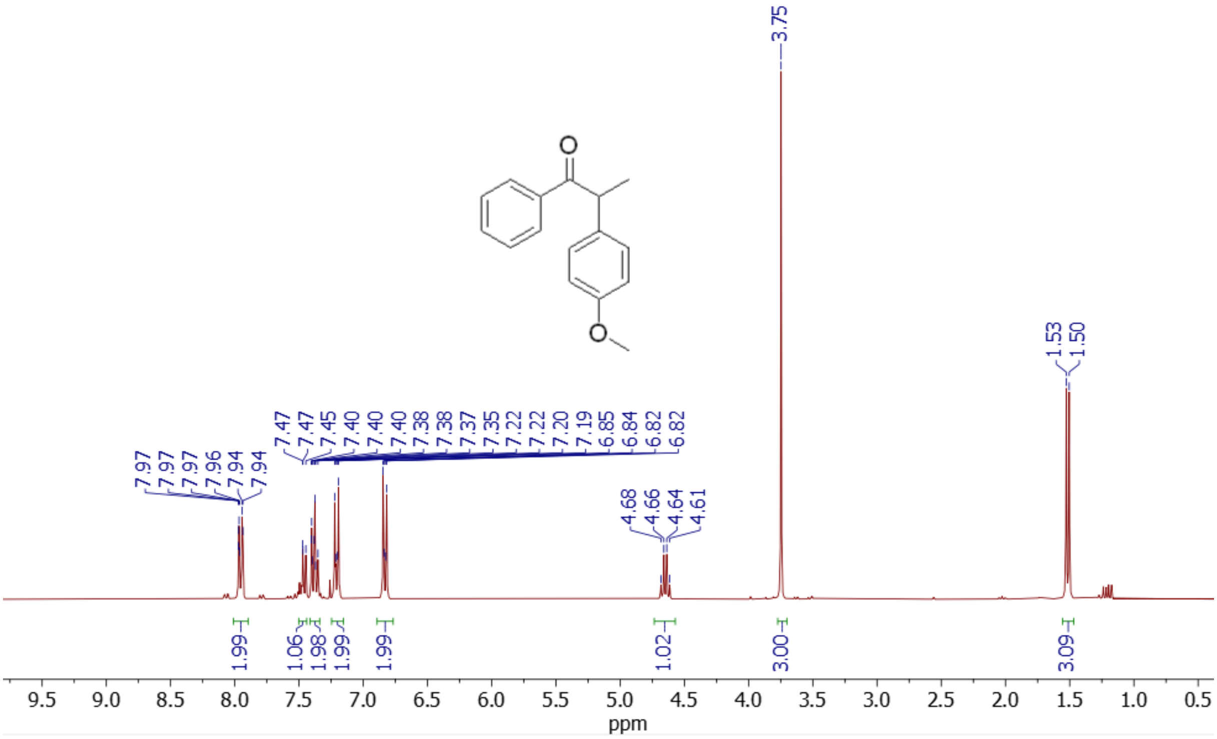

Figure S54. ^{1}H NMR spectrum ($CDCl_3$, 298 K, 300 MHz) of isolated **11bc,** in agreement with literature data.[S11] HR-MS (m/z): calculated for $C_{16}H_{17}O_2$ $[M+H]^+$ = 241.1229; found: 241.1222.

Product 11cc

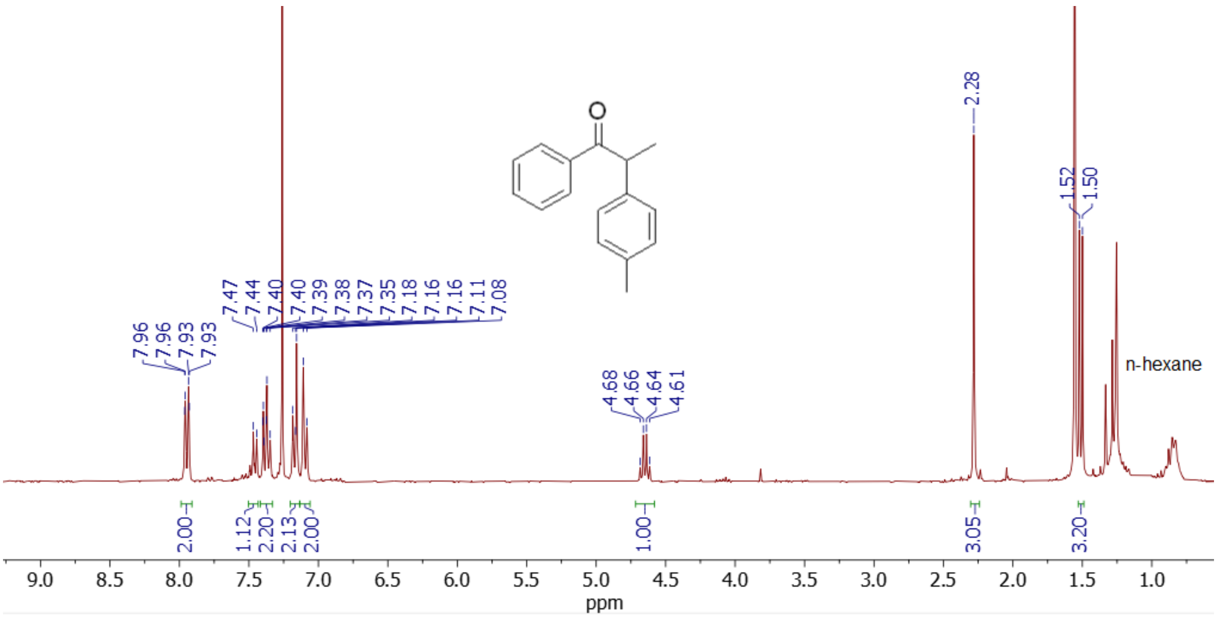

Figure S55. ^{1}H NMR spectrum ($CDCl_3$, 298 K, 300 MHz) of isolated **11cc,** in agreement with literature data.[S11] HR-EI-MS (m/z): calculated for $C_{16}H_{18}O$ = 224.1201; found: 224.1196.

Product 11dc (also **11jc** and **11kc**)

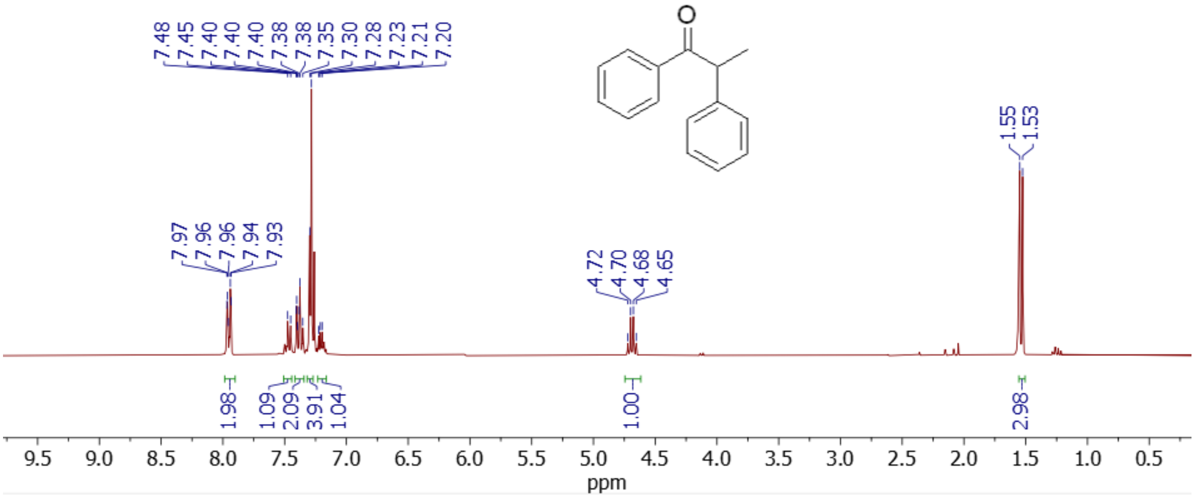

Figure S56. ^{1}H NMR spectrum ($CDCl_3$, 298 K, 300 MHz) of isolated **11dc,** in agreement with literature data.[S11] HR-MS (m/z): calculated for $C_{15}H_{16}O$ $[M+H]^+$ = 211.1123; found: 211.1127. Note: Products **11jc** and **11kc** had identical analytics (same specie).

Product **11ec**

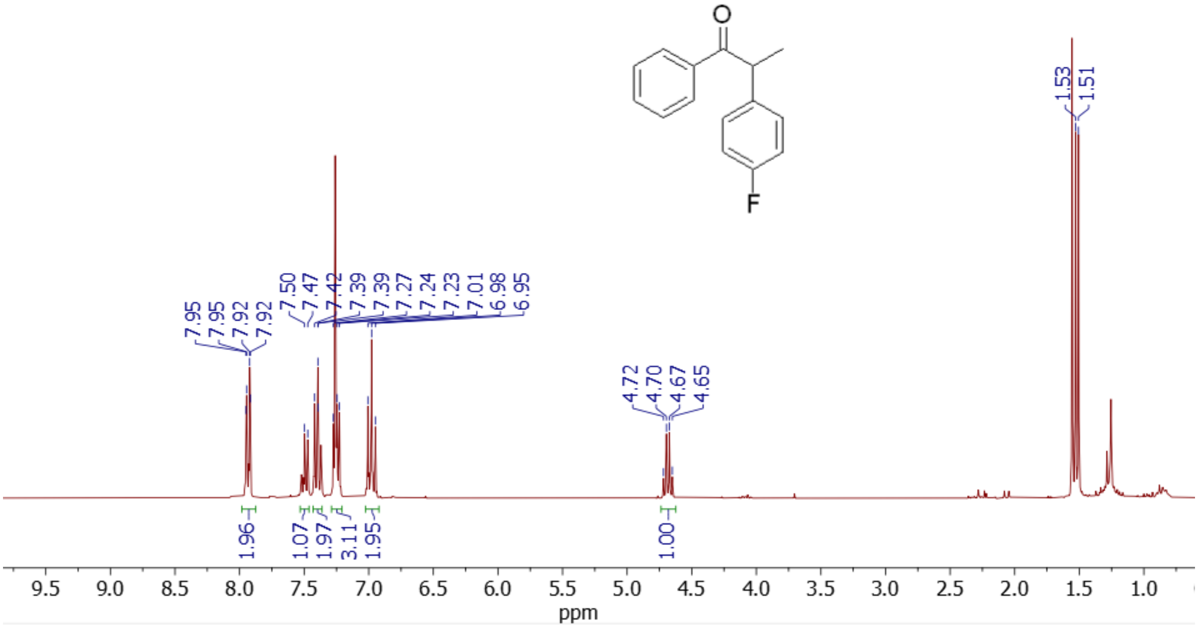

Figure S57. ^{1}H NMR spectrum ($CDCl_3$, 298 K, 300 MHz) of isolated **11ec,** in agreement with literature data.[S12] HR-EI-MS (m/z): calculated for $C_{15}H_{13}FO$ = 228.0950; found: 228.0942.

Product 11fc

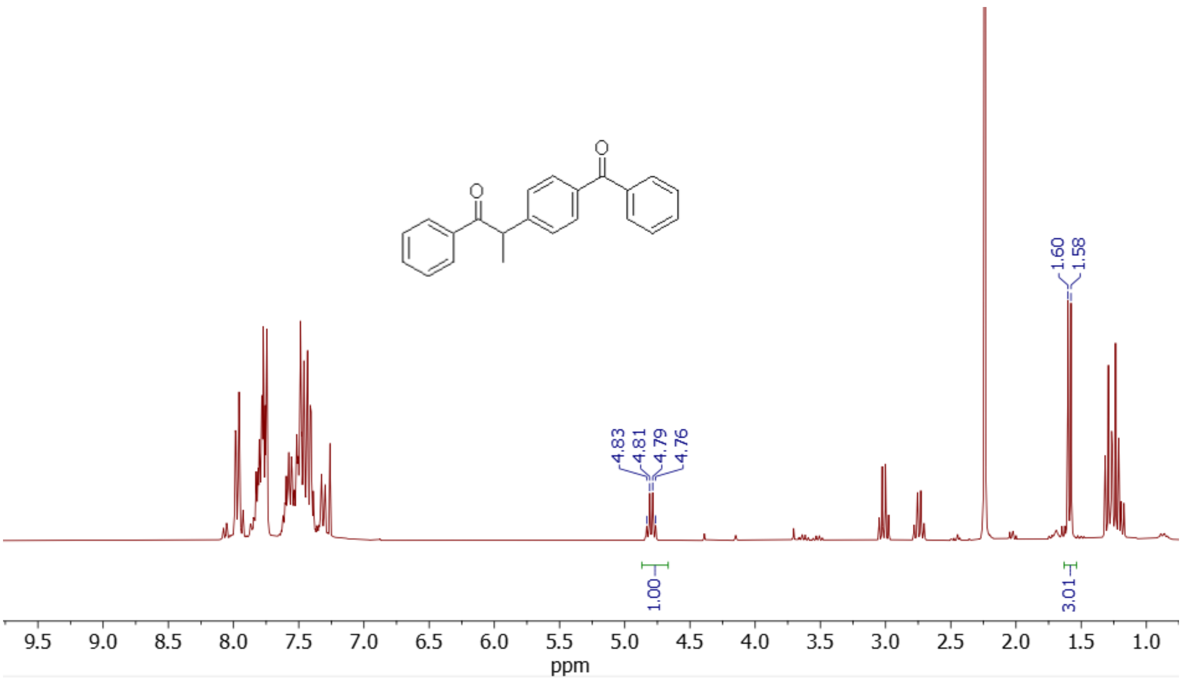

Figure S58. ^{1}H NMR spectrum ($CDCl_3$, 298 K, 300 MHz) of crude **11fc** from the reaction mixture, in agreement with literature data.[S13] Due to low conversion, only the aliphatic protons were integrated. Conversion was calculated from the crude mixture. HR-EI-MS (m/z): calculated for $C_{22}H_{18}O_2$ = 314.1307; found: 314.1304.

Product 11gc

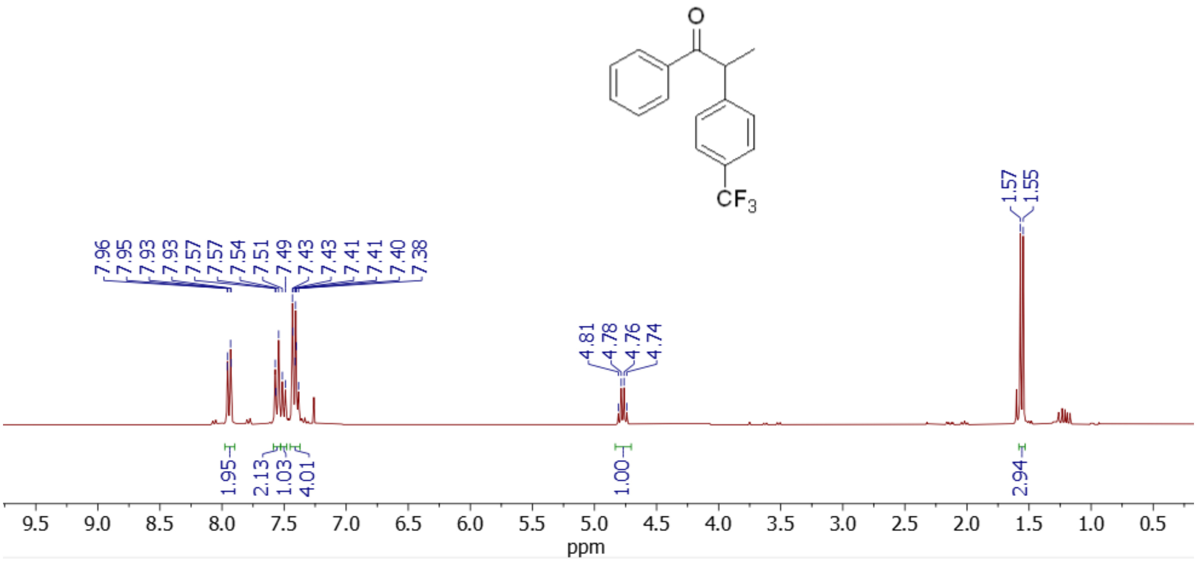

Figure S59. ^{1}H NMR spectrum ($CDCl_3$, 298 K, 300 MHz) of isolated **11gc,** in agreement with literature data.[S10] HR-EI-MS (m/z): calculated for $C_{16}H_{13}F_3O$ = 278.0918; found: 278.0918.

Product 11hc

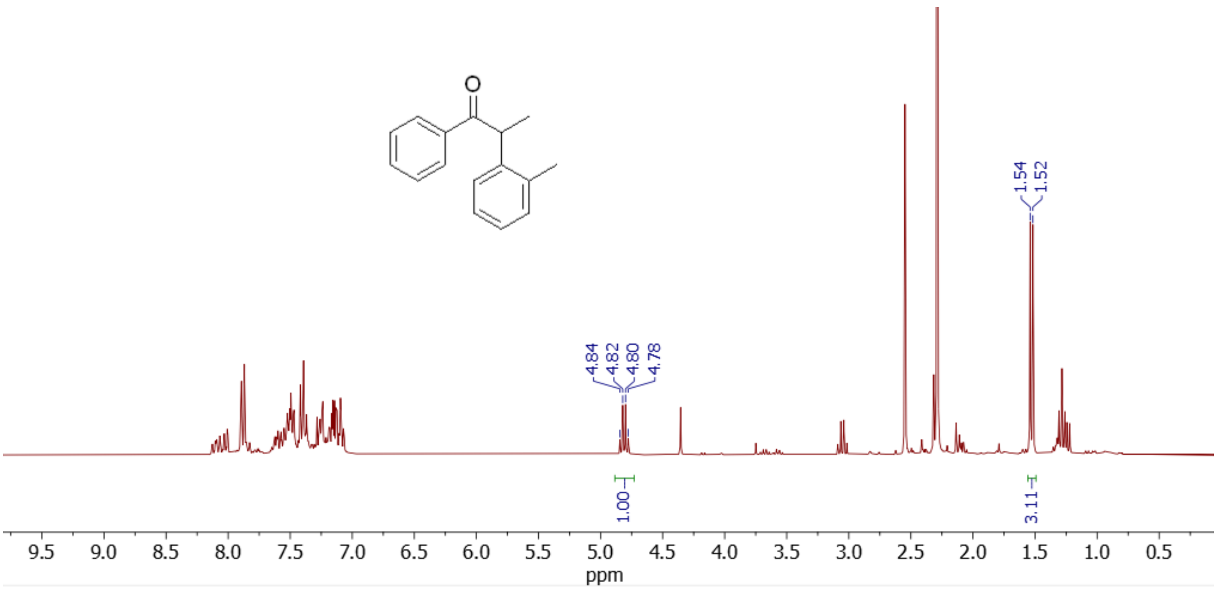

Figure S60. ^{1}H NMR spectrum ($CDCl_3$, 298 K, 300 MHz) of crude **11hc** from the reaction mixture, in agreement with literature data.[S11] Due to low conversion, only the aliphatic protons were integrated. Conversion was calculated from the crude mixture. HR-EI-MS (m/z): calculated for $C_{16}H_{16}O$ = 224.1201; found: 224.1195.

Product 11da

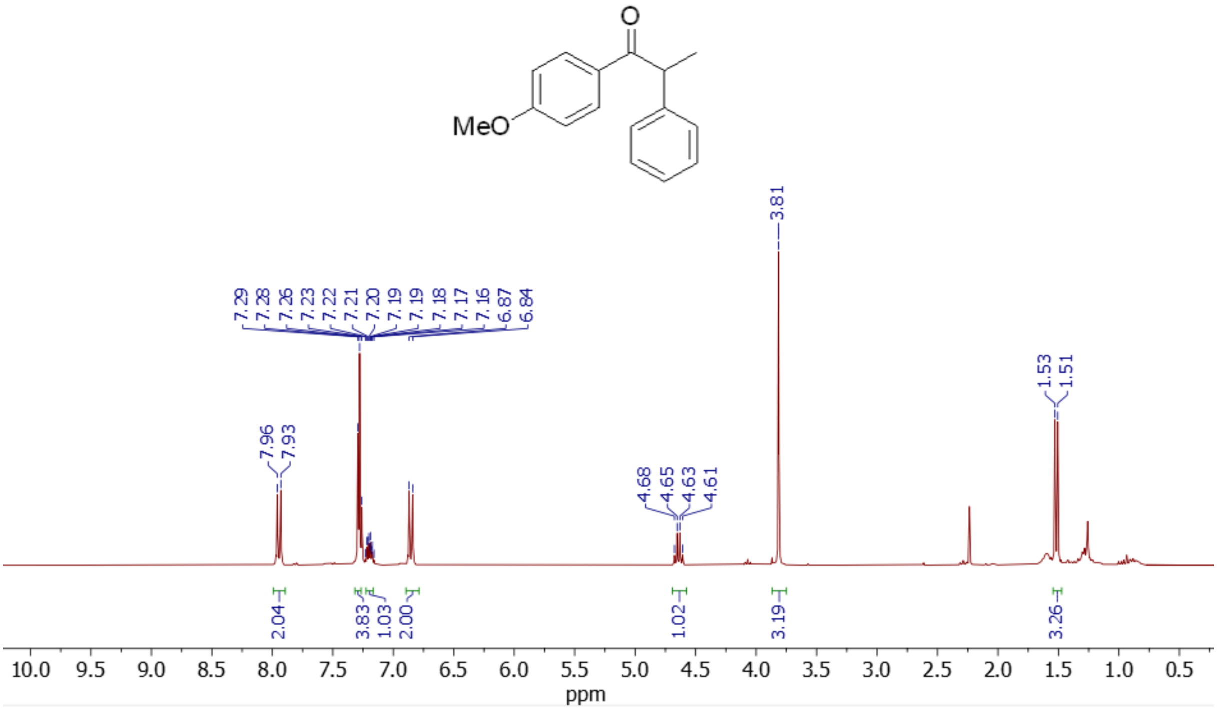

Figure S61. ^{1}H NMR spectrum ($CDCl_3$, 298 K, 300 MHz) of isolated **11da,** in agreement with literature data.[S12] HR-ESI-MS (m/z): calculated for $C_{16}H_{17}O_2$ $[M+H]^+$ = 241.1229; found: 241.1220.

Product 11db

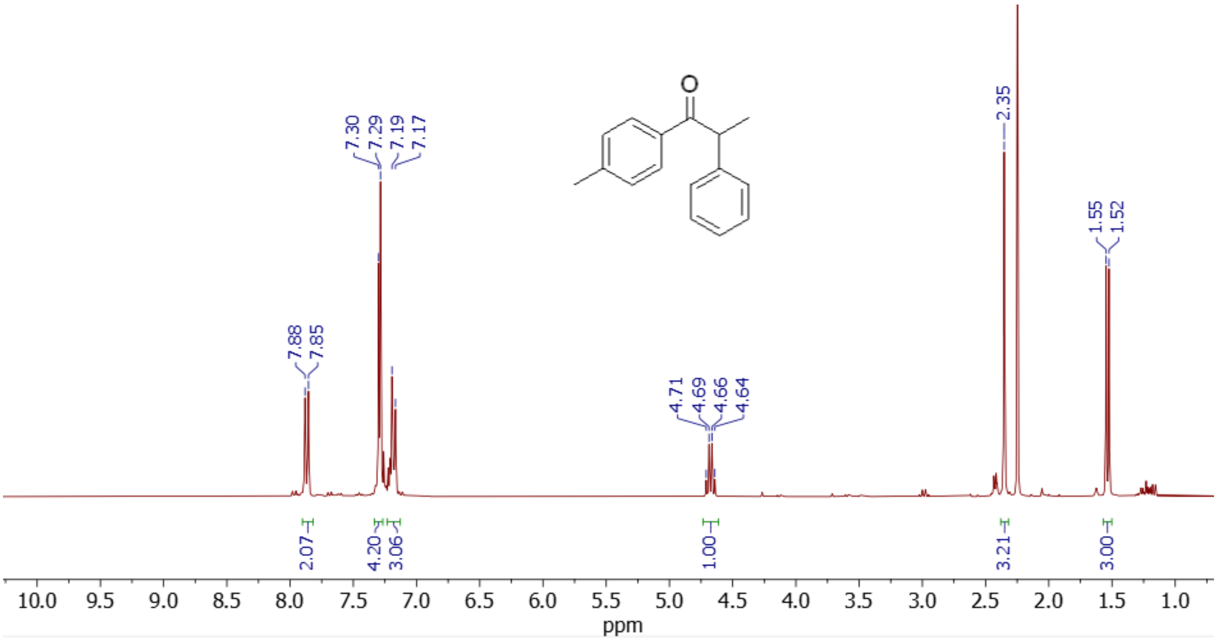

Figure S62. ^{1}H NMR spectrum ($CDCl_3$, 298 K, 300 MHz) of crude **11db** from the reaction mixture, in agreement with literature data.[S12] HR-ESI-MS (m/z): calculated for $C_{16}H_{17}O$ [M+H] = 225.1279; found: 225.1273.

Product 11dd

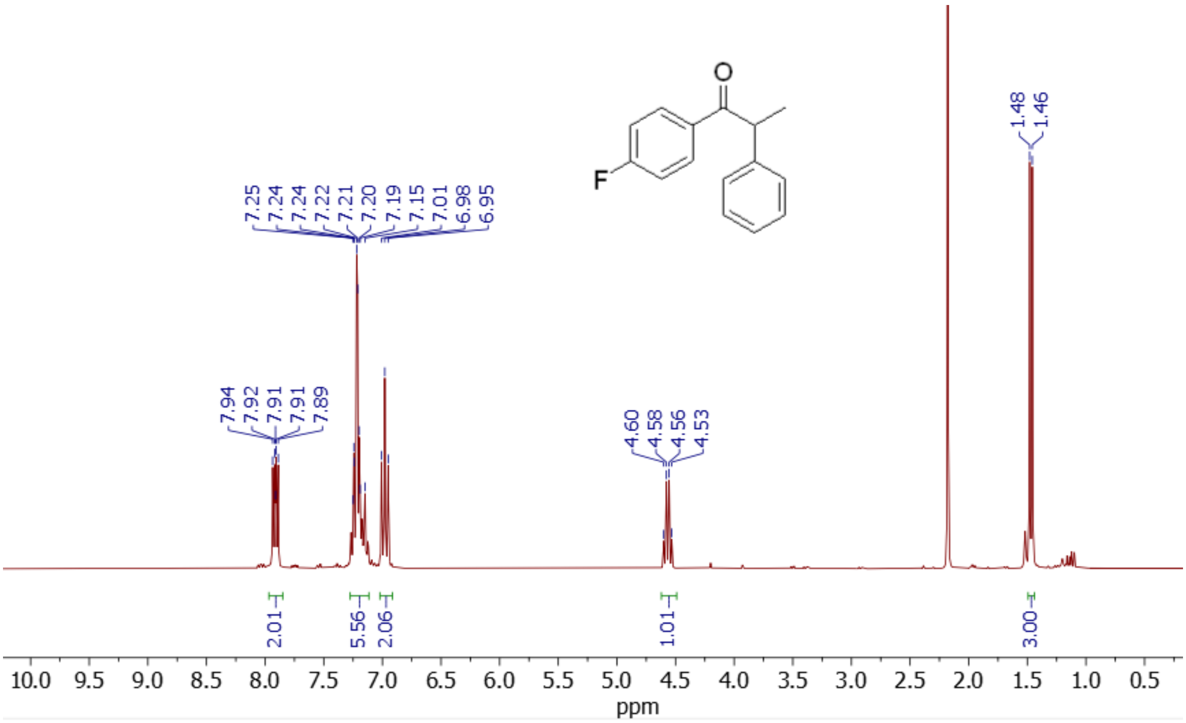

Figure S63. ^{1}H NMR spectrum ($CDCl_3$, 298 K, 300 MHz) of crude **11dd** from the reaction mixture, in agreement with literature data.[S12] HR-EI-MS (m/z): calculated for $C_{15}H_{13}FO$ = 228.0950; found: 241.1220.

Product 11de

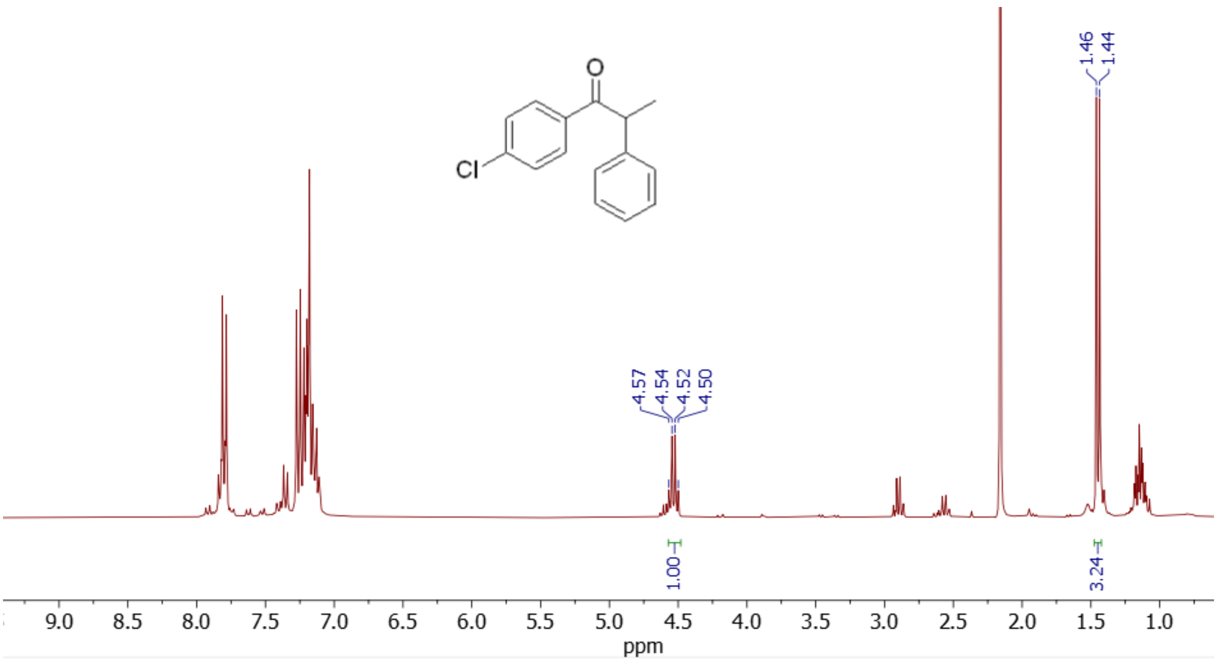

Figure S64. ^{1}H NMR spectrum ($CDCl_3$, 298 K, 300 MHz) of crude **11de** from the reaction mixture, in agreement with literature data.[S12] HR-ESI-MS (m/z): calculated for $C_{15}H_{13}ClO$ = 244.0655; found: 244.0653.

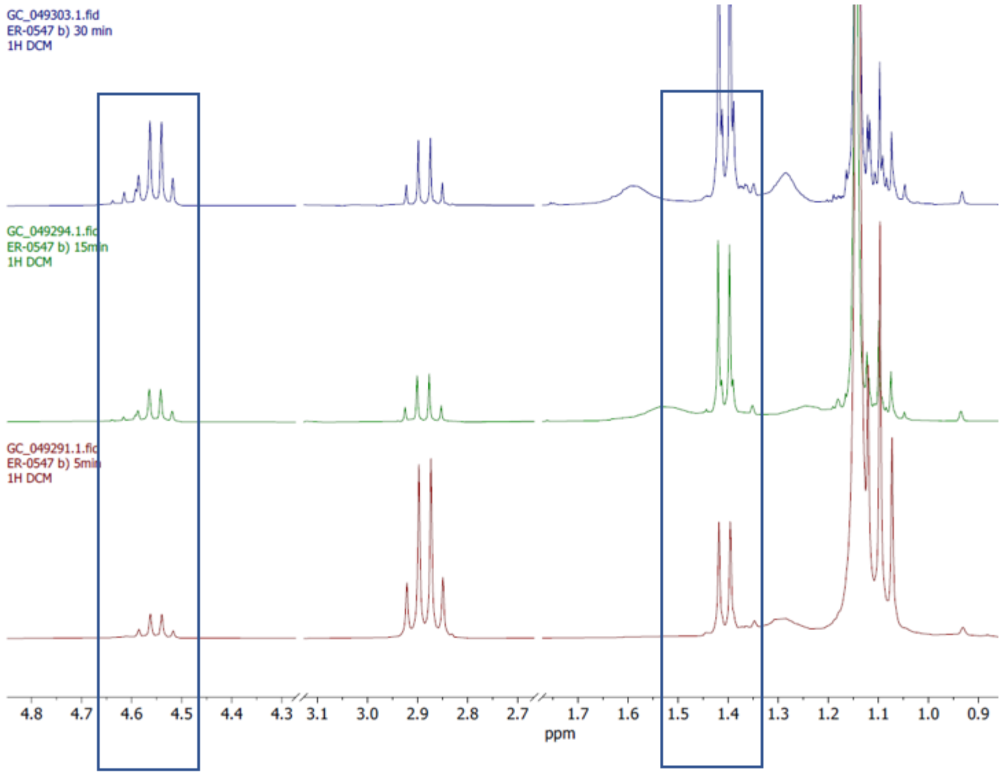

Figure S65. ^{1}H NMR spectrum ($CDCl_3$, 298 K, 300 MHz) of the crude product **11de** zoomed in to the two aliphatic resonances of the product showing the appearance of other minor overlapping signals attributed to aryl chloride activation.

Product 11df

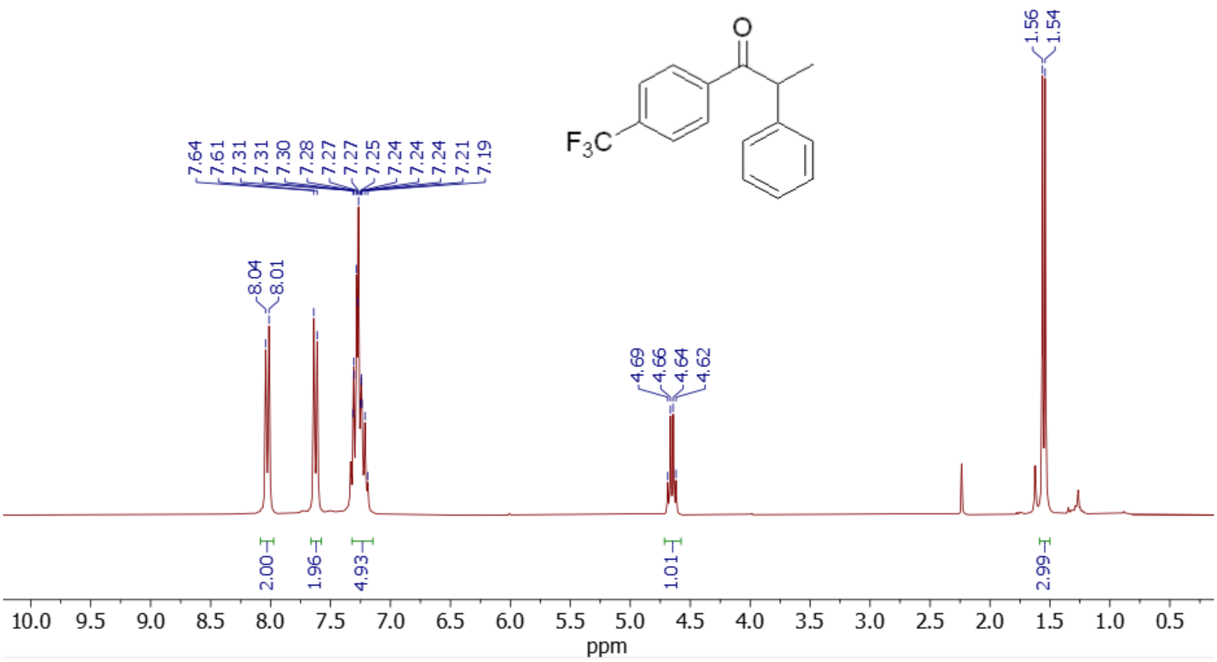

Figure S66. ^{1}H NMR spectrum ($CDCl_3$, 298 K, 300 MHz) of isolated **11df**, in agreement with literature data.[S14] HR-EI-MS (m/z): calculated for $C_{16}H_{13}F_3O$ = 278.0918; found: 278.0918.

Product 11dg

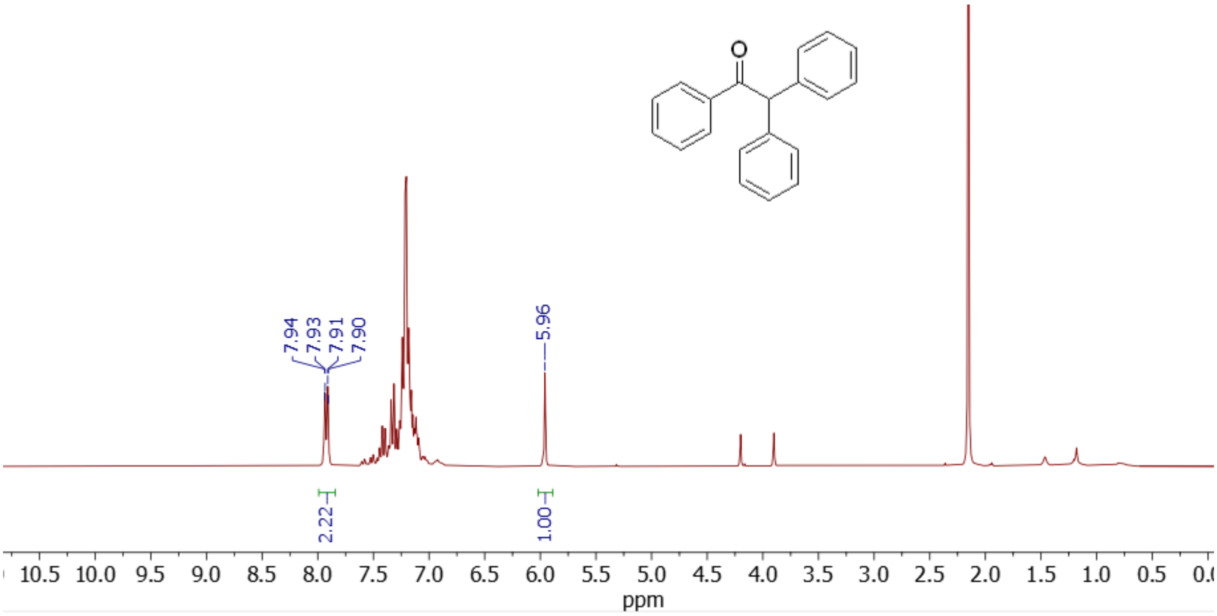

Figure S67. ^{1}H NMR spectrum ($CDCl_3$, 298 K, 300 MHz) of crude **11dg** from the reaction mixture, in agreement with literature data.[S15] Aromatic protons could not be integrated due to uncomplete conversion. HR-EI-MS (m/z): calculated for $C_{20}H_{16}O$ = 272.1201; found: 272.1198.

Product 11dh

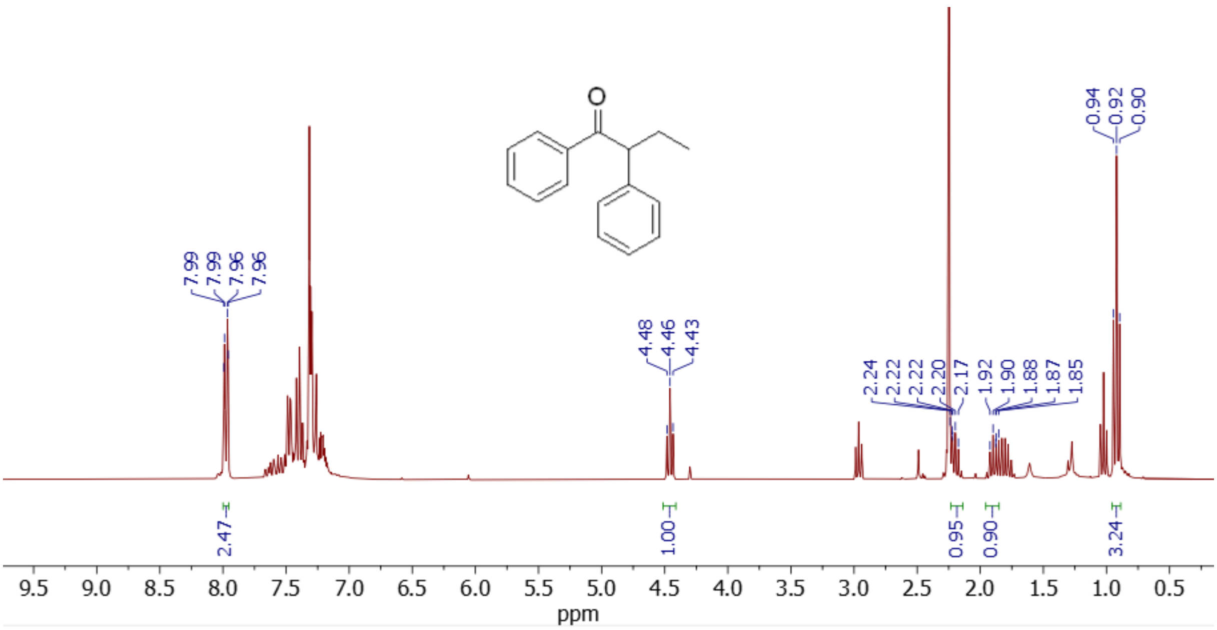

Figure S68. ^{1}H NMR spectrum ($CDCl_3$, 298 K, 300 MHz) of crude **11dh** from the reaction mixture, in agreement with literature data.[S16] Aromatic protons could not be integrated due to incomplete conversion. HR-ESI-MS (m/z): calculated for $C_{16}H_{17}O$ [M+H] = 225.1279; found: 225.1274.

Product 11di

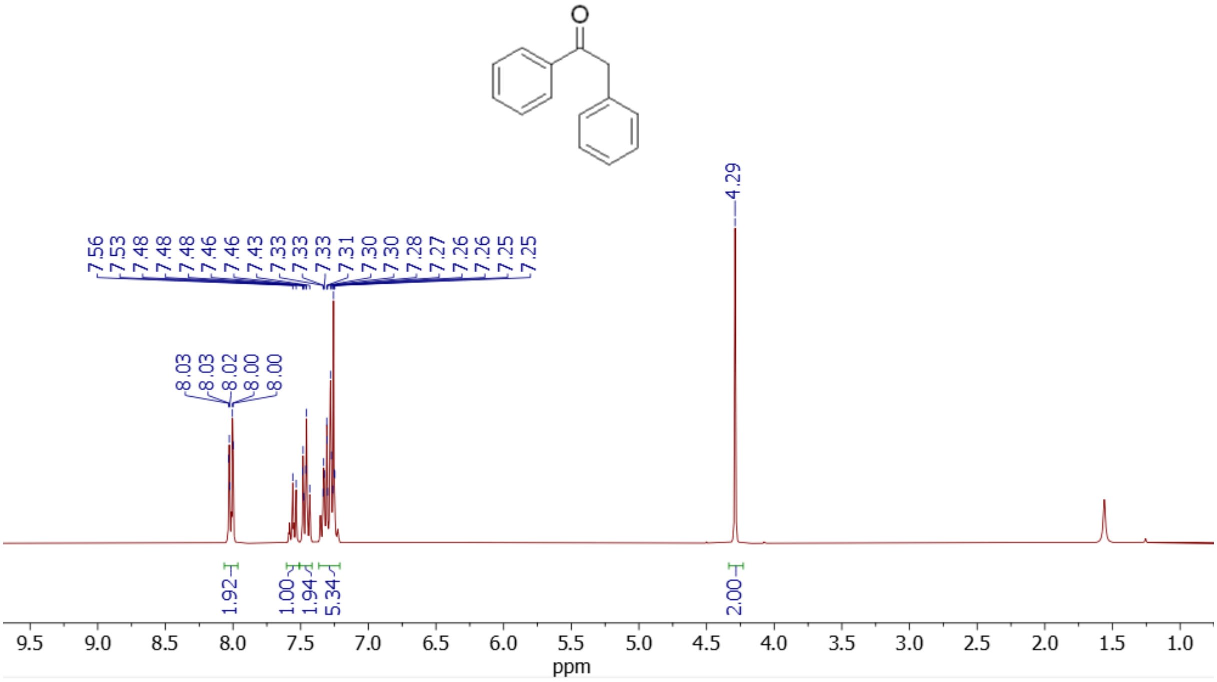

Figure S69. ^{1}H NMR spectrum ($CDCl_3$, 298 K, 300 MHz) of isolated **11di,** in agreement with literature data.[S17] HR-ESI-MS (m/z): calculated for $C_{14}H_{13}O$ [M+H] = 197.0966; found: 197.0959.

Product 11dj

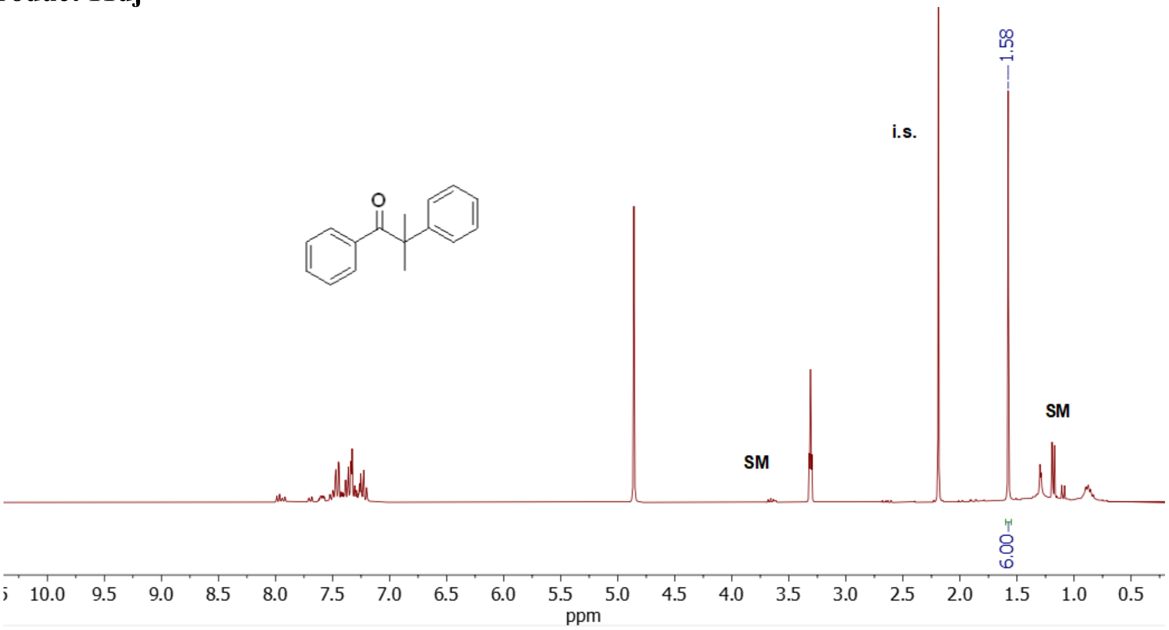

Figure S70. ^{1}H NMR spectrum (MeOD-*d4*, 298 K, 300 MHz) of crude **11dj** from the reaction mixture, in agreement with literature data.[S13] Aromatic protons could not be integrated due to incomplete conversion. Conversion and yield were assessed in a MeOD-*d4* solution to avoid potential overlap of the compound geminal methyls signal with H_2O in $CDCl_3$. HR-EI-MS (m/z): calculated for $C_{16}H_{16}O$ [M+H] = 224.1201; found: 224.1197.

Product 11dk

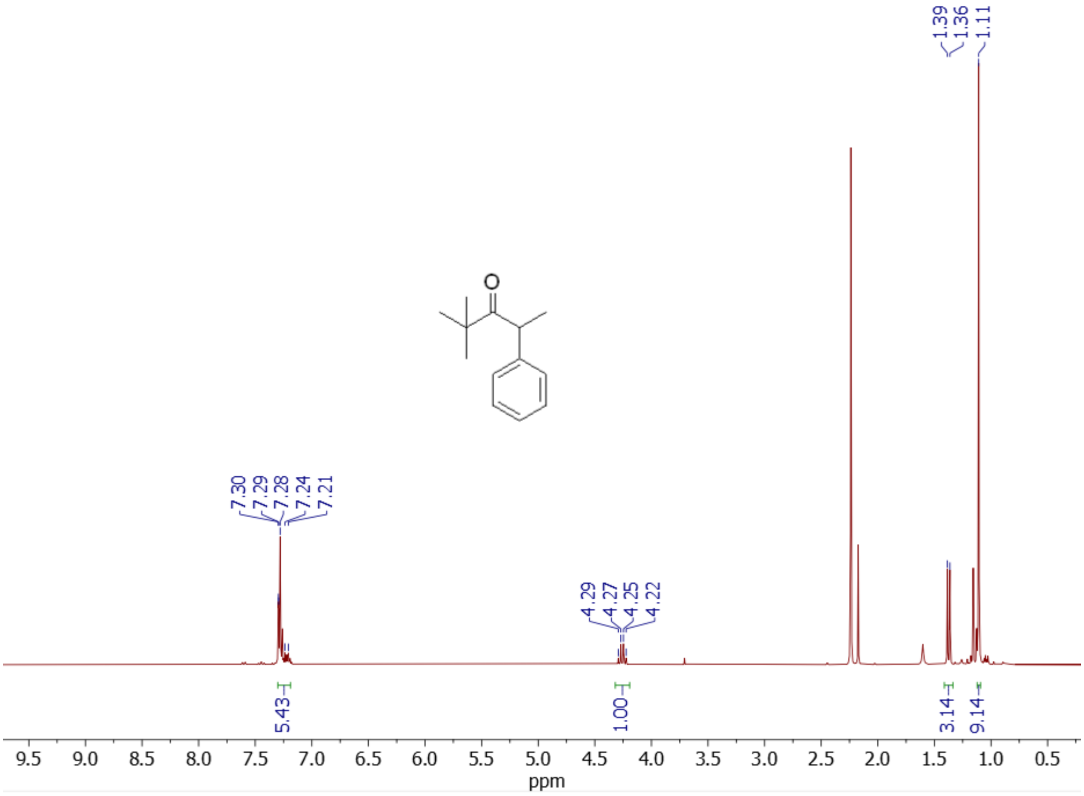

Figure S71. ^{1}H NMR spectrum ($CDCl_3$, 298 K, 300 MHz) of crude **11dk** from the reaction mixture, in agreement with literature data.[S18] HR-EI-MS (m/z): calculated for $C_{13}H_{18}O$ = 190.1358; found: 190.1352.

Product 11dl

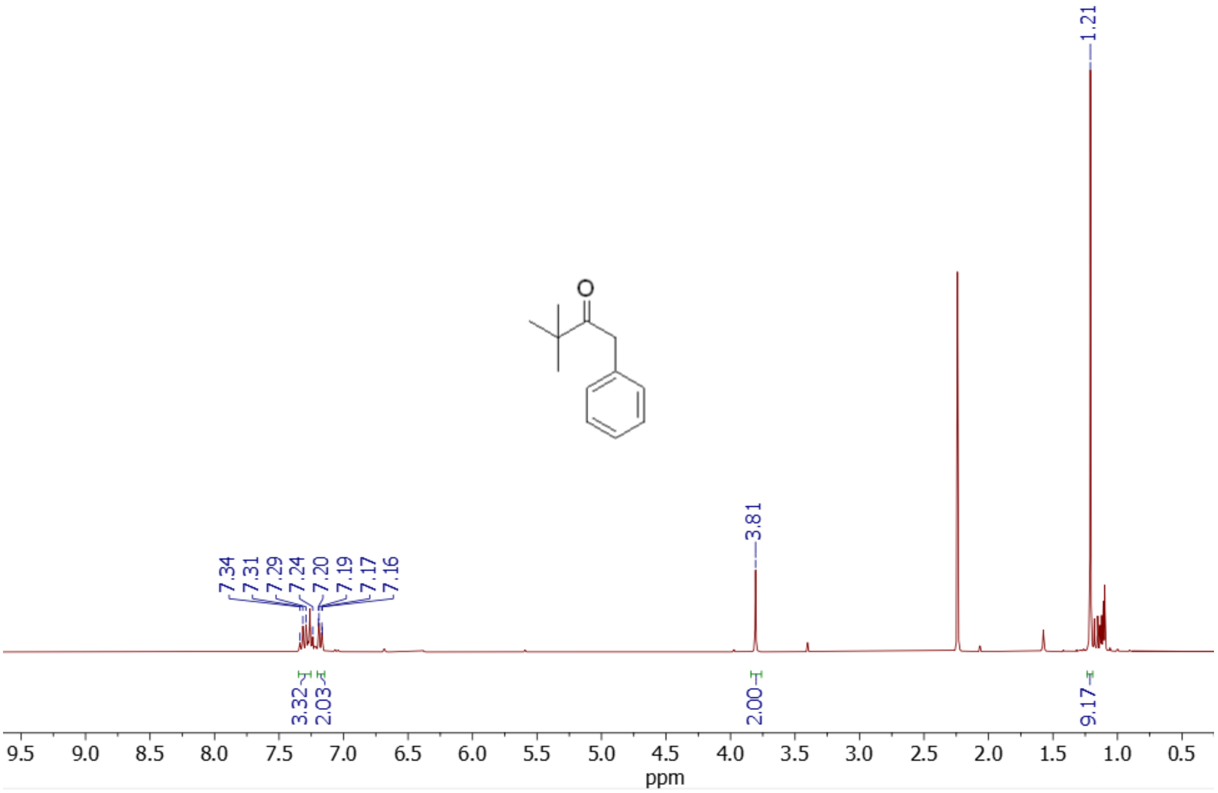

Figure S72. ^{1}H NMR spectrum ($CDCl_3$, 298 K, 300 MHz) of crude **11dl** from the reaction mixture, in agreement with literature data.[S19] HR-ESI-MS (m/z): calculated for $C_{12}H_{17}O$ [M+H] = 177.1279; found: 177.1272.

Product 11dm

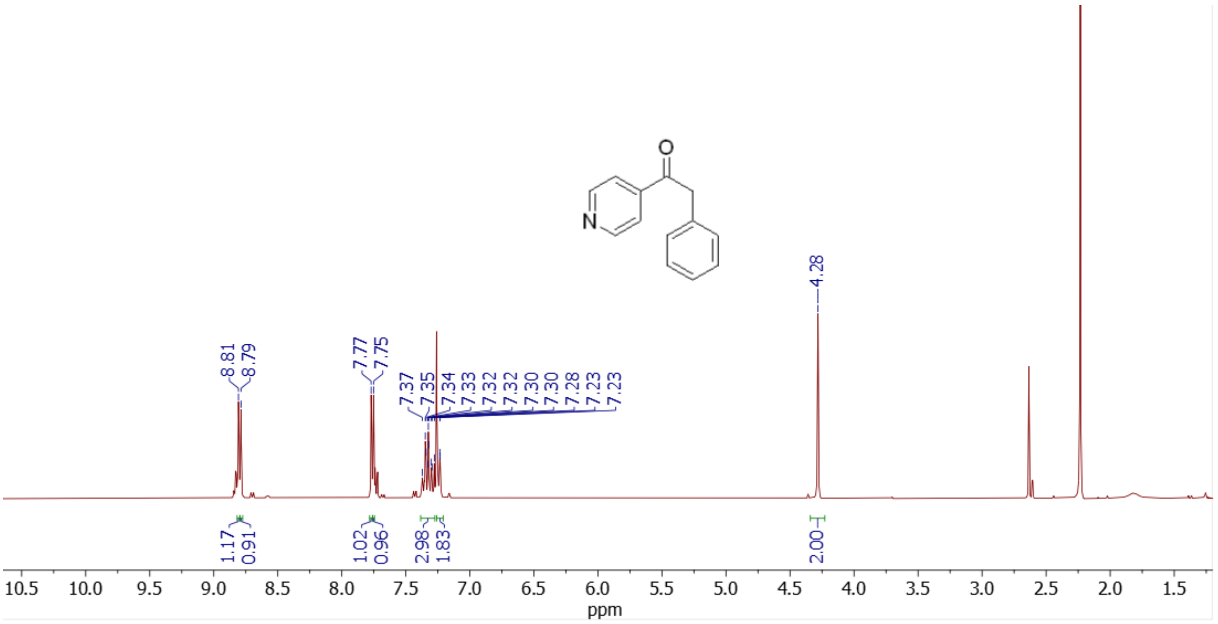

Figure S73. ^{1}H NMR spectrum ($CDCl_3$, 298 K, 300 MHz) of crude **11dm** from the reaction mixture, in agreement with literature data.[S20] HR-EI-MS (m/z): calculated for $C_{13}H_{11}NO$ = 197.0841; found: 197.0836.

S.5. References

S1 G. M. Ó Máille, A. Dall'Anese, P. Grossenbacher, T. Montini, B. Milani and M. Albrecht, *Dalton Trans.*, 2021, **50**, 6133–6145.

S2 P. D. W. Boyd, L. J. Wright and M. N. Zafar, *Inorg. Chem.*, 2011, **50**, 10522–10524.

S3 Oxford Diffraction (2018). CrysAlisPro (Version 1.171.40.37a). Oxford Diffraction Ltd., Yarnton, Oxfordshire, UK.

S4 G. M. Sheldrick, *Acta Crystallogr. Sect. C Struct. Chem.*, 2015, **A71**, 3–8.

S5 G. M. Sheldrick, *Acta Crystallogr. Sect. C Struct. Chem.*, 2015, **C71**, 3–8.

S6 O. V. Dolomanov, L. J. Bourhis, R. J. Gildea, J. a. K. Howard and H. Puschmann, *J. Appl. Crystallogr.*, 2009, **42**, 339–341.

S7 P. Macchi, H.-B. Bürgi, A. S. Chimpri, J. Hauser and Z. Gál, *J. Appl. Crystallogr.*, 2011, **44**, 763–771.

S8 L. Falivene, R. Credendino, A. Poater, A. Petta, L. Serra, R. Oliva, V. Scarano and L. Cavallo, *Organometallics*, 2016, **35**, 2286–2293.

S9 G. Lente, I. Fábián and A. J. Poë, *New J. Chem.*, 2005, **29**, 759.

S10 C. M. Zinser, K. G. Warren, F. Nahra, A. Al-Majid, A. Barakat, M. S. Islam, S. P. Nolan and C. S. J. Cazin, *Organometallics*, 2019, **38**, 2812–2817.

S11 J. M. Fox, X. Huang, A. Chieffi and S. L. Buchwald, *J. Am. Chem. Soc.*, 2000, **122**, 1360–1370.

S12 J. Templ and M. Schnürch, *J. Org. Chem.*, 2022, **87**, 4305–4315.

S13 M. Kawatsura and J. F. Hartwig, *J. Am. Chem. Soc.*, 1999, **121**, 1473–1478.

S14 M. Escudero-Casao, G. Licini and M. Orlandi, *J. Am. Chem. Soc.*, 2021, **143**, 3289–3294.

S15 A. Ehrentraut, A. Zapf and M. Beller, *Adv. Synth. Catal.*, 2002, **344**, 209–217.

S16 S. Jin, H. T. Dang, G. C. Haug, V. D. Nguyen, H. D. Arman and O. V. Larionov, *Chem. Sci.*, 2020, **11**, 9101–9108.

S17 S. Saha, S. Yadav, N. U. D. Reshi, I. Dutta, S. Kunnikuruvan and J. K. Bera, *ACS Catal.*, 2020, **10**, 11385–11393.

S18 C. H. Cheon, O. Kanno and F. D. Toste, *J. Am. Chem. Soc.*, 2011, **133**, 13248–13251.

S19 B. C. Hamann and J. F. Hartwig, *J. Am. Chem. Soc.*, 1997, **119**, 12382–12383.

S20 H. Wang, J. Mao, S. Shuai, S. Chen, D. Zou, P. J. Walsh and J. Li, *Org. Chem. Front.*, 2021, **8**, 6000–6008.
